# Suppressing circ_0008494 inhibits HSCs activation by regulating the miR-185-3p/Col1a1 axis

**DOI:** 10.3389/fphar.2022.1050093

**Published:** 2022-11-17

**Authors:** Binbin Li, Jiaming Zhou, Yuanyuan Luo, Kegong Tao, Lifen Zhang, Ying Zhao, Yong Lin, Xin Zeng, Hongyu Yu

**Affiliations:** ^1^ Department of Pathology, Second Affiliated Hospital of Naval Medical University, Shanghai, China; ^2^ Department of Pathology, Medical School of Nantong University, Nantong, China; ^3^ Department of Gastroenterology, Shanghai East Hospital, Tongji University School of Medicine, Shanghai, China; ^4^ Department of Traditional Chinese Medicine, Second Affiliated Hospital of Naval Medical University, Shanghai, China; ^5^ Department of Gastroenterology, Second Affiliated Hospital of Naval Medical University, Shanghai, China

**Keywords:** circ_0008494, hepatic fibrosis, hepatic stellate cells, miR-185-3p, Col1a1

## Abstract

**Background:** Hepatic fibrosis (HF) is characterized by activation of hepatic stellate cells (HSCs) and extensive deposition of extracellular matrix components, especially collagens. However, effective antifibrotic therapies are still lacking. Recently, circular RNAs (circRNAs) have been identified as novel regulators of HF.

**Methods:** circRNAs profile was screened by RNA sequencing and the location of circ_0008494 was confirmed by fluorescence *in situ* hybridization assay in human HF tissues. Bioinformatics analysis was used for result prediction and dual-luciferase reporter, together with AGO-RIP and biotin-coupled miRNA capture assays, were used to determine miR-185-3p/collagen type I alpha 1 chain (Col1a1) as the target of circ_0008494. A stable circ_0008494-interfering human HSCs cell line was constructed and used to determine the regulatory mechanism of circ_0008494/miR-185-3p/Col1a1 axis.

**Results:** circ_0008494 was abundantly and significantly over-expressed in human HF tissues and located at the cytoplasm of HSCs. Together, dual-luciferase reporter, AGO-RIP and biotin-coupled miRNA capture assays confirmed that circ_0008494 acted as a sponge of miR-185-3p. Cell functional experiments and rescue assays demonstrated suppressing circ_0008494 could inhibit activation, proliferation, migration of HSCs and promote their apoptosis through miR-185-3p. In particular, the HF indicator, Col1a1, was validated as the direct target of miR-185-3p and the suppression of circ_0008494 inhibited the expression of Col1a1 by releasing miR-185-3p.

**Conclusion:** Knocking down circ_0008494 inhibited HSCs activation through the miR-185-3p/Col1a1 axis. circ_0008494 could be a promising treatment target for HF.

## Introduction

Hepatic fibrosis (HF) is the central pathological process of various chronic liver diseases, including alcoholism, chronic viral hepatitis, autoimmune diseases and fatty liver (Lee et al., 2015). The prevalence of viral hepatitis is traditionally the leading cause of HF, and the rapid increase of metabolic liver diseases (e.g., NAFLD/NASH) in recent years has become a new risk factor for this disease (Chien et al., 2020; Zeng et al., 2021). Without appropriate intervention, HF can progress to liver cirrhosis, or even the lethal cancer hepatocellular carcinoma (HCC), which has been deemed a challenge to global health (Zhang et al., 2016). However, as of now, there is no effective treatment for HF. Hepatic stellate cells (HSCs) play a key role in the initiation of HF. When responding to injury or inflammatory stimuli, HSCs are activated to produce excessive extracellular matrix (ECM), including mainly collagen I and collagen III (Tsuchida and Friedman, 2017). Thus, the strategies to inhibit the HSCs activation and collagens deposition are critical to the prevention and treatment of HF.

In recent years, studies have revealed that non-coding RNAs cooperate in fibrogenesis and are correlated with the severity of HF. These include microRNAs (miRNAs), long non-coding RNAs (lncRNAs) and circular RNAs (circRNAs) (Song et al., 2018; Ghafouri-Fard et al., 2021; Yang et al., 2021). circRNA is a novel type of non-coding RNA characterized by a covalent closed-loop structure without 5′ to 3′ ends or a poly-A tail. It has high stability, evolutionary conservation and tissue specificity (Jeck and Sharpless, 2014; Lasda and Parker, 2014). circRNAs can be divided into three types: ecircRNAs (exonic circRNAs), ciRNAs (intronic circRNAs) and EIciRNAs (exon-intron circRNAs). EIciRNAs/ciRNAs are reported to be mainly involved in gene transcription and are usually located in the cell nucleus (Jeck et al., 2013; Li Z et al., 2015; Zhang et al., 2013). Conversely, most ecircRNAs are commonly found in the cell cytoplasm. They contain miRNA response elements (MREs) and act as competitive endogenous RNAs (ceRNAs) by sponging miRNAs (Zheng et al., 2016; Bu et al., 2021).

Studies have found that circRNA could act as a diagnostic marker and serve as a potential therapeutic target in non-alcoholic fatty liver disease, nonalcoholic steatohepatitis, hepatitis and hepatocellular carcinoma (Han et al., 2017; Jin X. et al., 2020; Chien et al., 2020; Chen et al., 2021; Sunagawa et al., 2021; Wang et al., 2019). Recently, circRNAs are gaining increasing attention in the field of HF. However, the exact mechanism of circRNA and its ceRNA networks in HF remain unclear. Therefore, further research to illuminate the role of circRNAs in HSCs activation and HF is warranted.

In this research, it is first discovered that circ_0008494 served as a new regulator in HF. circ_0008494 regulated the activation of HSCs by sponging miR-185-3p and particularly targeting collagen type I alpha 1 chain (Col1a1), thus it may serve as a promising therapeutic target of HF.

## Materials and methods

### Patient samples

A total of twenty-eight HF tissues (distal paracancerous tissue from patients undergoing hepatectomy for HCC) and twenty-two normal liver tissues (distal normal tissues from patients undergoing hepatic hemangioma resection) were obtained from the Second Affiliated Hospital of Naval Medical University, Shanghai, China. All liver tissues were identified by two senior professional pathologists. The staging of HF was determined based on the Metavir semi-quantitative evaluation system. Six human HF tissues were used for RNA sequencing (RNA-seq). In addition, 22 liver cirrhosis (advanced HF tissue) were used for further detection of the expression of hsa_circ_0008494. The research was approved by the Research Ethics Committee of Second Affiliated Hospital of Naval Medical University, Shanghai, China. Informed written consent was obtained from each sampled patient.

### RNA-seq array

Six HF tissues were used for RNA-seq detection. The clinical information of each subjected sample was shown in Supplementary Table S1. RNA QC and quantitation was performed using the Agilent Bio-analyzer 2100 **(** Supplementary Table S2 **)**. Sequencing and data analysis were performed using the Illumina HiSeq 4000 system (Illumina, San Diego, CA, United States). The experimental process was carried out according to the standard procedures provided by Illumina, including the preparation of library and sequencing experiment. Ribosomal RNA (rRNA) was removed through epicenter ribo zero^TM^kit produced by the Illumina company after the quality inspection. The remaining RNA was purified, recovered and randomly broken into small pieces by fragmentation buffer for RNA library construction. The reading length is 2 × 150 bp (PF150). The circRNAs sequencing data has been successfully uploaded on the GEO website (https://www.ncbi.nlm.nih.gov/geo/query/acc.cgi?acc=GSE191247).

### Cell culture and TGF-β1 cytokine stimulation

The human HSC cell line LX-2 was cultured in Dulbecco’s modified Eagle’s medium, supplemented with 100 U/ml penicillin, 100 mg/ml streptomycin (Beyotime, Shanghai, China) and 10% fetal bovine serum (Gibco, New York, NY, United States) at 37°C in an atmosphere of 5% CO_2_.

Recombinant human TGF-β1 (PeproTech, NJ, United States) was used to activate LX-2 cells. Briefly, the LX-2 cells in the logarithmic growth phase were seeded in culture dishes at a suitable density. After starvation with serum-free DMEM medium, TGF-β1 (15 ng/ml) was added and stimulation was performed for 24 hours.

### Actinomycin D (ActD) and RNase R treatment assay

Transcription blocking assay was performed through the addition of 2 μg/ml actinomycin D (Med Chem Express, NJ, United States) for 4, 8, 12, and 24 h. To detect the expression level of circ_0008494 and linear ARID1A mRNA, the same amount of RNA was utilized for reverse transcription and quantitative real-time PCR analysis. The RNase R treatment assay was used to verify the stability of circ_0008494. Brefily, 1 μg of total RNA was incubated with RNase R (Epicenter Technologies, Madison, WI, United States) for 30 min at room temperature. For the RNase R treatment group, 0.15 μl RNase R (20 U/μl)and 1.5 μl 10×reaction buffer were added. As for the control group, 0.15 μl DEPC-treated water and 1.5 μl 10×reaction buffer were added.

### HE、Masson and immunohistochemical (IHC) staining

Proper amounts of human HF tissues were fixed in 10% formalin, embedded in paraffin, and cut to 4-μm-thick slices. Histopathology evaluation was conducted by HE, Masson and IHC staining. HE and Masson trichrome staining were performed with the HE staining kit (Beyotime, Beijing, China) and Trichrome Stain (Masson) Kit (Sigma-Aldrich, United States). For immunohistochemical staining, sections were deparaffinized and rehydrated routinely. Then the sections were placed in citrate buffer and heated to boiling for antigen retrieval. Sections were blocked with 5% BSA for 1 h at room temperature and incubated with the following antibodies: rabbit anti-α-SMA, rabbit anti-Col1a1(CST, MA, United States) overnight at 4°C. The next day, sections were incubated with the goat-anti-rabbit secondary antibodies at 37°C for 1 h. Staining was visualized using a DAB solution for 10 min at room temperature and hematoxylin counterstain.

### Fluorescence *in Situ* hybridization

The expression level of hsa_circ_0008494 in liver tissues was evaluated by fluorescence *in situ* hybridization (FISH) assay using a specific Cy3-labeled-circ_0008494 probe (circ103134, Ruibo Bio, Guangzhou, China) and a Fluorescent *in Situ* Hybridization Kit (C10910, Ruibo Bio, Guangzhou, China). Following the manufacturer’s instructions, pre-hybridization solution was added to the sections and incubated at 37°C for 30 min. After pre-hybridization, tissue sections were hybridized in hybridization buffer with Cy3-labeled-circ_0008494 probe overnight at 37°C. The next day, sections were washed sequentially with hybrid wash buffer I, II, III at 42°C for 5 min each time under the condition of darkness. Finally, the sections were counterstained by 4′,6-Diamidino-2-phenylindole (DAPI) and observed under the immunofluorescence microscope (Zeiss, Thornwood, United States).

### Quantitative real-time PCR (qRT-PCR)

Detection of mRNAs were performed using a qPCR-RT Kit and a SYBR Premix Ex TaqTM II Kit (RR036A, RR820A, Takara, Tokyo, Japan). Detection of circRNAs was performed using Reverse transcription and qRT-PCR kits (R11088.2, Ruibo Bio, Guangzhouou, China). Detection of miRNAs was performed using Reverse transcription and qRT-PCR kits (R10031.7, Ruibo Bio, Guangzhou, China). The primers used for detection of mRNAs and circRNAs are shown in Supplementary Tables S3, S4. miR-185-3p primer, miRNA mimic, miRNA inhibitor and miRNA NC were designed by Ruibo Bio (Guangzhou, China). The mRNA and circRNA levels were normalized to total GAPDH. The miRNA level was normalized to U6.

### Western blot analysis

RIPA-PMSF (100:1) buffer (Beyotime, Shanghai, China) was used for cell lysis, and the lysates were separated by SDS-PAGE. Proteins of 10–20 mg were transferred to polyvinylidene fluoride membranes (Millipore, CA, United States) at 350 mA for 2 h. The membranes were blocked using 5% BSA at room temperature for 1 h and incubated using the primary antibodies overnight at 4°C. The primary antibodies worked in this paper included anti-α-SMA, anti-Col1a1, anti-FGF5 and anti-BRD4 antibodies (rabbit anti-human, CST, MA, United States). After incubation with the goat-anti-rabbit secondary antibodies (Sigma, CA, United States) for 2 h, the membranes were subjected to chemiluminescence exposure and photographed using a Tannon 3500 imager (Tannon, Shanghai, China). The protein levels were normalized to total GAPDH.

### Establishment of a stable circ_0008494-interfering cell line

Three siRNAs targeting the back-splice site of hsa_circ_0008494 and a negatively controlled siRNA were designed and packaged into the lentiviral vector (No. Gv493, element sequence hu6-mcs-cbh-gcgfp-ires-puromycin). The negatively controlled scramble sequence was TTC​TCC​GAA​CGT​GTC​ACG​T. LX-2 cells were infected with lentiviral constructs, including LV-circ_0008494-KD1, LV-circ_0008494-KD2, LV-circ_0008494-KD3 and LV-NC. The GFP labeling of the recombinant viruses was confirmed using an inverted fluorescent microscope (Zeiss, Thornwood, United States) and the knockdown efficiency of circ_0008494 was confirmed by RT-qPCR assays. A stable circ_0008494-interfering cell line and a stable LV-NC cell line were established following culture in the presence of puromycin (5 μg/ml). The cells were used for functional and the rescue experiments.

### miRNA transfection

miRNA products and transfection reagent were purchased from Ruibo Bio (Guangzhou, China). Transfection was carried out according to the manufacturer’s instructions. Briefly, 10 × riboFECTTM CP Buffer was diluted to 1 ×. Then 120 μl 1 × RiboFECTTM CP Buffer and 10 μl miRNA product were added to six well plates. The mixture was incubated for 5 min at room temperature. Subsequently, 12 μl riboFECTTM CP Agent was added into the mixture and incubated for 10–15 min. The final transfection concentration was 125 nM. For cell function detection or rescue assays, miRNA products were respectively transfected into LX-2 cells, stable LV-circ_0008494-KD LX-2 cells or LV-NC cells according to the experimental purpose. 48 h after transfection, the cells of each experimental group were re-suspended, collected and subjected to downstream experiments.

### CCK8 assay

Cells of each experimental group were seeded in 96-well plates overnight at a density of 1 × 10^3^/ml. After 1, 2, 3, 4 or 5 days, 10 μl CCK8 (Dojindo, Kumamoto, Japan) was added to each well and incubated for 4 h at 37°C. The absorbance was measured with a microplate reader at 450 nm.

### Transwell migration assay

Cells of each experimental group were resuspended and seeded in upper transwell chambers with pore size of 8 µm for the migration assay (Corning, NY, United States) at a density of 1 × 10^6^ cells/ml. The cells were allowed to cross the chamber for 48 h. The penetrated cells were fixed, stained with 1% crystal violet, and counted under an inverted microscope at a magnification ×100 (Zeiss, Thornwood, United States).

### Apoptosis analysis

Cell apoptosis was quantified using an Annexin V-APC Single Staining Kit (eBioscience, San Diego, CA, United States). The experimental grouping was carried out as above. Each Falcon tube was added with Annexin V-APC (10 μl) and incubated at 37°C in the dark for 10–15 min. Next, each tube was added with 400 μl 1× Binding Buffer. Apoptosis was detected within 1 h in a flow cytometer (Beckman Coulter, CA, United States).

### Dual-luciferase reporter assay

To detect the binding of hsa_circ_0008494 and has-miR-185-3p, wild-type of circ_0008494 or its mutant sequence was inserted into the psiCHECK™ vector. circ_0008494-WT-psiCHECK or circ_0008494-MUT-psiCHECK was co-transfected with miR-185-3p mimic and mimic NC (miR-185-3p inhibitor and inhibitor NC) using Lipofectamine 2000 (Invitrogen, CA, United States) in the HEK293T cells. Additionally, to detect the binding of Col1a1 and hsa-miR-185-3p, the Col1a1 3′UTR or its mutant sequence was inserted into the pMIR-REPORT vector. miR-185-3p inhibitor and inhibitor NC were co-transfected with Col1a1 3′UTR-WT-pMIR or Col1a1 3′UTR-MUT-pMIR vectors. 48 h since the transfection, the relative luciferase absorbance value of each group was examined with a Dual-Luciferase Reporter Assay System (Promega, Madison, United States).

### AGO-RIP assay

A Simple Chip™ Enzymatic Chromatin IP Kit (CST, MA, United States) was used for RNA immunoprecipitation (RIP) experiments. 1 × 10^7^ LX-2 cells were pelleted and re-suspended with an equal pellet volume of RIP Lysis Buffer. After that, 100 μl of cell lysate was incubated with RIP buffer containing magnetic beads conjugated with human anti-AGO2 antibody or isotype-matched IgG (CST, MA, United States) at 4°C overnight. The immunoprecipitated RNAs were extracted by RNeasy MinElute Cleanup Kit (Qiagen, Hilden, Germany) after the treatment of proteinase K on the next day. Finally, collected RNA was reversed into cDNA and the abundance of circ_0008494 and miR-185-3p was evaluated by qPCR analysis.

### Biotin-coupled miRNA capture

A total of 1 × 10^7^ LX-2 cells were harvested, lysed and sonicated. The biotin-coupled miR-185-3p probe or miRNA NC probe were incubated with the C-1 magnetic beads (Life Technologies, Guilford, CT) at 25°C for 2 h to generate probe-coated beads. The cell lysates were incubated with miR-185-3p probe or miRNA NC probe at 4°C overnight. After cleansing with the wash buffer, the RNA complexes bound to the beads were eluted and extracted by RNeasy MinElute Cleanup Kit (Qiagen, Hilden, Germany) for qRT-PCR assay on the next day. Biotin-coupled miR-185-3p probe was designed and synthesized by Ruibo Bio (Guangzhou, China).

### Statistical analysis

Statistical analyses were performed using SPSS software 25.0 and GraphPad Prism 8.0. Statistically significant differences were calculated using Student’s *t*-test. Data were expressed as the mean ± SD of three independent experiments and *p* < 0.05 was considered to indicate a significant difference. The asterisks ^*^, ^**^and^***^ stand for *p* < 0.05, *p* < 0.01and *p* < 0.001 respectively.

## Results

### Identification of circRNAs in human HF tissues

**FIGURE 1:**
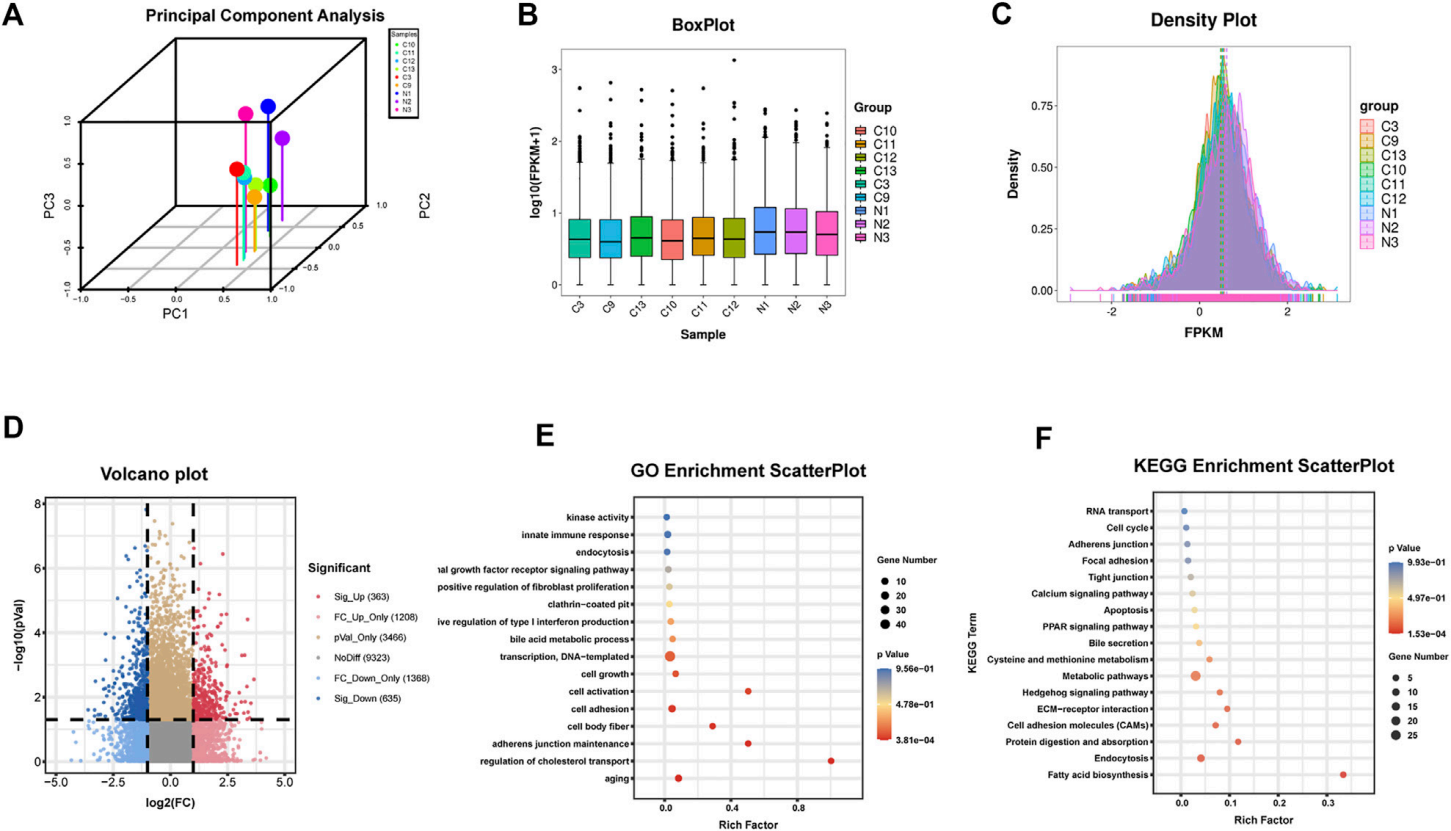
Identification of circRNAs in human HF tissues **(A)**Principal component analysis **(B)** Box plot **(C)** Density plot **(D)** Differential circRNAs volcano plot. The abscissa is the log2 (Fold Change), and the ordinate is -log10 (*p*-value). circRNAs with fold changes ≥2 and *p*-values ≤ 0.05 are indicated as significant up-regulation **(E)** GO_enrichment_scatterplot of the parental genes of the upregulated circRNAs **(F)** KEGG_enrichment_scatterplot of the parental genes of the upregulated circRNAs.C represents HF samples (*n* = 6). N represents control samples (*n* = 3).

Six human HF samples were used for RNA-seq detection. All RNA samples were of high-quality with a 28S/18S ratio ≥1.0 and RIN ≥7.0. The results of circRNAs sequencing were analyzed using the Illumina HiSeq system 4000, together with the data of three normal liver tissues searched on NCBI as controls (https://www.ncbi.nlm.nih.gov/Traces/study/?acc=ERP013191, data from the Cambridge Institute University of Cambridge cancer research, United Kingdom). Finally, 363 significantly upregulated and 635 significantly downregulated circRNAs were detected in human HF tissues (Figures 1A–D).

The parental genes of the upregulated and downregulated circRNAs were analyzed by GO and KEGG enrichment analysis, respectively. Among the upregulated circRNAs, the enriched factors were primarily related to aging, cell body fiber, cell activation, or positive regulation of fibroblast proliferation in GO analysis while in KEGG analysis, the enriched factors were mainly related to fatty acid biosynthesis, ECM-receptor interaction, or focal adhesion (Figures 1E, F). Among the downregulated circRNAs, the enriched factors were primarily related to myofibril, collagen fibril organization, or smooth muscle contraction in GO analysis while in KEGG analysis, the enriched factors were mainly related to ECM-receptor interaction, Focal adhesion or TGF-beta signaling pathway **(** Supplementary Figure S1). The analysis preliminarily indicated that the dysregulated circRNAs obtained in this RNA-seq might have a potential function in the regulation of HF.

### Identification and characteristics of circ_0008494 in HF tissues and HSCs

**FIGURE 2:**
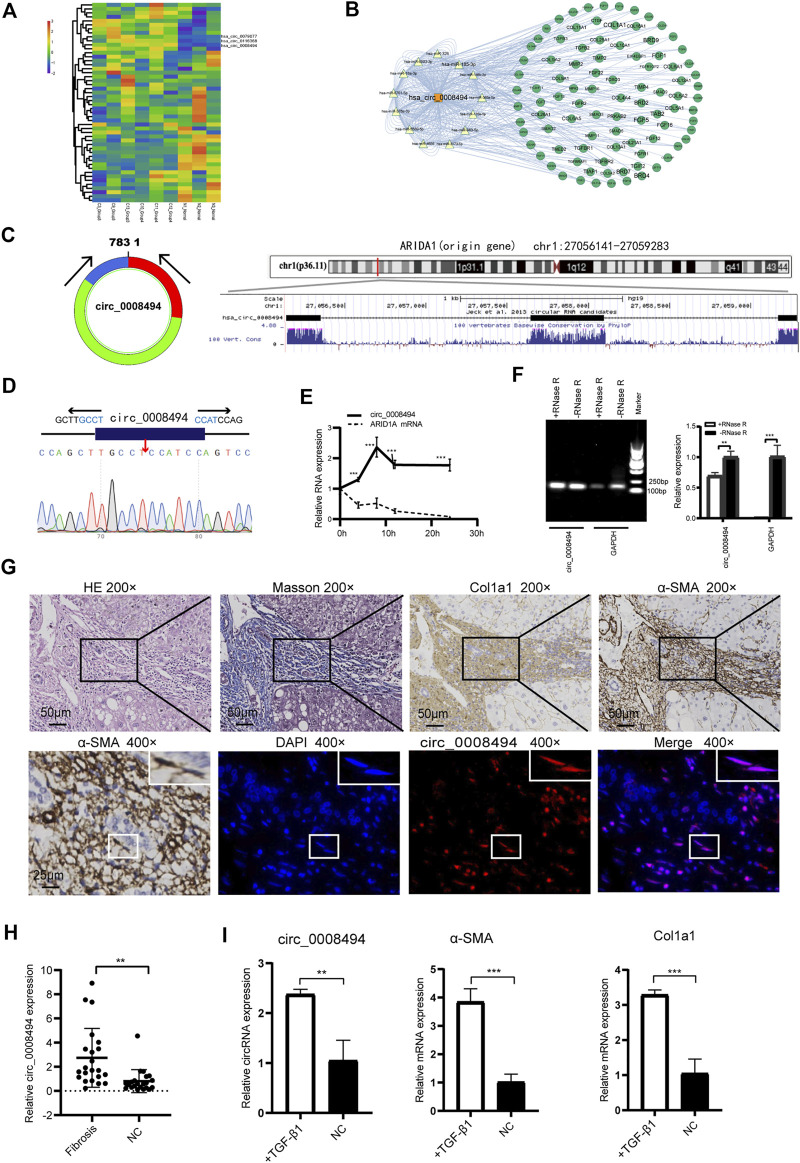
Identification and characteristics of circ_0008494 in HF tissues and HSCs **(A)** circRNAs clustering heat map showed circ_0008494 was up-regulated in HF tissues. C represents HF samples (*n* = 6). N represents control samples (*n* = 3). High expression level is indicated by “red” and lower levels by “blue” **(B)** circRNA-miRNA-mRNA interaction network of circ_0008494**(C)** circ_0008494 is derived from the second, third and fourth exons of the ARIDIA gene, which is located on chromosome 1p36 **(D)** The back-splice junction of circ_0008494 was verified by Sanger sequencing **(E)** ActD treatment assay showed circ_0008494 was more stable and resistant to ActD than the linear ARID1A mRNA **(F)** Electrophoresis on agarose gel and qRT-PCR assays showed circ_0008494 exhibited clear resistance to RNase R digestion **(G)** FISH assay was conducted on the HF tissue (nodular cirrhosis). Fibrotic area was confirmed by HE, Masson and immunohistochemical staining (Col1a1, α-SMA) and indicated with the black boxes (×200) (Low-magnification were shown in Supplementary Figure S2). HSCs (α-SMA-positive, 400×, representative cells were indicated with the white boxes) with a spindle-shaped fibroblastic morphology were present in large numbers in fibrous septa, followed by some small bile ducts. FISH assay revealed that circ_0008494 was predominantly localized at the cytoplasm of HSCs (400×), but not expressed in adjacent cholangiocytes. Cy3-labeled-circ_0008494 appeared “red”, and DAPI staining nuclei appeared “blue” **(H)** qRT-PCR assay showed circ_0008494 significantly increased in human HF tissues (*n* = 22) compared with paired normal tissues **(I)** qRT-PCR assay showed circ_0008494 significantly increased in TGF-β1 activated LX-2 cells, together with the HF indictors a-SMA and Col1a1. The RNA levels were normalized to total GAPDH. Statistical analysis: Student *t*-tests. ^**^ and ^***^stand for *p* < 0.01 and *p* < 0.001, respectively. Experiments were repeated independently three times.

Three dysregulated circRNAs, hsa_circ_0079077, hsa_circ_0116368 and hsa_circ_0008494 which were uniformly upregulated in the six HF sequencing samples, were initially noted (Figure 2A, Supplementary Table S4). miRNAs and mRNAs profiles were also screened using the same batch of HF specimens (datasets are available at: https://www.ncbi.nlm.nih.gov/geo/query/acc.cgi?acc=GSE190366) in our RNA-seq. Through miRanda algorithms, circRNA-miRNA-mRNA interaction network of the three circRNAs in the sequencing data was calculated. Multiple HF related genes (particularly numerous collagens) were found to be located at the circRNA-miRNA-mRNA network of circ_0008494 (Figure 2B). However, circ_0079077 and circ_0116368 were not involved in the transcription of ECM components that much. Thus, major attention was preliminarily drawn to circ_0008494.

Using the UCSC Genome Browser**,** circ_0008494 was found derived from exon2, exon3 and exon4 of the ARID1A (AT-rich interaction domain 1A, located on chromosome 1p36) gene through back-splicing (Figure 2C). The splice junction of circ_0008494 was verified by Sanger sequencing in human HSC cell line LX-2 (Figure 2D). To further confirm the circular characteristics of circ_0008494, ActD, an inhibitor of transcription, was added in LX-2 cells. The linear ARID1A mRNA was used as a control in this experiment. Total RNA was harvested at the indicated time points (0,4,8,12 and 24 h) after treatment with 2 μg/ml ActD.qRT-PCR showed circ_0008494 was more stable and resistant to ActD than the linear ARID1A mRNA (Figure 2E). Besides, an RNase R digestion assay was performed and the result further demonstrated that circ_0008494 was well resistant to RNase R digestion (Figure 2F).

The FISH assay was performed in HF tissue using the Cy3-labeled-circ_0008494 probe. Fibrotic area was confirmed by HE, Masson and immunohistochemical staining (Col1a1, α-SMA) (Figure 2G, Supplementary Figure S2). The results showed that circ_0008494 was abundantly located at the cytoplasm of HSCs in fibrous septa (Figure 2G). Moreover, an addition of 22 human HF tissues were collected and the qRT-PCR confirmed that circ_0008494 increased in HF tissues compared with matched normal liver tissues (Figure 2H). Furthermore, the expression of circ_0008494 was detected in the human HSC cell line LX-2. Fibro-genic factor TGF-β1 (15 ng/ml) was used to stimulate LX-2 cells. The qRT-PCR results showed that compared with the NC group, circ_0008494 significantly increased together with the HF indicators, α-SMA and Col1a1 (Figure 2I).

Together these results indicated that circ_0008494 might serve as a new regulator in the HF. Thus, circ_0008494 was selected as a promising candidate for further study.

### circ_0008494 knockdown inhibited the activation, proliferation, migration of HSCs and promoted their apoptosis

**FIGURE 3:**
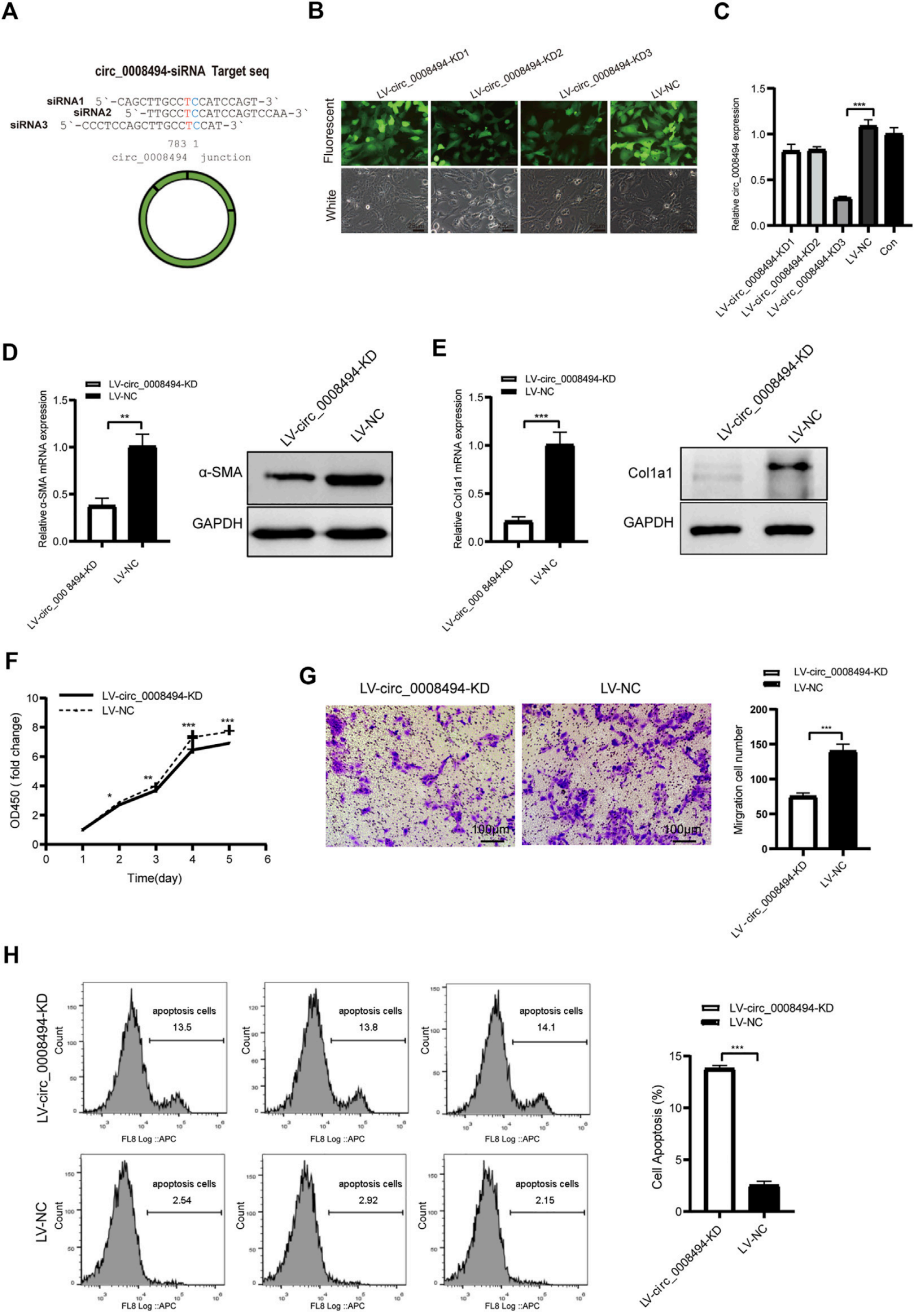
circ_0008494 knockdown inhibits the activation, proliferation, migration of HSCs and promoted their apoptosis **(A)** Three siRNAs targeting the junction sequence of circ_0008494 were designed **(B)** LX-2 cells emitted GFP fluorescence after circ_0008494-interfering viruses and NC virus infection (400×)**(C)** qRT-PCR showed LV-circ_0008494-KD3 exhibited the highest circ_0008494 reduction (0.30 ± 0.02) vs LV-NC **(D and E)** The mRNA and protein expression levels of a-SMA and Col1a1 in the LV-circ_0008494-KD and LV-NC groups **(F and G)** Effect of circ_0008494 knockdown on the proliferation and migration of LX-2 cells was detected by CCK8 and transwell assays. In CCK8 assay, the OD value at day1 was normalized to 1 and the data are expressed as fold **(H)** Apoptosis was detected by Annexin V-APC single staining combined with flow cytometry in LV-circ_0008494-KD and LV-NC groups. The RNA levels were normalized to total GAPDH. The protein levels were normalized to total GAPDH. Statistical analysis: Student *t*-tests. ^*^, ^**^, and ^***^ stand for *p* < 0.05, *p* < 0.01, and *p* < 0.001, respectively. Experiments were repeated independently three times.

Three siRNA interference targets were designed according to back-splice site sequence of circ_0008494 (Figure 3A; Table1). Virus packaging were completed and the construction framework of the lentiviral vectors were shown in Supplementary Table S5. LX-2 cells successfully infected with GFP-labeled lentiviral constructs LV-circ_0008494-KD1, LV-circ_0008494-KD2, LV-circ_0008494-KD3 and LV-NC were confirmed under a fluorescence microscope (Figure 3B). By qRT-PCR, the KD3 group exhibited the highest reduction in circRNA expression compared with the NC group (Figure 3C). However, the LV-circ_0008494-KD3 could not downregulate the linear mRNA of the ARID1A gene (Supplementary Figure S3). Next, LX-2 cells were infected with the circ_0008494-KD3 or NC lentiviral constructs, and clones resistant to puromycin were collected and cultured. A stable LV-circ_0008494-KD LX-2 cell line and LV-NC cell line were successfully established for subsequent experiments.

**TABLE 1:** Design of siRNA targeting circ_0008494.

siRNA	Target seq	Gc%
si1- circ_0008494	CAG​CTT​GCC​TCC​ATC​CAG​T	57.89%
si2- circ_0008494	TTG​CCT​CCA​TCC​AGT​CCA​A	52.63%
si3- circ_0008494	CCC​TCC​AGC​TTG​CCT​CCA​T	63.16%

The role of circ_0008494 in the development of HF was subsequently studied using LV-circ_0008494-KD LX-2 cell line or LV-NC cell line. The HF indicators, α-SMA and Col1a1, were detected by qRT-PCR and western blot assays. As is shown in Figures 3D, E, the mRNA and protein expression levels of α-SMA and Col1a1 in circ_0008494-knockdown LX-2 cells significantly decreased. The CCK8 test showed that the OD450 value (fold change) of the circ_0008494-KD group was lower than that of the NC group from day 2–5 (Figure 3F). The transwell assay showed that the migration ability was also reduced in the circ_0008494-KD group (Figure 3G). Additionally, Annexin V-APC single apoptosis test showed that the apoptotic proportion of the circ_0008494-KD group significantly increased than that of the NC group (Figure 3H). These results identified circ_0008494 as a new pro-fibrotic regulator of HF, suppressing circ_0008494 inhibited activation, proliferation, migration and promoted the apoptosis of HSCs.

### circ_0008494 acted as a sponge of miR-185-3p

**FIGURE 4:**
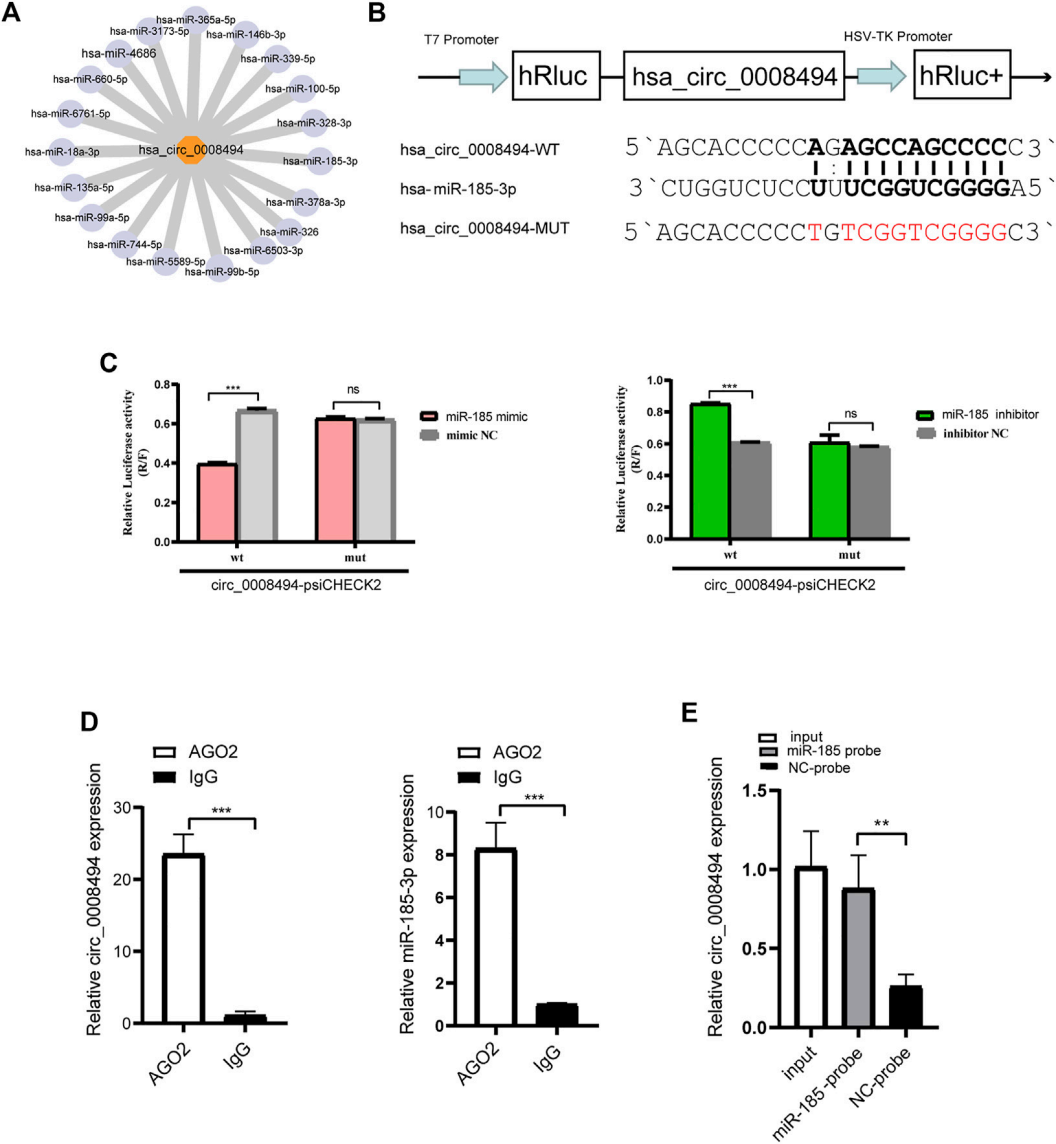
circ_0008494 acts as a sponge of miR-185-3p **(A)** miRanda algorithms predicted the binding miRNAs of circ_0008494 **(B)** psiCHECK luciferase reporter plasmid was used to construct circ_0008494-WT-psiCHECK and circ_0008494-MUT-psiCHECK **(C)** Dual-Luciferase reporter gene assay verified the binding relationship between circ_0008494 and miR-185-3p **(D)** Association of circ_0008494 and miR-185-3p with AGO2. Cellular lysates of LX-2 were used for the RIP assay with an AGO2 antibody (IgG as control). circ_0008494 and miR-185-3p levels were detected by qRT-PCR assay **(E)** Biotin-coupled miRNA capture assay was constructed to verify the binding between circ_0008494 and miR-185-3p using a biotinylated miR-185-3p probe and a miRNA NC probe. The RNA levels were normalized to total GAPDH. Statistical analysis: Student *t*-tests. ^**^ and ^***^ stand for *p* < 0.01 and *p* < 0.001, respectively. ns, nonsignificant. Experiments were repeated independently three times.

Given that exon-derived circRNAs usually function as miRNA sponge, miRNAs that can bind to circ_0008494 in our RNA-seq data were predicted by miRanda algorithms (Figure 4A). Nine predicted miRNAs showed significantly differential expression in HF tissues (FC ≥ 2 or≤0.5 and *p*-values ≤ 0.05), among which miR-185-3p was markedly downregulated and harbored an ideal binding target site for circ_0008494 (Supplementary Table S6).

A luciferase assay was further designed and performed (Figure 4B). As is shown in Figure 4C, the luciferase activity was significantly reduced in 293T cells co-transfected with miR-185-3p mimic and circ_0008494-WT plasmid compared with cells co-transfected with mimic NC and circ_0008494-WT plasmids. But there was no significant difference in the luciferase activity between circ_0008494-MUT plasmid and miR-185-3p mimic co-transfection group, as compared to circ_0008494-MUT plasmid and mimic NC co-transfection group. Besides, compared with cells co-transfected with circ_0008494-WT plasmid and miRNA inhibitor NC, the luciferase activity was significantly increased in 293T cells co-transfected with circ_0008494-WT plasmid and miR-185-3p inhibitor.

It was generally known that miRNA function as components of ribonucleoprotein (RNP) complexes or RNA-induced silencing complexes (RISCs), and AGO2 was one of the most important characterized components of RISCs. An RNA-RIP assay was performed to confirm whether circ_0008494 was associated with miRNA RNP. The results showed that both circ_0008494 and miR-185-3p were significantly enriched in AGO2 immunoprecipitated comparing to the control IgG group (Figure 4D). In addition, a biotin-coupled miRNA capture assay was performed using a biotin-coupled miR-185-3p probe, followed by qRT-PCR. It was observed a fourfold enrichment of circ_0008494 in the miR-185-3p captured fraction compared with the negative group (Figure 4E). These results suggested that miR-185-3p was a direct target of circ_0008494. Besides, both of circ_0008494 and miR-185-3p could interact with the AGO2 protein, indicating the possibility of circ_0008494 acting as a miRNA sponge for miR-185-3p.

### Suppressing circ_0008494 inhibited activation, proliferation, migration of HSCs and promoted their apoptosis through miR-185-3p

**FIGURE 5:**
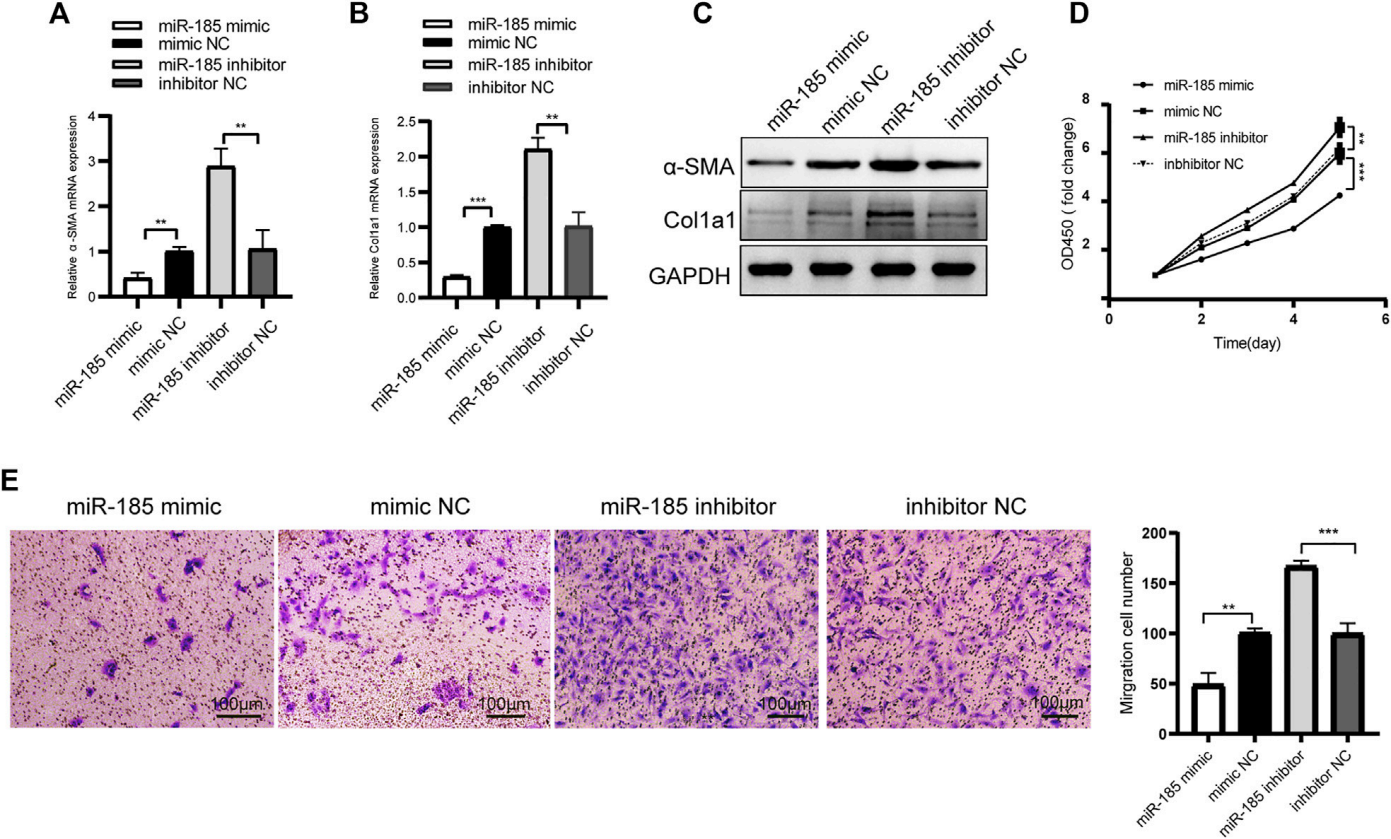
miR-185-3p inhibits HSCs activation, proliferation and migration**(A and B)** The mRNA expressions of α-SMA and Col1a1 were detected by qRT-PCR assay after miR-185-3p inhibitor/mimic transfection **(C)** Western blot assay showed the protein expression of α-SMA and Col1a1 after miR-185-3p mimic or mimic NC transfection**(D and E)** Proliferation and migration of LX-2 cells were detected by CCK8 and transwell assays after miR-185-3p inhibitor/mimic transfection. In CCK8 assay, the OD value at day1 was normalized to 1 and the data are expressed as fold. The RNA levels were normalized to total GAPDH. The protein levels were normalized to total GAPDH. Statistical analysis: Student *t*-tests. ^**^ and ^***^ stand for *p* < 0.01 and *p* < 0.001, respectively. Experiments were repeated independently three times.

We detected the expression level of miR-185-3p in TGF-β1 stimulated LX-2 cells. The qRT-PCR results showed that miR-185-3p expression in TGF-β1 stimulated LX-2 cells significantly decreased, comparing to the NC group (Supplementary Figure S4). Next, LX-2 cells were transfected with miR-185-3p mimic or inhibitor. The qRT-PCR results showed that the mRNA expression levels of α-SMA and Col1a1 significantly decreased in the miR-185-3p mimic transfected group but obviously increased in the miR-185-3p inhibitor transfected group compared with the miRNA NC groups (Figures 5A, B). Western blot assay demonstrated the same trend, which goes to show that miR-185-3p could well regulate the α-SMA and Col1a1 in protein level (Figure 5C). In addition, CCK8 and transwell assays further revealed that miR-185-3p mimic could decrease the proliferation and migration abilities of LX-2 cells, while miR-185-3p inhibitor had an opposite function (Figures 5D, E). Together, these results suggested that miR-185-3p is an anti-fibrotic regulator in HF.

**FIGURE 6:**
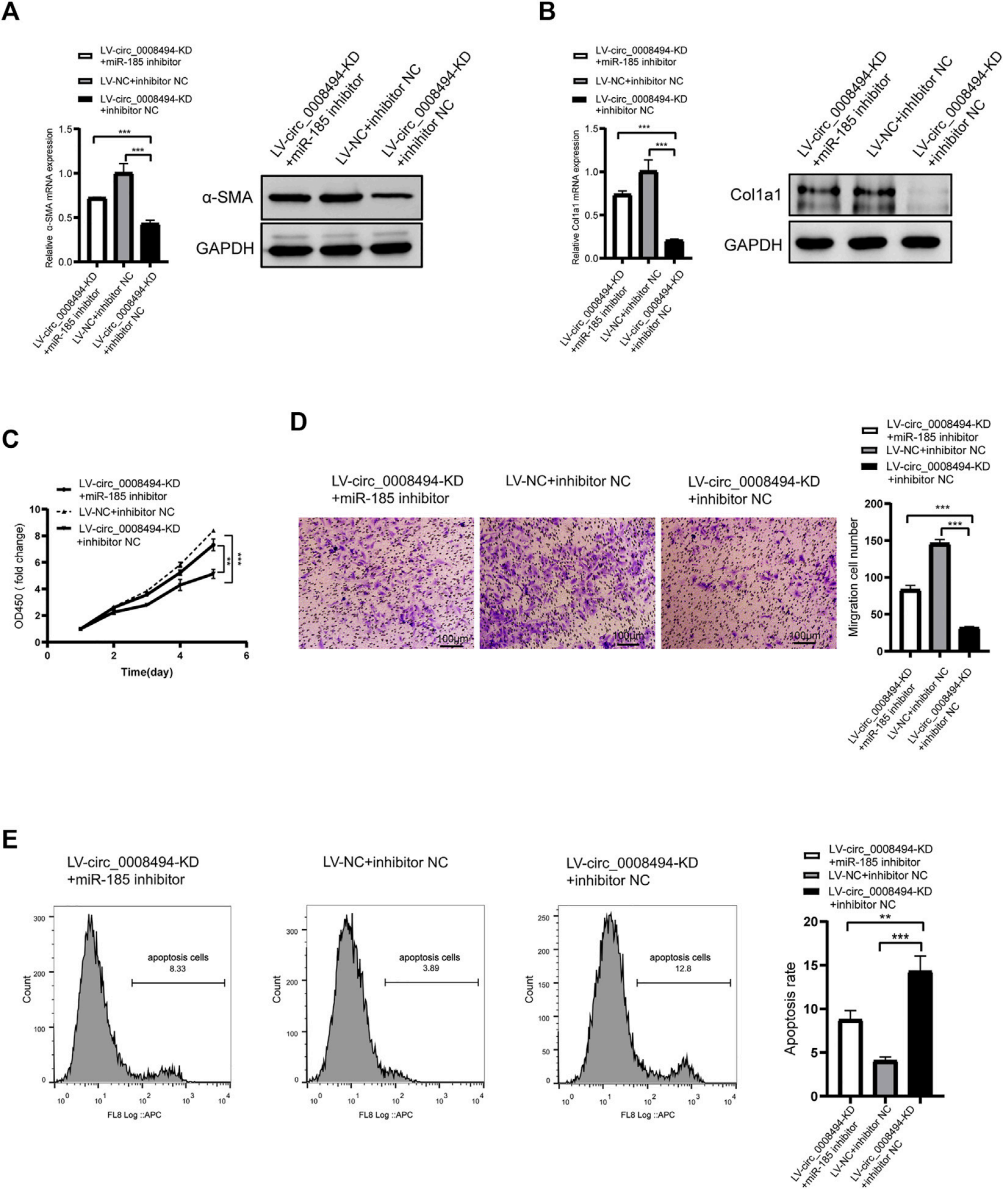
Suppressing circ_0008494 inhibits activation, proliferation, migration of HSCs and promotes their apoptosis through miR-185-3p **(A and B)** miR-185-3p inhibitor was transfected into stable LV-circ_0008494-KD LX-2 cells. miRNA inhibitor NC was transfected into stable circ_0008494-interfering cell line as a control or LX-2 NC cells as a normal control. 48 h after transfection, the mRNA and protein expression levels of a-SMA and Col1a1 in each group were detected qRT-PCR and western blot assays **(C and D)** After transfection as above, proliferation and migration of LX-2 cells in each group were detected by CCK8 and transwell assays. In CCK8 assay, the OD value at day1 was normalized to 1 and the data are expressed as fold **(E)** After transfection as above, apoptosis was detected by Annexin V-APC single staining combined with flow cytometry was performed in each group. The RNA levels were normalized to total GAPDH. The protein levels were normalized to total GAPDH. Statistical analysis: Student *t*-tests. ^**^ and ^***^ stand for *p* < 0.01and *p* < 0.001, respectively. Experiments were repeated independently three times.

To further confirm whether the regulatory effect of circ_0008494 on HSCs is dependent on its interaction with miR-185-3p, a rescue assay was designed. miR-185-3p inhibitor was transfected into stable circ_0008494-interfering cell line, while miRNA inhibitor NC was transfected into stable circ_0008494-interfering cell line as a control or into LX-2 NC cells as a normal control. As is shown in Figures 6A, B, miR-185-3p inhibitor partially restored the reduced expression of α-SMA and Col1a1 after it was transfected into the circ_0008494-knockdown LX-2 cells, comparing with the inhibitor NC transfection stable circ_0008494-interfering group. Figures 6C–E showed miR-185-3p inhibitor partially promoted the proliferation and migration of HSCs and ameliorated their apoptosis after it was transfected into the circ_0008494-knockdown group when compared with the miRNA inhibitor NC transfected stable circ_0008494-knockdown LX-2 cells. These results suggest that circ_0008494 can regulate the activation and biological function of HSCs by sponging miR-185-3p.

### Suppressing circ_0008494 inhibited HSCs activation through the miR-185-3p/Col1a1 axis

**FIGURE 7:**
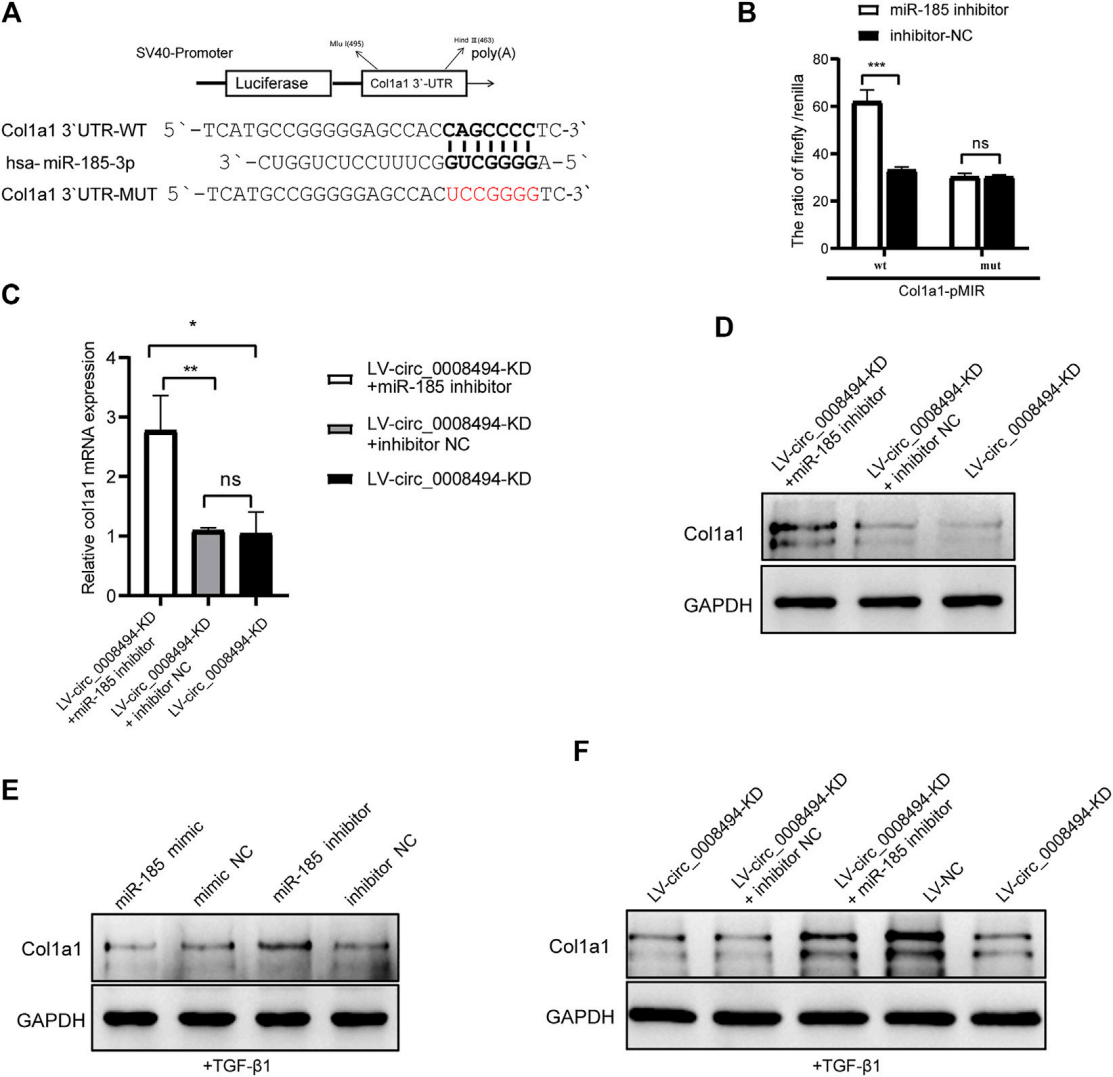
Suppressing circ_0008494 inhibits HSCs activation by regulating the miR-185-3p/Col1a1 axis **(A)**The pMIR-REPORT luciferase reporter plasmid was used to construct Col1a1 3′UTR-WT-pMIR and Col1a1 3′UTR-MUT-pMIR. TargetScan website predicted seven ribonucleotides of has-miR-185-3p were complementary to the 642–648 sites of Col1a1 3′-UTR **(B)** Dual-luciferase reporter gene assay verified the binding relationship between miR-185-3p and Col1a1**(C and D)** miR-185-3p inhibitor or inhibitor NC was transfected into stable LV-circ_0008494-KD LX-2 cells, and mRNA and protein levels of Col1a1 was detected by qRT-PCR and western blot assays **(E)** After TGF-β1 stimulation, miR-185-3p inhibitor/mimic were transfected into LX-2 cells, and Col1a1 protein expression level were detected by western blot assay **(F)** After TGF-β1 stimulation of stable LV-circ_0008494-KD cells or LV-NC cells, Col1a1 protein expression was detected by western blot assays. Moreover, miR-185-3p inhibitor or inhibitor NC was transfected into stable LV-circ_0008494-KD LX-2 cells after TGF-β1 stimulation, and Col1a1 protein expression was detected. The RNA levels were normalized to total GAPDH. The protein levels were normalized to total GAPDH. Statistical analysis: Student *t*-tests. ^*^, ^**^, and ^***^ stand for *p* < 0.05, *p* < 0.01 and *p* < 0.001, respectively. ns, nonsignificant. Experiments were repeated independently three times.

Considering that HSCs have a particularly complex regulatory network, candidate targets of miR-185-3p were predicted by using literature search and TargetScan online website (Liao and Lu, 2011; Li G et al., 2015; Li et al., 2017; Lu et al., 2018; Ma et al., 2019; Gao et al., 2020; Ou et al., 2020) (Supplementary Table S7). The predicted candidates FSCN1, MLCK, GREM1, c-Myc, WNT2B, E2F1, BRD4, Col1a1, FGF5, TIMP2 and TGFBR2 were then detected by qRT-PCR in LX-2 cells transfected with miR-185-3p mimic and mimic NC. As is presented in Supplementary Figure S5, Col1a1, FGF5 and BRD4 were significantly downregulated after miR-185-3p mimic transfection. Among the three genes, Col1a1 showed the most obvious reduction. It was nearly downregulated to 30%, compared to FGF5 (57%) and BRD4 (67%) by qRT-qPCR detection. Besides, the three genes were up-regulated in TGF-β1 stimulated LX-2 cells, indicating that they might play a role in HSCs activation (Supplementary Figure S6). Western blot analysis confirmed that miR-185-3p mimic downregulated the protein levels of Col1a1, FGF5 and BRD4 but miR-185-3p inhibitor promoted their expression (Supplementary Figure S7). In view of the deposition of collagen I playing a key role in HF, Col1a1 was selected for further research. Using the TargetScan website, we found that seven ribonucleotides of hsa-miR-185-3p were complementary to the 642–648 site of the Col1a1 3′UTR. The direct binding between miR-185-3p and Col1a1 was verified by dual-luciferase reporter assay (Figure 7A). Compared with cells co-transfected with Col1a1-WT plasmid and miRNA inhibitor NC, the luciferase activity was significantly increased in 293T cells co-transfected with Col1a1-WT plasmid and miR-185-3p inhibitor. But there was no significant difference in the luciferase activity between Col1a1-MUTANT plasmid and miRNA inhibitor NC co-transfection group, as compared to Col1a1-MUTANT plasmid and miR-185-3p inhibitor co-transfection group (Figure 7B). The result confirmed that Col1a1 was a bona fide target gene of miR-185-3p.

Col1a1 was a direct target gene of miR-185-3p and Col1a1 itself is also a key indicator of HSCs activation and HF. To further confirm whether the regulatory effect of circ_0008494 on Col1a1 was dependent on its interaction with miR-185-3p, a rescue assay was designed. miR-185-3p inhibitor or inhibitor NC was transfected into the stable circ_0008494-interfering cell line. Quantitative real-time PCR and western blot assays were used to detect the expression of Col1a1. As is shown in Figures 7C, D, miR-185-3p inhibitor significantly reversed the decreased expression of Col1a1 both in mRNA and protein levels in stable circ_0008494-interfering LX-2 cells, whereas inhibitor NC had no effect. Besides, TGF-β1 was used to stimulate the LX-2 cells, mimicking HSCs activation *in vivo*. After TGF-β1 stimulation, miR-185-3p mimic or miR-185-3p inhibitor was transfected into LX-2 cells, Col1a1 protein decreased in miR-185-3p mimic group but increased in miR-185-3p inhibitor group (Figure 7E). Besides, after TGF-β1 stimulation was run in stable circ_0008494-interfering cells or LV-NC cells, Col1a1 protein increased significantly in LV-NC cells, but showed less remarkable elevation in LV-circ_0008494-KD cells (Figure 7F). Importantly, miR-185-3p inhibitor or inhibitor NC was transfected into the TGF-β1 stimulated LV-circ_0008494-KD cells. miR-185-3p inhibitor significantly reversed the decreased protein expression of Col1a1 during this stimulation process, whereas inhibitor NC did not have the same effect (Figure 7F). These results further indicated that circ_0008494 and miR-185-3p could regulate Col1a1 during HSCs activation. Suppressing circ_0008494 attenuated its competitive inhibition to miR-185-3p, thereby attenuating the expression of Col1a1.

## Discussion

Hepatic fibrosis-associated morbidity is progressively increasing worldwide, but successful antifibrotic treatment is still lacking. In addition, uncontrolled HF may eventually progress into irreversible liver injury. HSCs are central players in the pathogenesis of HF. Following liver damage, HSCs are activated to produce smooth muscle actin protein and secrete large amounts of ECM (Friedman, 2008a; Friedman, 2008b; Breitkopf-Heinlein K et al., 2017). Collagens are the most important ECM components in the liver. Suffering from liver injury, the normal matrix of the Disse space is disrupted and replaced by fibrillar collagens, including primarily collagen I and collagen III, especially collagen I. The deposition of type I and type III collagens not only continuously activates HSCs but also keeps them in an active state, thereby causing hepatocyte dysfunction (Hernandez-Gea and Friedman, 2011). Thus, inhibition of liver collagen production is critical to the prevention and treatment of HF.

At present, the role of circRNAs as a novel type of competitive endogenous RNA is generally accepted. They contain MREs and can competitively bind to miRNAs, thereby augmenting the expression the downstream target genes of miRNAs. In the past 2 years, circRNAs research in the field of HF has received some progress. Some circRNAs, such as circFBXW4, cMTO1 and circ_0004018, have been demonstrated to participate in the regulation of the proliferation and activation of HSCs by sponging miR-18b-3p, miR-181-5p or miR-660-3p and modulating their target genes, including FBXW7, PTEN and TEP1 (Jin H. et al., 2020; Chen et al., 2020; Li et al., 2020).

In our research, we were initially concerned about circ_0008494, which is derived from three exons of the ARID1A gene and abundantly over-expressed in RNA-seq data. Bioinformatics analysis revealed multiple HF related genes, particularly mounts of collagen genes, were located in its circRNA-miRNA-mRNA interaction network. Given that the function of circRNA mainly depends on its downstream target genes, circ_0008494 was selected as a promising HF regulator for further research. Sanger sequencing, ActD and RNase R treatment assays confirmed its circular structure. Importantly, FISH assay demonstrated that circ_0008494 was primarily located in the cytoplasm of HSCs of the fibrosis region and circ_0008494 was both up-regulated in additional 22 HF samples and TGF-β1 activated LX-2 cells. Through cell functional experiments, we further demonstrated that the activation, proliferation and migration abilities of HSCs were markedly attenuated whereas the apoptotic proportion was significantly increased after circ_0008494 knockdown. These results suggested that circ_0008494 served as a novel pro-fibrotic regulator in the HF. We assessed the circ_0008494 ceRNA network and miR-185-3p harbored an ideal target site for circ_0008494. Using dual-luciferase reporter, AGO-RIP and biotin-coupled miRNA capture assays, we confirmed that miR-185-3p was a direct target of circ_0008494.

miR-185-3p has been demonstrated to act as a tumor suppressor in various malignancies, such as nasopharyngeal carcinoma, breast cancer and colorectal cancer (Li G et al., 2015; Lu et al., 2018; Ou et al., 2020). In our study, we discovered that miR-185-3p was downregulated in TGF-β1-stimulated HSCs and inhibited the activation of HSCs markedly. Rescue assay demonstrated that suppressing circ_0008494 inhibited the activation, proliferation and migration of HSCs, and promoted their apoptosis through miR-185-3p, at least in part. Through literature search and bioinformatics analysis, we predicted potential targets of miR-185-3p and through qRT-PCR and western blot assays. We found Col1a1, FGF5 and BRD4 were significantly downregulated at both the mRNA and protein levels in LX-2 cells after miR-185-3p mimic transfection. Fibroblast growth factors (FGFs) are heparin-binding polypeptides which function in numerous cellular developmental and metabolic processes. It was reported that FGF5 played significant roles in diet-induced steatohepatitis and fibrosis (Hanaka et al., 2014; Nakashima et al., 2016). BRD4 is a member of the BET family proteins (Cai et al., 2018). Huang et al. reported that microRNA-29a could mitigate HF in mice and inhibit HSCs activation by regulating BRD4 (Huang et al., 2019). However, the deposition of collagen I plays a key role in HF, and among these three genes, Col1a1 showed the most obvious reduction after miR-185-3p mimic transfection in qPCR assay. The luciferase assay further verified that Col1a1 was a direct target gene of miR-185-3p, and rescue assays confirmed that the regulatory effect of circ_0008494 on Col1a1 was dependent on its interaction with miR-185-3p. miR-185-3p inhibitor significantly reversed the lowered levels of Col1a1 in stable circ_0008494-interfering LX-2 cells. To further verify the regulatory effect of circ_0008494/miR-185-3p on Col1a1 in the activation of HSCs, recombinant human TGF-β1 was used to activate LX-2 cells. TGF-β1 is one of the most potent cytokines which can accelerate HF through promoting transcriptions of type I and type III collagen and promoting HSC-to-myofibroblast transdifferentiation (Hellerbrand et al., 1999; Xu et al., 2016). TGF-β1 was widely recognized to stimulate LX-2 cells for further activation, mimicking the HSCs activation process *in vivo* (Liu et al., 2019; Wang et al., 2019). After TGF-β1 was added, we found the expression of Col1a1 was obviously inhibited in the TGF-β1 stimulated LV-circ_0008494-KD cells comparing with the TGF-β1-treated LV-NC cells and miR-185-3p inhibitor significantly reversed the decreased protein expression of Col1a1 during this stimulation process. Hence, the effective role of circ_0008494/miR-185-3p/Col1a1 axis in HSCs activation was well demonstrated.

In conclusion, circ_0008494 was identified as a new pro-fibrotic regulator of HF. Our study demonstrates that the circ_0008494/miR-185-3p axis not only regulates the proliferation, migration and apoptosis of HSCs, but in particular, it has the ability to regulate the activation of HSCs by directly targeting Col1a1, which is the key indicator of HSCs activation and HF itself. Knocking down circ_0008494 remarkably ameliorated the expression of Col1a1 by freeing of miR-185-3p. Therefore, the role of circ_0008494/miR-185-3p/Col1a1 axis in HSC activation and HF deserves serious attention, as it may serve as a promising and effective target for the treatment of HF.

However, some limitations also existed in this study. Considering that the sequences of circRNAs in humans and mice are inconsistent, we did not conduct animal experiments in this study. And whether circ_0008494 knockdown in HSCs has an effect on other intrahepatic cells (such as hepatocyte and Kupffer cell) needs further attention. Interestingly, some other potential targets of circ_0008494 were also noted during our study, such as FGF5 and BRD4, which reflects the complexity of the circRNA-miRNA-ceRNA network in HSCs. Our results provide new insight into the ceRNAs network in HF and make a beneficial contribution to the identification of effective HF therapeutic targets.

## Data Availability

The datasets presented in this study can be found in online repositories. The names of the repository/repositories and accession number(s) can be found below: https://www.ncbi.nlm.nih.gov/geo/query/acc.cgi?acc=GSE191247.
