# Supplementary material for: Suppressing circ_0008494 inhibits HSCs activation by regulating the miR-185-3p/Col1a1 axis
Source: Front Pharmacol. 2022 Nov 17;13:1050093. doi: 10.3389/fphar.2022.1050093 (PMC9713816; doi:10.3389/fphar.2022.1050093)
Supplement: Supplementary file 2 [file DataSheet3.docx]

Supplementary Material

# Supplementary Figures and Tables

## Supplementary Figures


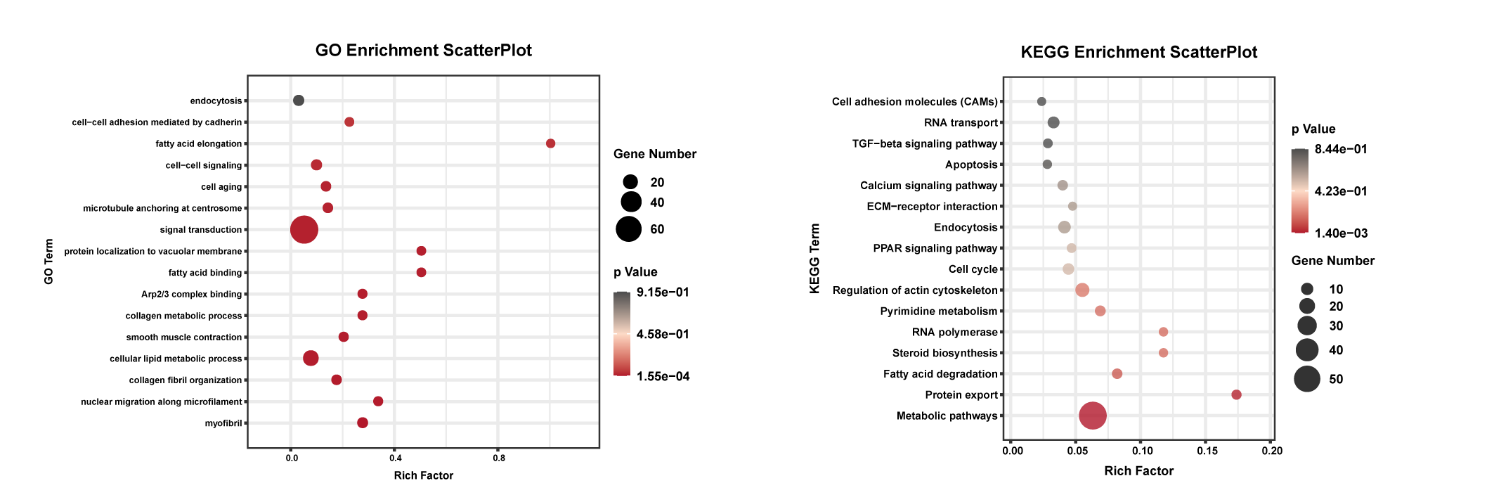


**FIGURE1**| GO and KEGG enrichment scatterplots of the parental genes of the downregulated circRNAs.


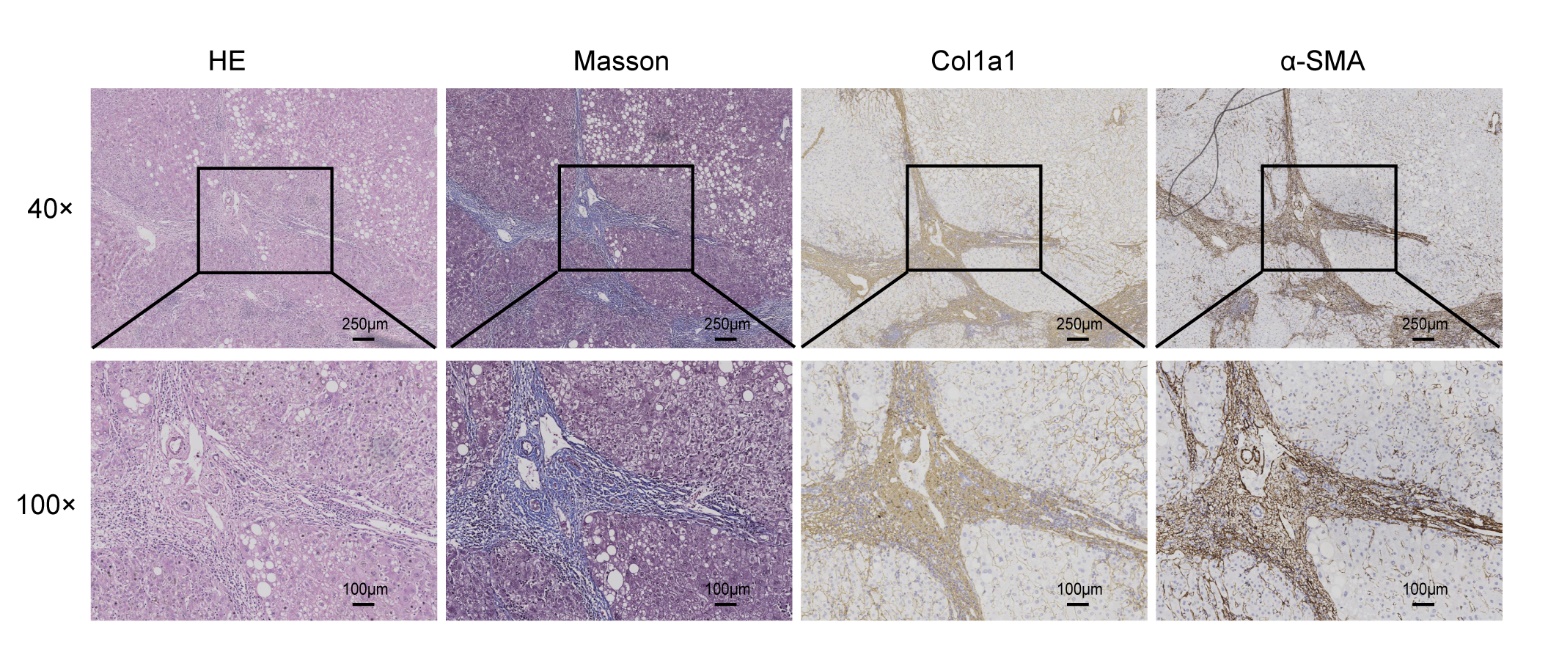
 **FIGURE2**| HE、Masson and immunohistochemical (IHC) staining of human HF tissue.

**
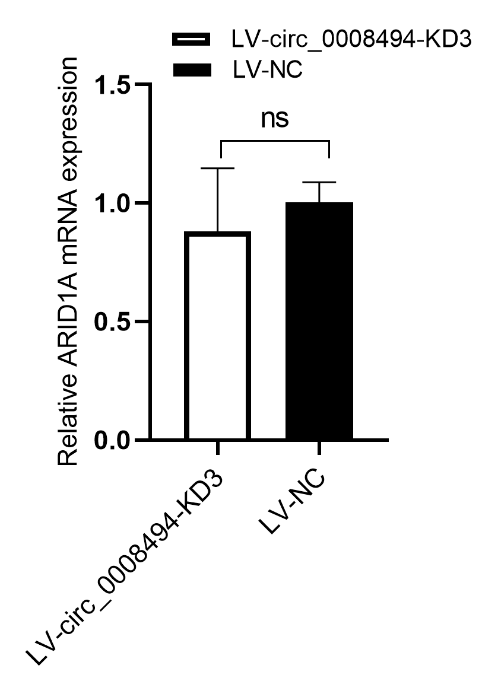
**

**FIGURE3**| **qRT**-PCR showed LV-circ_0008494-KD3 could not downregulate the linear mRNA of the ARID1A gene. ns, nonsignificant.


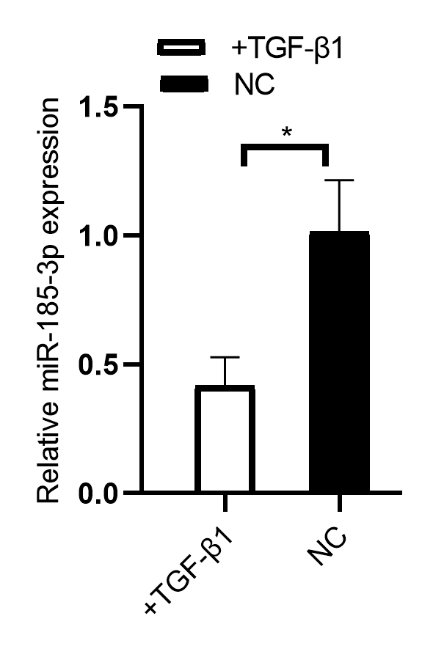


**FIGURE4**| **Expression** of miR-185-3p in TGF-β1 activated LX-2 cells by qRT-PCR assay. The RNA levels were normalized to U6. ^*^ stands for p<0.0.5.


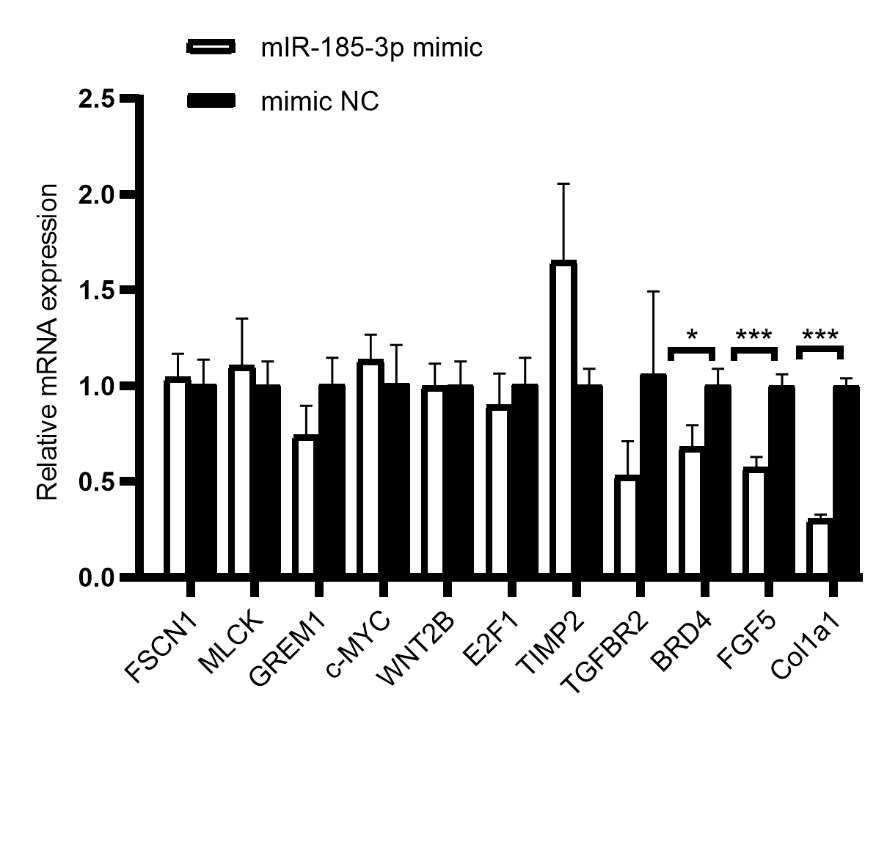


**FIGURE5**| The expression of predicted target genes of miR-185-3p was detected. qRT-PCR assay showed the mRNA expression of predicted target genes of miR-185-3p after miR-185-3p mimic or mimic NC transfection. The RNA levels were normalized to total GAPDH. ^*^and ^***^ stand for p<0.05 and p<0.001, respectively.

**
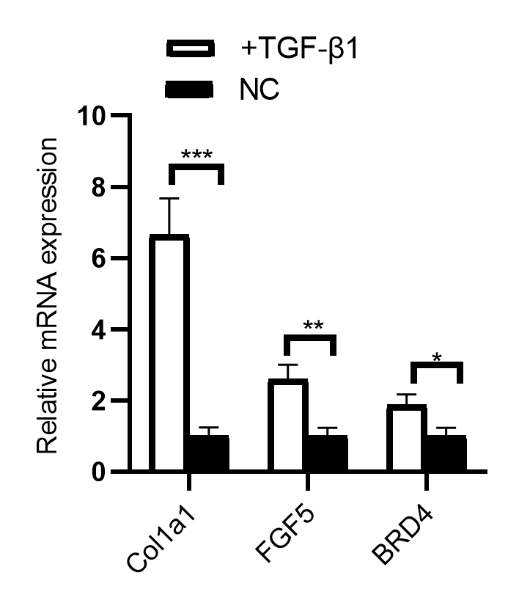
**

**FIGURE6**| Expression of Col1a1, FGF5 and BRD4 in TGF-β1 activated LX-2 cells by qRT-PCR assay. The RNA levels were normalized to total GAPDH. ^*^, ^**^and ^***^ stand for p<0.05, p<0.01 and p<0.001, respectively.


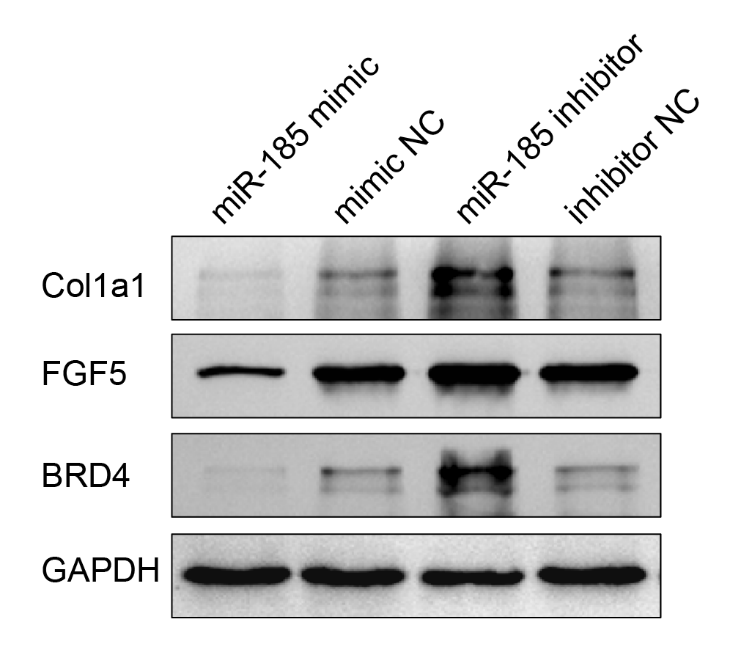


**FIGURE7**| **The** protein expression of Col1a1, FGF5 and BRD4 was detected by western blot assay after miR-185-3p inhibitor/mimic transfection. The protein levels were normalized to total GAPDH.

## Supplementary Tables

Table 1 Clinical Data of HF patients for RNA-seq

| Gender | Age | Fibrosis stage | Etiology | HBsAg HBsAb  HBeAg HBeAb  HBcAb | ALT  (IU/L) | AST  (IU/L) |  | HBV DNA  (IU/ml) |
| --- | --- | --- | --- | --- | --- | --- | --- | --- |
| Female | 56 | F1 | HBV | - +  - -  + | 17 | 34 |  | <500 |
| Male | 59 | F1 | HBV | + -  - +  + | 247 | 174 |  | <500 |
| Male | 65 | F2 | HBV | - -  - +  + | 51 | 44 |  | <500 |
| Male | 67 | F2 | HBV | + -  - +  + | 66 | 44 |  | 8.5e+02 |
| Male | 61 | F3 | HBV | + -  + -  + | 116 | 36 |  | 2.7e+05 |
| Male | 62 | F4 | HBV | + -  - ±  + | 559 | 997 |  | <500 |

Note：ALT（Alanine aminotransferase）reference range0~50IU/L

AST（aspartate aminotransferase）reference range0~40IU/L

Table2 RNA QC Evaluation of the 6 fibrosis samples for RNA-seq

| Sample  ID | Conc. (µg/µL) | O.D.  260/280 | O.D.  260/230 | Amount  (µg) | rRNA  28S/18S | RIN | QC  Evaluation |
| --- | --- | --- | --- | --- | --- | --- | --- |
| C3 | 0.24 | 2.12 | 1.56 | 11.88 | 1.8 | 7.8 | A |
| C9 | 0.84 | 2.18 | 2.08 | 37.71 | 1.30 | 8.00 | A |
| C10 | 1.46 | 2.15 | 2.24 | 65.54 | 1.40 | 8.20 | A |
| C11 | 0.81 | 2.17 | 1.86 | 36.41 | 1.30 | 7.30 | A |
| C12 | 0.67 | 2.14 | 2.23 | 30.09 | 1.40 | 8.40 | A |
| C13 | 0.76 | 2.18 | 2.28 | 34.34 | 1.20 | 7.10 | A |

Note：All RNA samples for RNA-seq were of high-quality with a 28S/18S ratio ≥ 1.0 and RIN ≥ 7.0 after an RNA quality assessment.

Table3 Primer information for mRNA qRT-PCR

| **Species** | **Gene** | **Primer sequence** （**5'-3'**） |
| --- | --- | --- |
| Homo | α-SMA | F: TATGCCTCTGGACGCACAACT |
|  |  | R: GCTCAGCAGTAGTAACGAAGGAA |
| Homo | Col1a1 | F: ACGAAGACATCCCACCAATCA |
|  |  | R: CAGATCACGTCATCGCACAAC |
| Homo | GAPDH | F: GAACGGGAAGCTCACTGG  R: GCCTGCTTCACCACCTTCT |
| Homo | FSCN1 | F: ACCTGTCTGCCAATCAGGAC  R: AGTACTTGCCCGTGTGGGTA |
| Homo | BRD4 | F: CAACAAGCCTGGAGATGACA |
|  |  | R: CGGTTTCTTCTGTGGGTAGC |
| Homo | MLCK | F: ATGCTGTCCATGAGGAGGAC |
|  |  | R: AAGCTGCTTCGCAAAACTTC |
| Homo | E2F1 | F: CCAGGAGGTCACTTCTGAGG |
|  |  | R: ATCTGTGGTGAGGGATGAGG |
| Homo | WNT2B | F: CGGTTGTGAAATCATGTGCT |
|  |  | R: CACAGCACCAGTGGAATTTG |
| Homo | c-Myc | F: TCAAGAGGCGAACACACAAC |
|  |  | R: GGCCTTTTCATTGTTTTCCA |
| Homo | GREM1 | F: ATCAACCGCTTCTGTTACGG |
|  |  | R: AATTTCTTGGGCTTGCAGAA |
| Homo | TGFBR2 | F: CAGCCCAGTGTGTGGCAGAA |
|  |  | R: AGAGGGGCAGCCTCTTTGGA |
| Homo | FGF5 | F: GCAGACAGAGCAGCAGTAGC |
|  |  | R: GGAAACTGCTCTGCTCCAAG |
| Homo | TIMP2 | F: AAGCGGTCAGTGAGAAGG |
|  |  | R: TCTCAGGCCCTTTGAACATC |
| Homo | ARID1A | F: ACCACAAGCACCCAGAACGG  R: CCCGGAGCATCTCCAAGCAG |

Table4 Sequencing information and primer design of circ_0008494

| Category | Information |
| --- | --- |
| circRNA | hsa_circ_0008494 |
| Gene name | ARID1A |
| Chromosome | chr1:27056141-27059283 |
| Lengh  Up/down  Significant  Primer sequence | 783bp  Up  Yes  F: AGATTCATTTGGGTCTCAGGC  R: ATTGGACTGGATGGAGGCA |

Table5 Framework for circ_0008494-interfering lentiviral vector construction

| Number | 5’ | STEM | Loop | STEM | 3’ |
| --- | --- | --- | --- | --- | --- |
| circRNA-KD1-a | Ccgg | CAGCTTGCCTCCATCCAGT | CTCGAG | ACTGGATGGAGGCAAGCTG | TTTTTg |
| circRNA- KD1-b | aattcaaaaa | CAGCTTGCCTCCATCCAGT | CTCGAG | ACTGGATGGAGGCAAGCTG |  |
| circRNA- KD2-a | Ccgg | TTGCCTCCATCCAGTCCAA | CTCGAG | TTGGACTGGATGGAGGCAA | TTTTTg |
| circRNA- KD2-b | aattcaaaaa | TTGCCTCCATCCAGTCCAA | CTCGAG | TTGGACTGGATGGAGGCAA |  |
| circRNA- KD3-a | Ccgg | CCCTCCAGCTTGCCTCCAT | CTCGAG | ATGGAGGCAAGCTGGAGGG | TTTTTg |
| circRNA- KD3-b | aattcaaaaa | CCCTCCAGCTTGCCTCCAT | CTCGAG | ATGGAGGCAAGCTGGAGGG |  |

Table6 Sequencing information of the 9 predicted miRNAs

| miRNA | miRNA seq | | Up/down | Log2FC | pVal(t) | Context score | Site Type | Significant |
| --- | --- | --- | --- | --- | --- | --- | --- | --- |
| miR-365a-5p  miR-185-3p  miR-744-5p  miR-99b-5p  miR-99a-5p  miR-100-5p  miR-146b-3p  miR-328-3p  miR-339-5p | | agggacttttgggggcagatgtg  aggggctggctttcctctggt  tgcggggctagggctaacagca  cacccgtagaaccgaccttgcg  aacccgtagatccgatcttgt  aacccgtagatccgaacttgtg  gccctgtggactcagttctggt  ctggccctctctgcccttccgt  tccctgtcctccaggagctca | down  down  down  up  up  up  up  up  up | -3.78  -2.50  -0.98  2.18  3.09  3.96  4.56  4.76  6.86 | 0.0229  0.0325  0.0212  0.0100  0.0132  0.0202  0.0009  0.0011  0.0143 | 42  95  15  32  48  36  37  73  12 | 1a  8mer  1a  m8  m8  m8  1a  m8  1a | Yes  Yes  Yes  Yes  Yes  Yes  Yes  Yes  Yes |

Table7 Predicted targets of miR-185-3p

| Predicted targets | | Reported pathways | TargetScan  (site type,3`UTR) | |
| --- | --- | --- | --- | --- |
| FSCN1  MLCK  GREM1  c-Myc  WNT2B  E2F1  BRD4  Col1a1  FGF5  TIMP2  TGFBR2 | LINC00152/miR-185-3p/FSCN1^(Ou et al., 2020)^  lncRNA /miR-185-3p/MLCK^(Ma et al., 2019)^  miR-185-3p/GREM1^(Li et al., 2017)^  miR-185-3P/c-Myc^(Liao and Lu, 2011)^  miR-185-3p/WNT2B^(Li et al., 2015)^  LINC00511/miR-185-3p/E2F1/Nanog ^(Lu et al., 2018)^  circ_0074027/miR-185-3p/BRD4 /MADD ^(Gao et al., 2020)^ | | | 7mer-m8,642~648  7mer-m8,130~136  7mer-A1,596~602  7mer-A1,712~718 |

Gao, P., Wang, Z., Hu, Z., Jiao, X., and Yao, Y. (2020). Circular RNA circ_0074027 indicates a poor prognosis for NSCLC patients and modulates cell proliferation, apoptosis, and invasion via miR-185-3p mediated BRD4/MADD activation. *J Cell Biochem* 121**,** 2632-2642. doi: 10.1002/jcb.29484

Li, G., Wang, Y., Liu, Y., Su, Z., and Qiu, Y. (2015). miR-185-3p regulates nasopharyngeal carcinoma radioresistance by targeting WNT2B in vitro. *Cancer Science* 105**,** 1560-1568. doi:[10.1111/cas.12555](https://doi.org/10.1111/cas.12555)

Li, J., Liu, H., Zou, L., Ke, J., Zhang, Y., Zhu, Y., Yang, Y., Gong, Y., Tian, J., Zou, D., Peng, X., Gong, J., Zhong, R., Huang, K., Chang, J., and Miao, X. (2017). A functional variant in GREM1 confers risk for colorectal cancer by disrupting a hsa-miR-185-3p binding site. *Oncotarget* 8**,** 61318-61326. doi:10.18632/oncotarget.18095

Liao, J.M., and Lu, H. (2011). Autoregulatory suppression of c-Myc by miR-185-3p. *Journal of Biological Chemistry* 286**,** 33901-33909. doi:10.1074/jbc.M111.262030

Lu, G., Li, Y., Ma, Y., Lu, J., Chen, Y., Jiang, Q., Qin, Q., Zhao, L., Huang, Q., Luo, Z., Huang, S., and Wei, Z. (2018). Long noncoding RNA LINC00511 contributes to breast cancer tumourigenesis and stemness by inducing the miR-185-3p/E2F1/Nanog axis. *J Exp Clin Cancer Res* 37**,** 289. doi:10.1186/s13046-018-0945-6

Ma, D., Cao, Y., Wang, Z., He, J., Chen, H., Xiong, H., Ren, L., Shen, C., Zhang, X., Yan, Y., Yan, T., Guo, F., Xuan, B., Cui, Z., Ye, G., Fang, J., Chen, H., and Hong, J. (2019). CCAT1 lncRNA Promotes Inflammatory Bowel Disease Malignancy by Destroying Intestinal Barrier via Downregulating miR-185-3p. *Inflammatory bowel diseases* 25**,** 862-874. doi:10.1093/ibd/izy381

Ou, C., Sun, Z., He, X., Li, X., and Ma, J. (2020). Targeting YAP1/LINC00152/FSCN1 Signaling Axis Prevents the Progression of Colorectal Cancer. *Advanced ence* 7**,** 1901380. doi: 10.1002/advs.201901380
