# Supplementary material for: Suppressing circ_0008494 inhibits HSCs activation by regulating the miR-185-3p/Col1a1 axis
Source: Front Pharmacol. 2022 Nov 17;13:1050093. doi: 10.3389/fphar.2022.1050093 (PMC9713816; doi:10.3389/fphar.2022.1050093)
Supplement: Supplementary file 3 [file DataSheet1.ZIP › raw data1/Bioinformatics/Figure3A circ_0008494 and siRNA design.docx]

http://www.circbank.cn/infoCirc.html?id=hsa_circARID1A_002


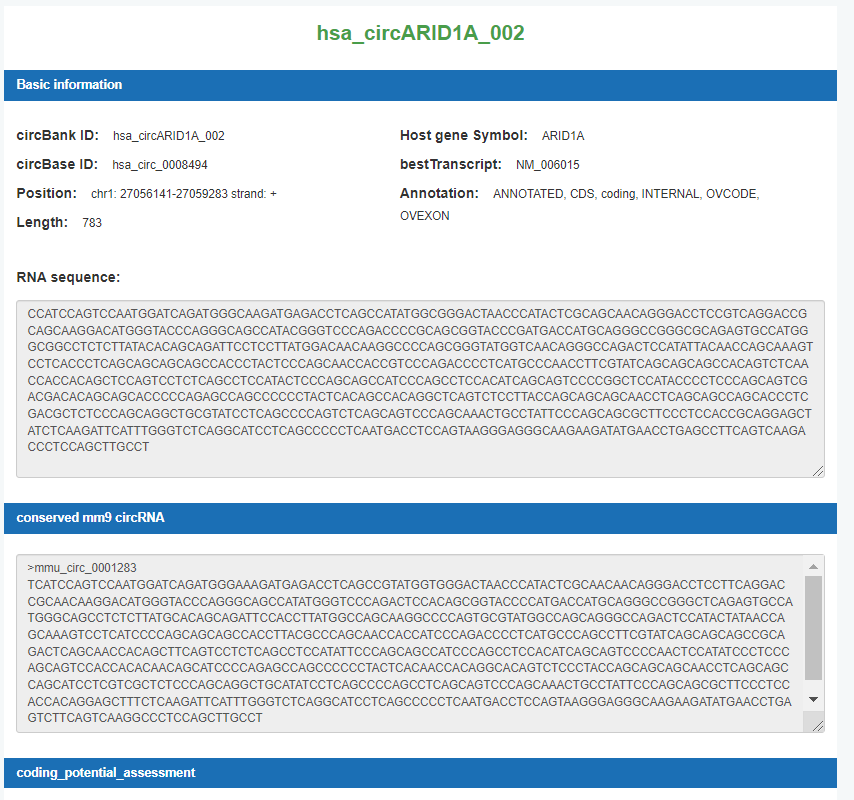


siRNA design for target circ_0008494

| siRNA | | Target Seq | | GC% |
| --- | --- | --- | --- | --- |
| si1- circ_0008494 | CAGCTTGCCTCCATCCAGT | | 57.89% | |
| si2- circ_0008494 | TTGCCTCCATCCAGTCCAA | | 52.63% | |
| si3- circ_0008494 | CCCTCCAGCTTGCCTCCAT | | 63.16% | |
