# Supplementary material for: Suppressing circ_0008494 inhibits HSCs activation by regulating the miR-185-3p/Col1a1 axis
Source: Front Pharmacol. 2022 Nov 17;13:1050093. doi: 10.3389/fphar.2022.1050093 (PMC9713816; doi:10.3389/fphar.2022.1050093)
Supplement: Supplementary file 3 [file DataSheet1.ZIP › raw data1/Figure4C 7B Luciferase Report/Luciferase reporter assay Plasmid sequence.docx]

**1 The dual luciferase reporter assay for circ_0008494 and miR-185-3p**


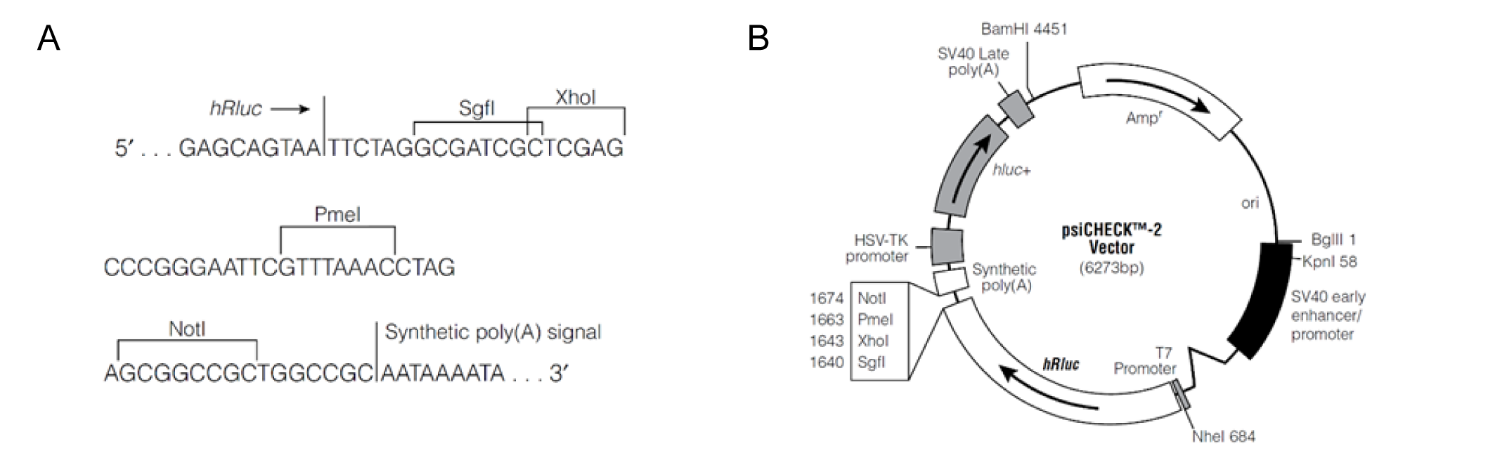
**1)** Schematic diagram of the psiCHECK plasmid

The psiCHECK luciferase reporter plasmid was used to construct luciferase reporter plasmids of circ_0008494.A plasmid multi-cloning sites of luciferase reporter plasmid. B Schematic diagram of the psiCHECK plasmid

2)plasmid sequences

>psi-hsa_circ_0008494-150bp-wt-psiCHECK2

CACATCAGCAGTCCCCGGCTCCATACCCCTCCCAGCAGTCGACGACACAGCAGCACCCCCAGAGCCAGCCCCCCTACTCACAGCCACAGGCTCAGTCTCCTTACCAGCAGCAGCAACCTCAGCAGCCAGCACCCTCGACGCTCTCCCAGC

>psi-hsa_circ_0008494-150bp-mut-psiCHECK2

CACATCAGCAGTCCCCGGCTCCATACCCCTCCCAGCAGTCGACGACACAGCAGCACCCCCTGTCGGTCGGGGCCTACTCACAGCCACAGGCTCAGTCTCCTTACCAGCAGCAGCAACCTCAGCAGCCAGCACCCTCGACGCTCTCCCAGC

**2 The dual luciferase reporter assay for Col1a1 and miR-185-3p**

1) Schematic diagram of the pMIR-REPORT plasmid


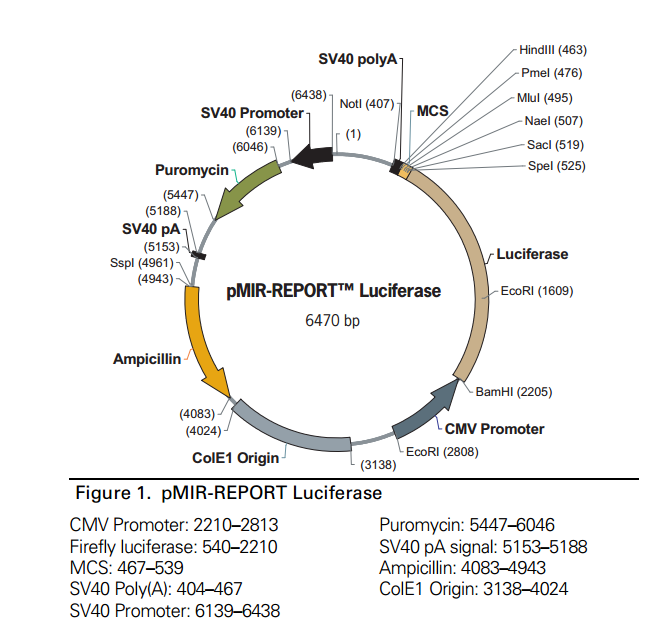


The pMIR-REPORT luciferase reporter plasmid was used to construct luciferase reporter plasmids of Col1a1. The Col1a1 3'UTR-WT-pMIR and Col1a1 3'UTR MUT-pMIR were cloned into the Mlu I和Hind Ⅲ sites of pMIR-REPORT plasmid.

2)plasmid sequences

Col1a1 3'UTR-WT-pMIR

1 actccctcc atcccaacct ggctccctcc cacccaacca

40 actttccccc caacccggaa acagacaagc aacccaaact gaaccccctc aaaagccaaa

100 aaatgggaga caatttcaca tggactttgg aaaatatttt tttcctttgc attcatctct

160 caaacttagt ttttatcttt gaccaaccga acatgaccaa aaaccaaaag tgcattcaac

220 cttaccaaaa aaaaaaaaaa aaaaagaata aataaataac tttttaaaaa aggaagcttg

280 gtccacttgc ttgaagaccc atgcgggggt aagtcccttt ctgcccgttg ggcttatgaa

340 accccaatgc tgccctttct gctcctttct ccacaccccc cttggggcct cccctccact

400 ccttcccaaa tctgtctccc cagaagacac aggaaacaat gtattgtctg cccagcaatc

460 aaaggcaatg ctcaaacacc caagtggccc ccaccctcag cccgctcctg cccgcccagc

520 acccccaggc cctgggggac ctggggttct cagactgcca aagaagcctt gccatctggc

580 gctcccatgg ctcttgcaac atctcccctt cgtttttgag ggggtcatgc cgggggagcc

640 accagcccct cactgggttc ggaggagagt caggaagggc cacgacaaag cagaaacatc

700 ggatttgggg aacgcgtgtc aatcccttgt gccgcagggc tgggcgggag agactgttct

760 gttccttgtg taactgtgtt gctgaaagac tacctcgttc ttgtcttgat gtgtcaccgg

820 ggcaactgcc tgggggcggg gatgggggca gggtggaagc ggctccccat tttataccaa

880 aggtgctaca tctatgtgat gggtggggtg gggagggaat cactggtgct atagaaattg

940 agatgccccc ccaggccagc aaatgttcct ttttgttcaa agtctatttt tattccttga

1000 tatttttctt tttttttttt tttttttgtg gatggggact tgtgaatttt tctaaaggtg

1060 ctatttaaca tgggaggaga gcgtgtgcgg ctccagccca gcccgctgct cactttccac

1120cctctctcca cctgcctctg gcttctcagg cctctgctct ccgacctctc tcctctgaaa

1180 ccctcctcca cagctgcagc ccatcctccc ggctccctcc tagtctgtcc tgcgtcctct

1240 gtccccgggt ttcagagaca acttcccaaa gcacaaagca gtttttcccc ctaggggtgg

1300 gaggaagcaa aagactctgt acctattttg tatgtgtata ataatttgag atgtttttaa

1360 ttattttgat tgctggaata aagcatgtgg aaatgaccca aacataa

Col1a1 3'UTR MUT-pMIR (CAGCCCC to UCCGGGG) ：

1 actccctcc atcccaacct ggctccctcc cacccaacca

40 actttccccc caacccggaa acagacaagc aacccaaact gaaccccctc aaaagccaaa

100 aaatgggaga caatttcaca tggactttgg aaaatatttt tttcctttgc attcatctct

160 caaacttagt ttttatcttt gaccaaccga acatgaccaa aaaccaaaag tgcattcaac

220 cttaccaaaa aaaaaaaaaa aaaaagaata aataaataac tttttaaaaa aggaagcttg

280 gtccacttgc ttgaagaccc atgcgggggt aagtcccttt ctgcccgttg ggcttatgaa

340 accccaatgc tgccctttct gctcctttct ccacaccccc cttggggcct cccctccact

400 ccttcccaaa tctgtctccc cagaagacac aggaaacaat gtattgtctg cccagcaatc

460 aaaggcaatg ctcaaacacc caagtggccc ccaccctcag cccgctcctg cccgcccagc

520 acccccaggc cctgggggac ctggggttct cagactgcca aagaagcctt gccatctggc

580 gctcccatgg ctcttgcaac atctcccctt cgtttttgag ggggtcatgc cgggggagcc

640 acuccggggt cactgggttc ggaggagagt caggaagggc cacgacaaag cagaaacatc

700 ggatttgggg aacgcgtgtc aatcccttgt gccgcagggc tgggcgggag agactgttct

760 gttccttgtg taactgtgtt gctgaaagac tacctcgttc ttgtcttgat gtgtcaccgg

820 ggcaactgcc tgggggcggg gatgggggca gggtggaagc ggctccccat tttataccaa

880 aggtgctaca tctatgtgat gggtggggtg gggagggaat cactggtgct atagaaattg

940 agatgccccc ccaggccagc aaatgttcct ttttgttcaa agtctatttt tattccttga

1000 tatttttctt tttttttttt tttttttgtg gatggggact tgtgaatttt tctaaaggtg

1060 ctatttaaca tgggaggaga gcgtgtgcgg ctccagccca gcccgctgct cactttccac

1120cctctctcca cctgcctctg gcttctcagg cctctgctct ccgacctctc tcctctgaaa

1180 ccctcctcca cagctgcagc ccatcctccc ggctccctcc tagtctgtcc tgcgtcctct

1240 gtccccgggt ttcagagaca acttcccaaa gcacaaagca gtttttcccc ctaggggtgg

1300 gaggaagcaa aagactctgt acctattttg tatgtgtata ataatttgag atgtttttaa

1360 ttattttgat tgctggaata aagcatgtgg aaatgaccca aacataa
