# Supplementary material for: Suppressing circ_0008494 inhibits HSCs activation by regulating the miR-185-3p/Col1a1 axis
Source: Front Pharmacol. 2022 Nov 17;13:1050093. doi: 10.3389/fphar.2022.1050093 (PMC9713816; doi:10.3389/fphar.2022.1050093)

Fig.3de

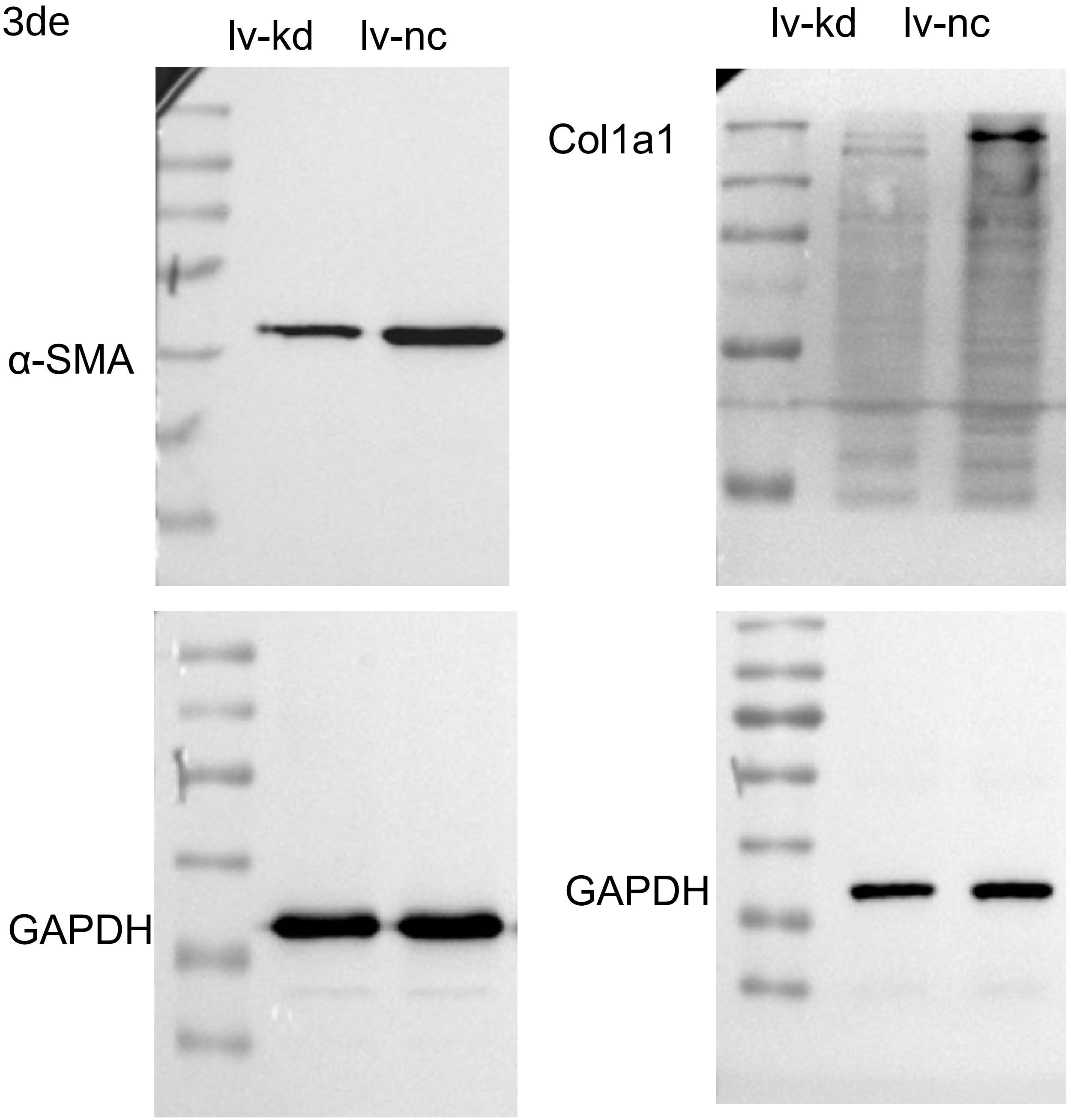

Fig.5c

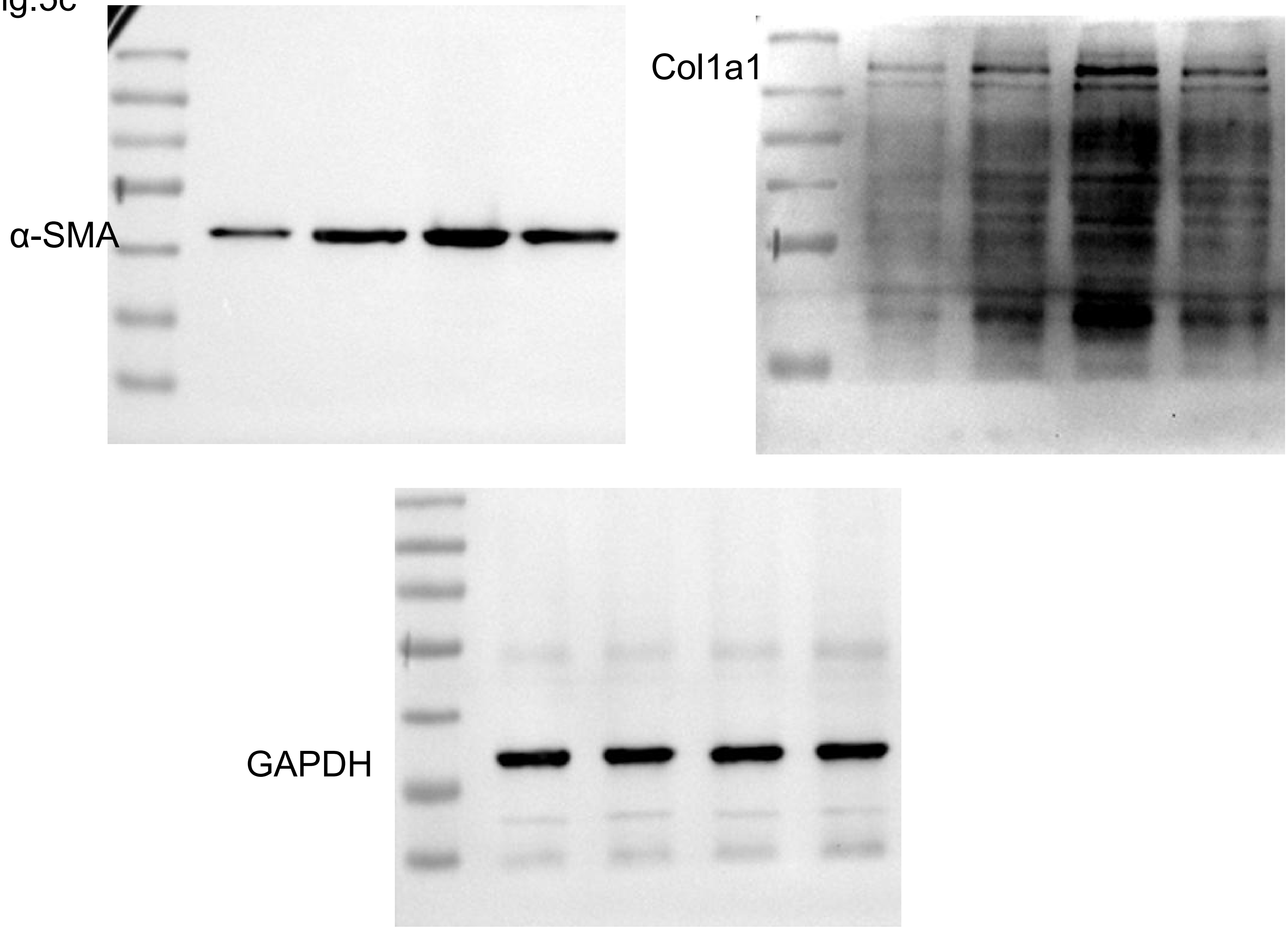

Fig.6ab

α-SMA

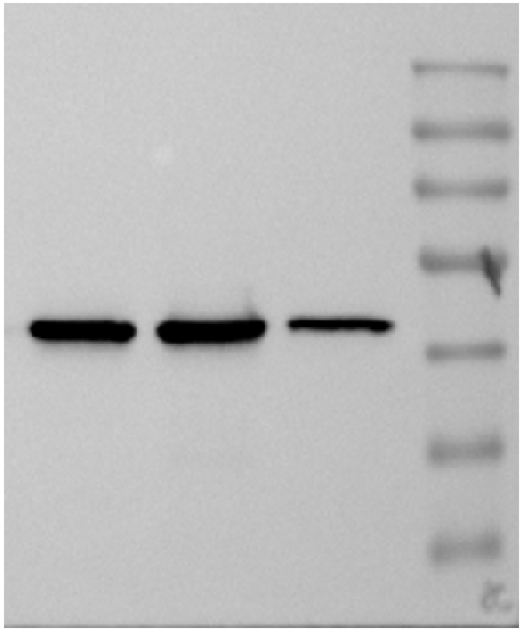

Col1a1

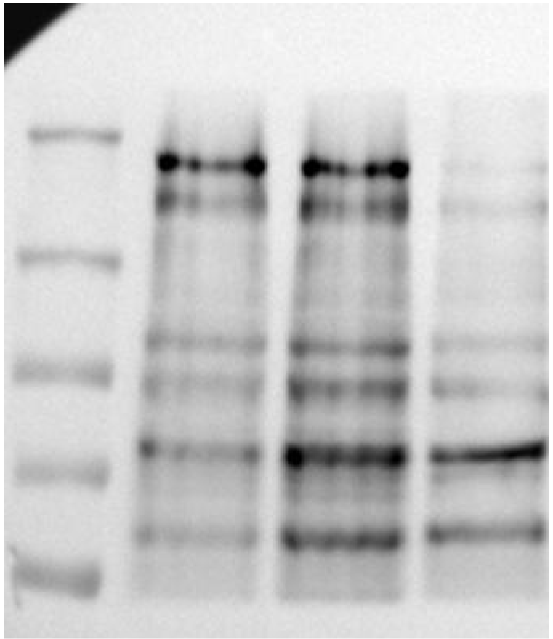

GAPDH

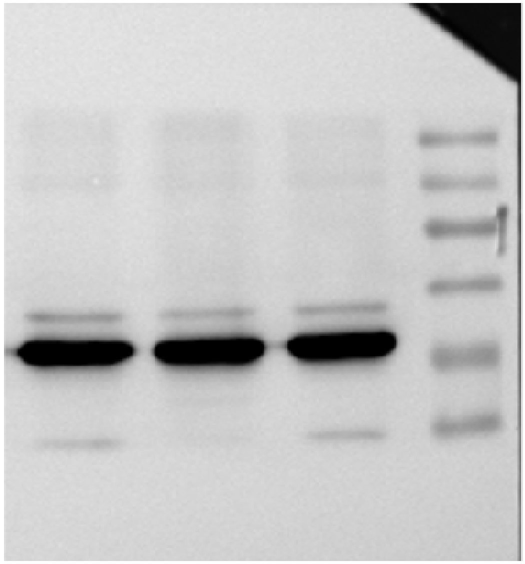

GAPDH

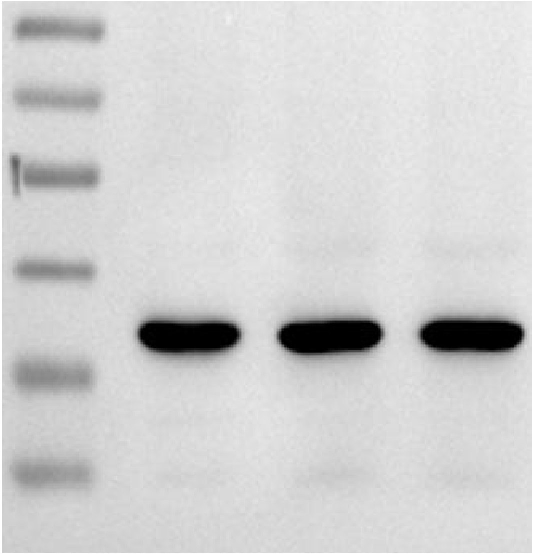

Fig.7d

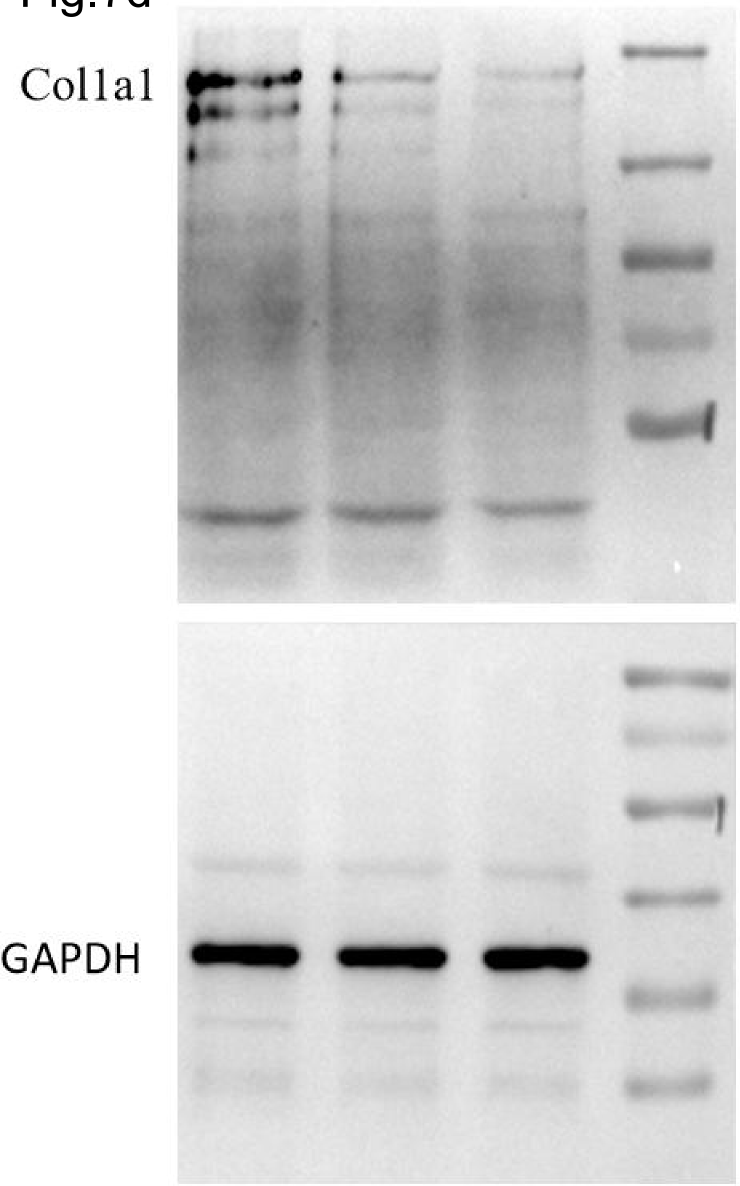

Fig.7e

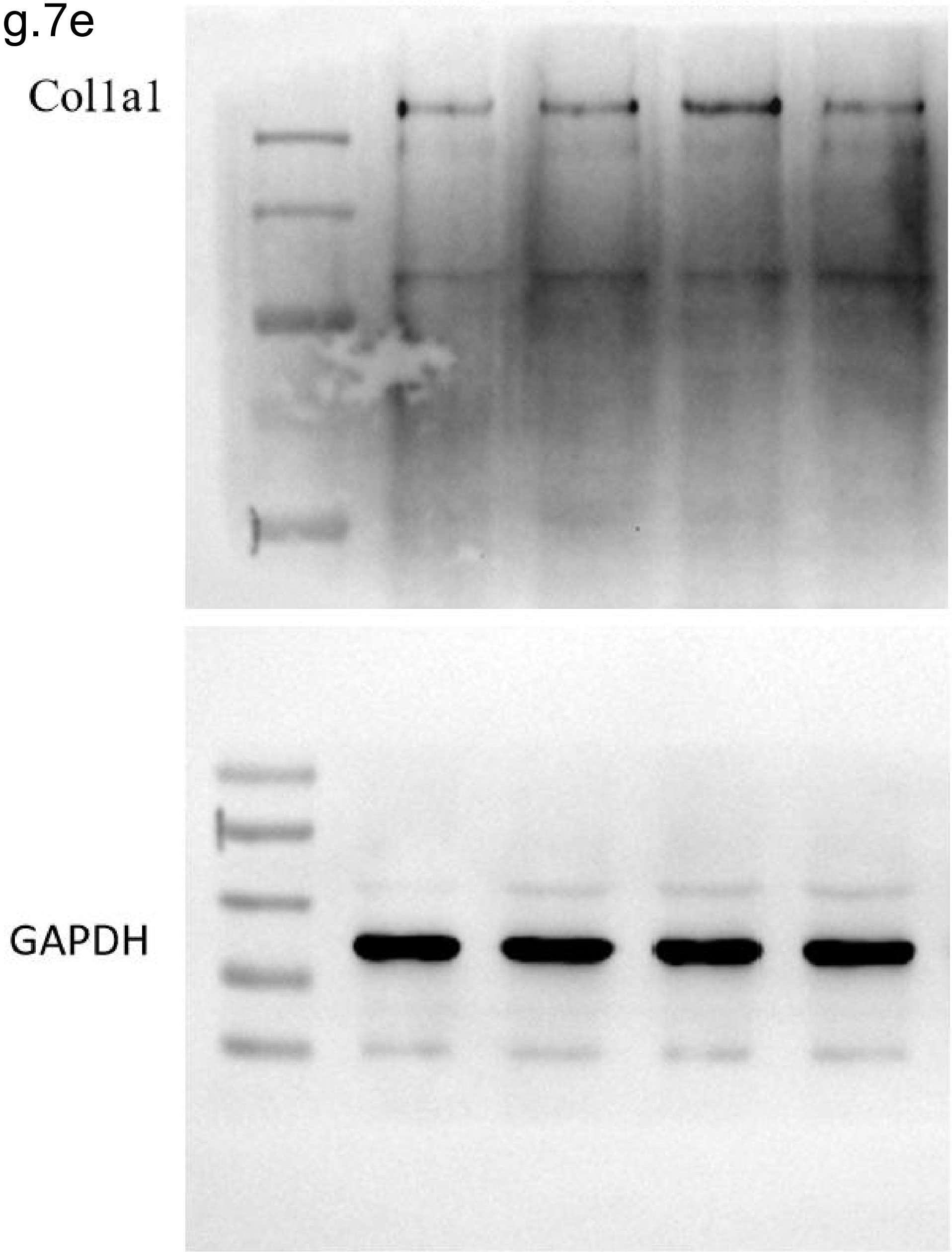

Fig.7f

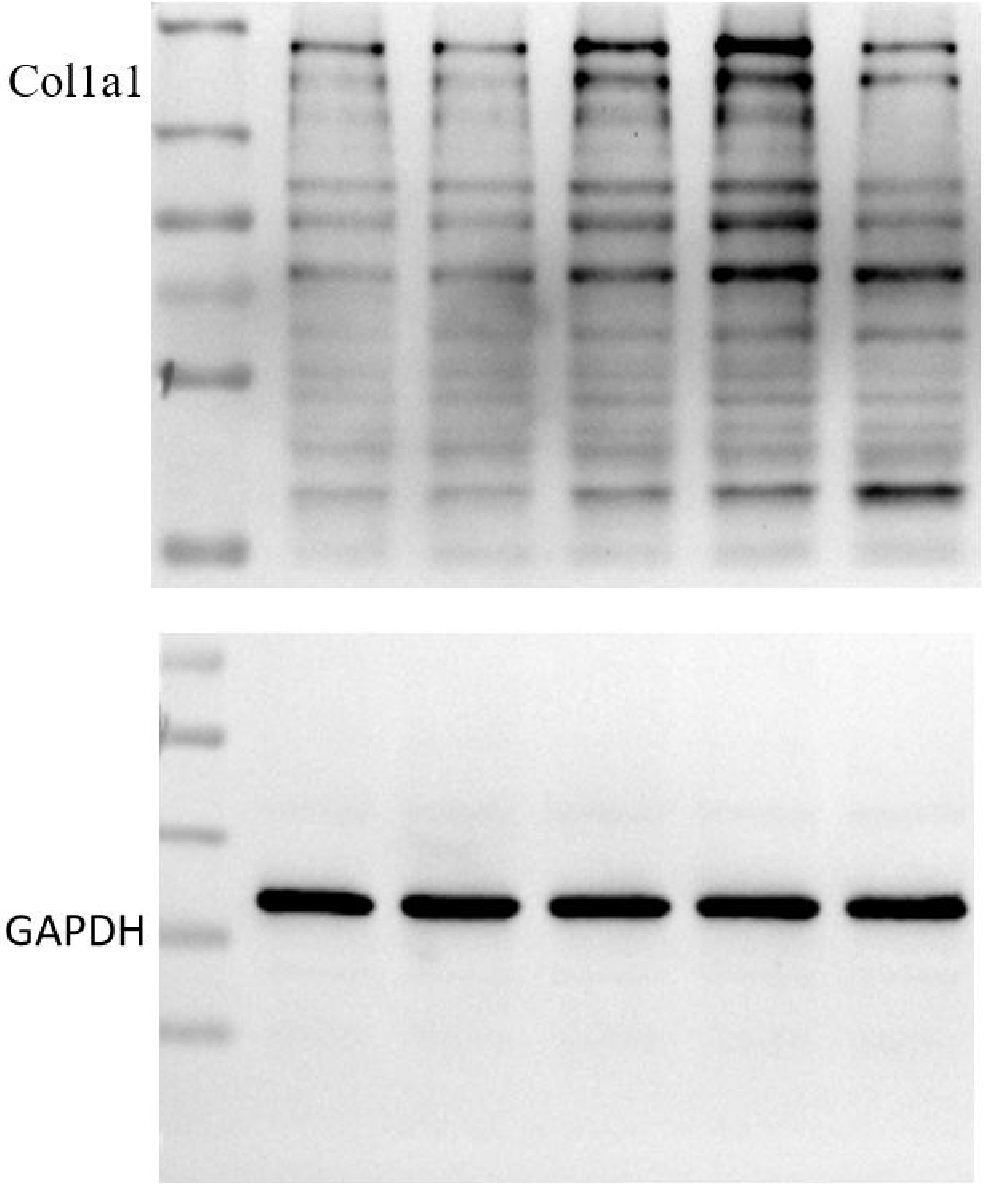

Fig.S7

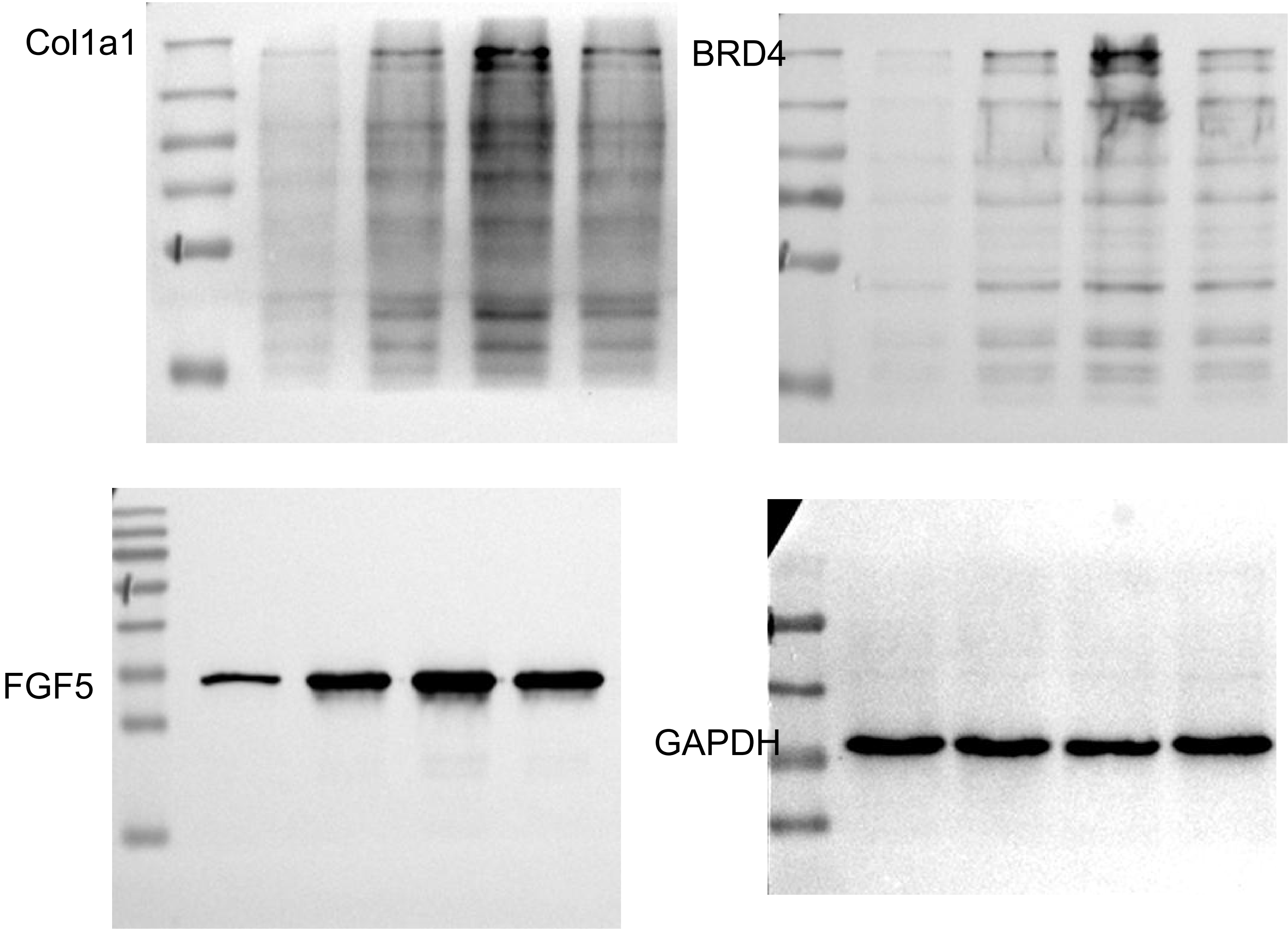

Supplement: Supplementary file 3 [file DataSheet1.ZIP › raw data1/wb/wb.pdf]
