# Supplementary figures and images for: Suppressing circ_0008494 inhibits HSCs activation by regulating the miR-185-3p/Col1a1 axis
Source: Front Pharmacol. 2022 Nov 17;13:1050093. doi: 10.3389/fphar.2022.1050093 (PMC9713816; doi:10.3389/fphar.2022.1050093)

COL1a1
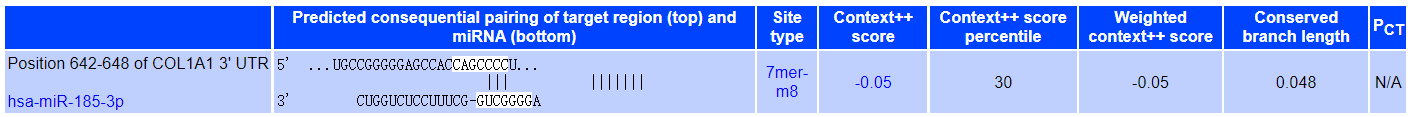


FGF5


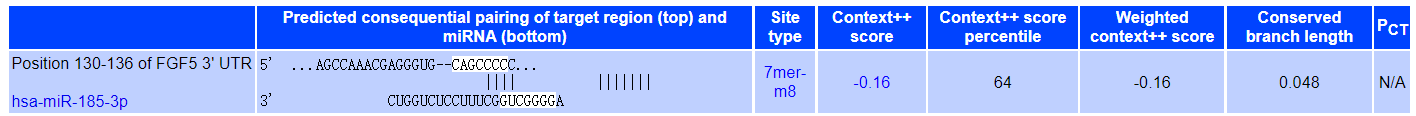


TIMP2


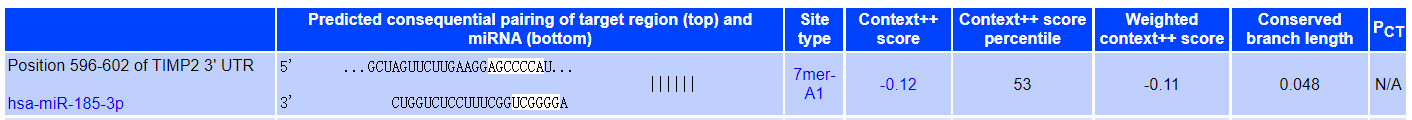


TGFBR2


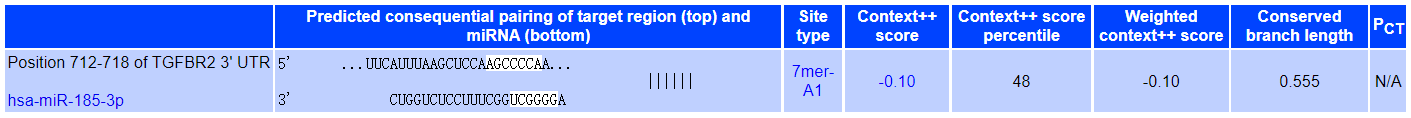

Supplement: Supplementary file 3 [file DataSheet1.ZIP › raw data1/Bioinformatics/supple Table3 Predicted mRNA.docx]

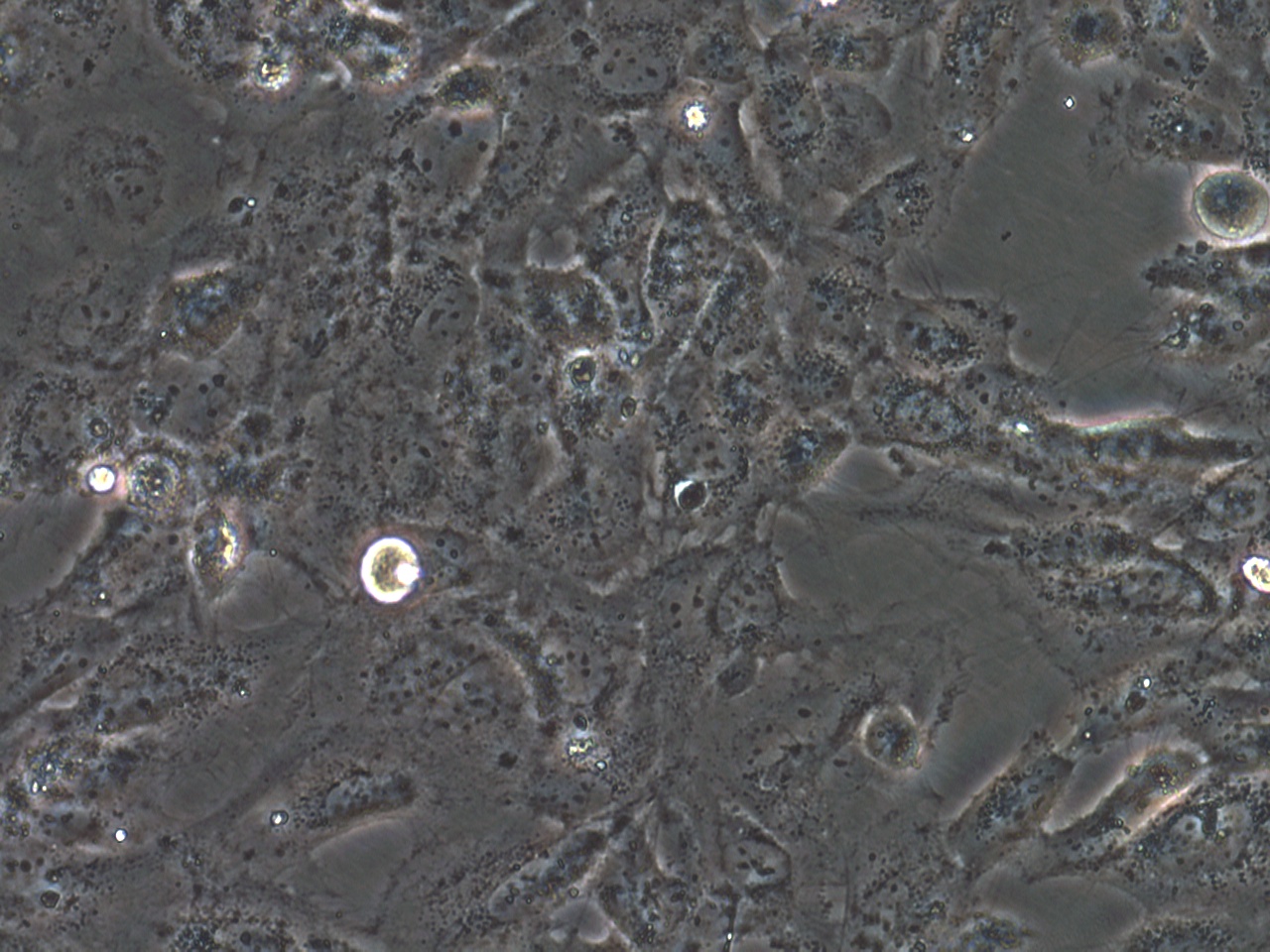

Supplement: Supplementary file 3 [file DataSheet1.ZIP › raw data1/Figure3B Immunofluorescence/KD1 B.jpg]

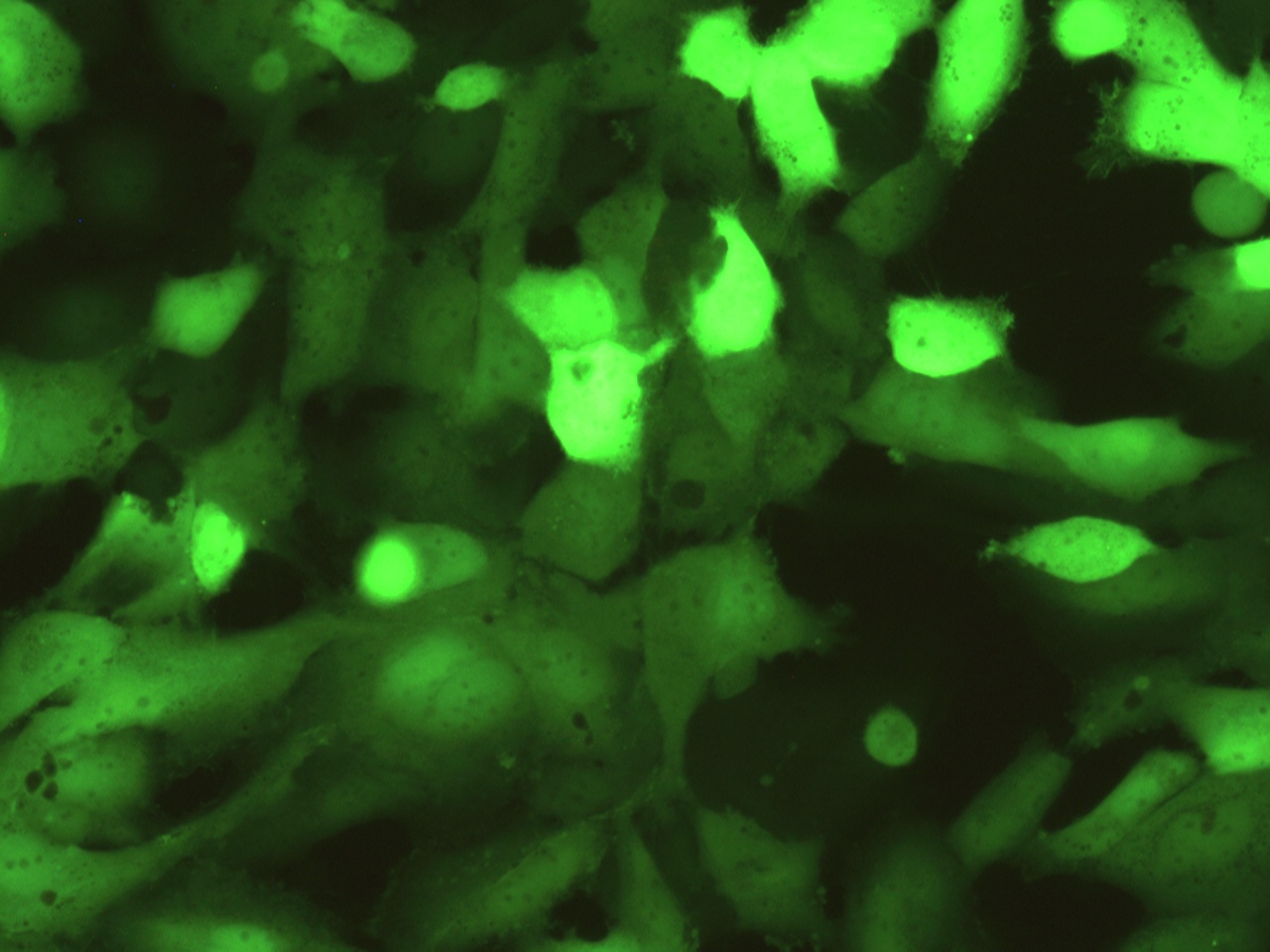

Supplement: Supplementary file 3 [file DataSheet1.ZIP › raw data1/Figure3B Immunofluorescence/KD1 G.jpg]

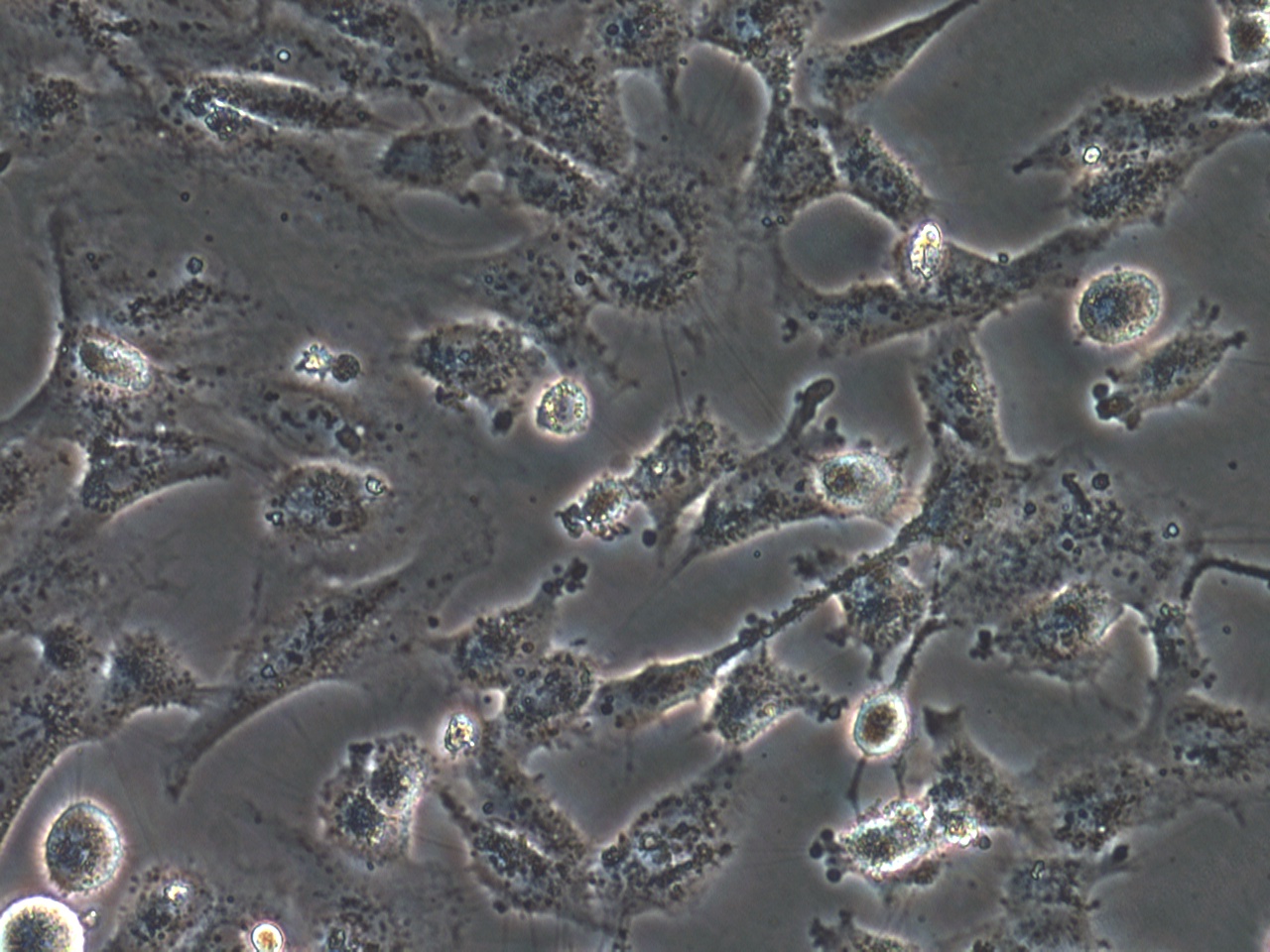

Supplement: Supplementary file 3 [file DataSheet1.ZIP › raw data1/Figure3B Immunofluorescence/KD2 B.jpg]

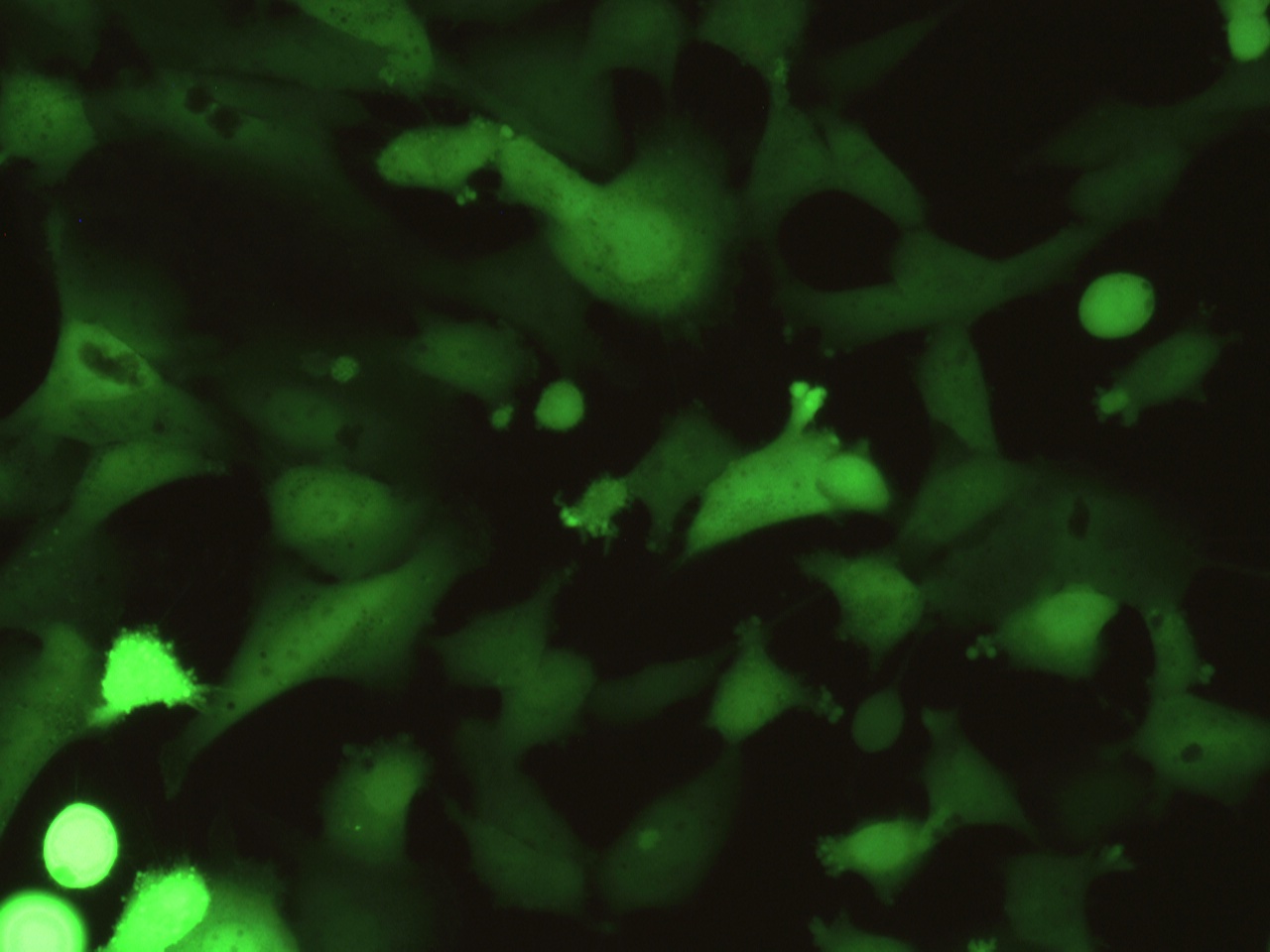

Supplement: Supplementary file 3 [file DataSheet1.ZIP › raw data1/Figure3B Immunofluorescence/KD2 G.jpg]

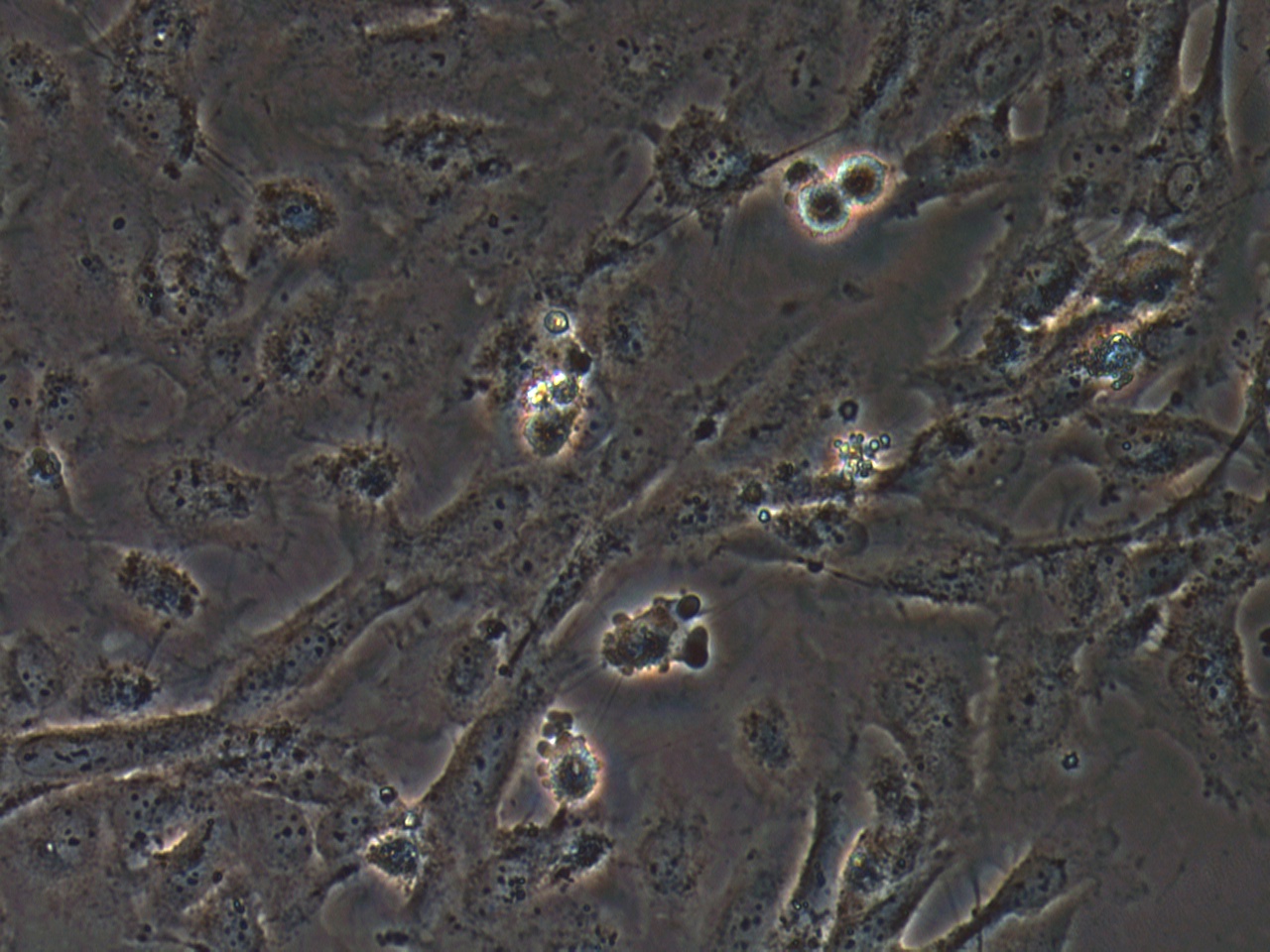

Supplement: Supplementary file 3 [file DataSheet1.ZIP › raw data1/Figure3B Immunofluorescence/KD3 B.jpg]

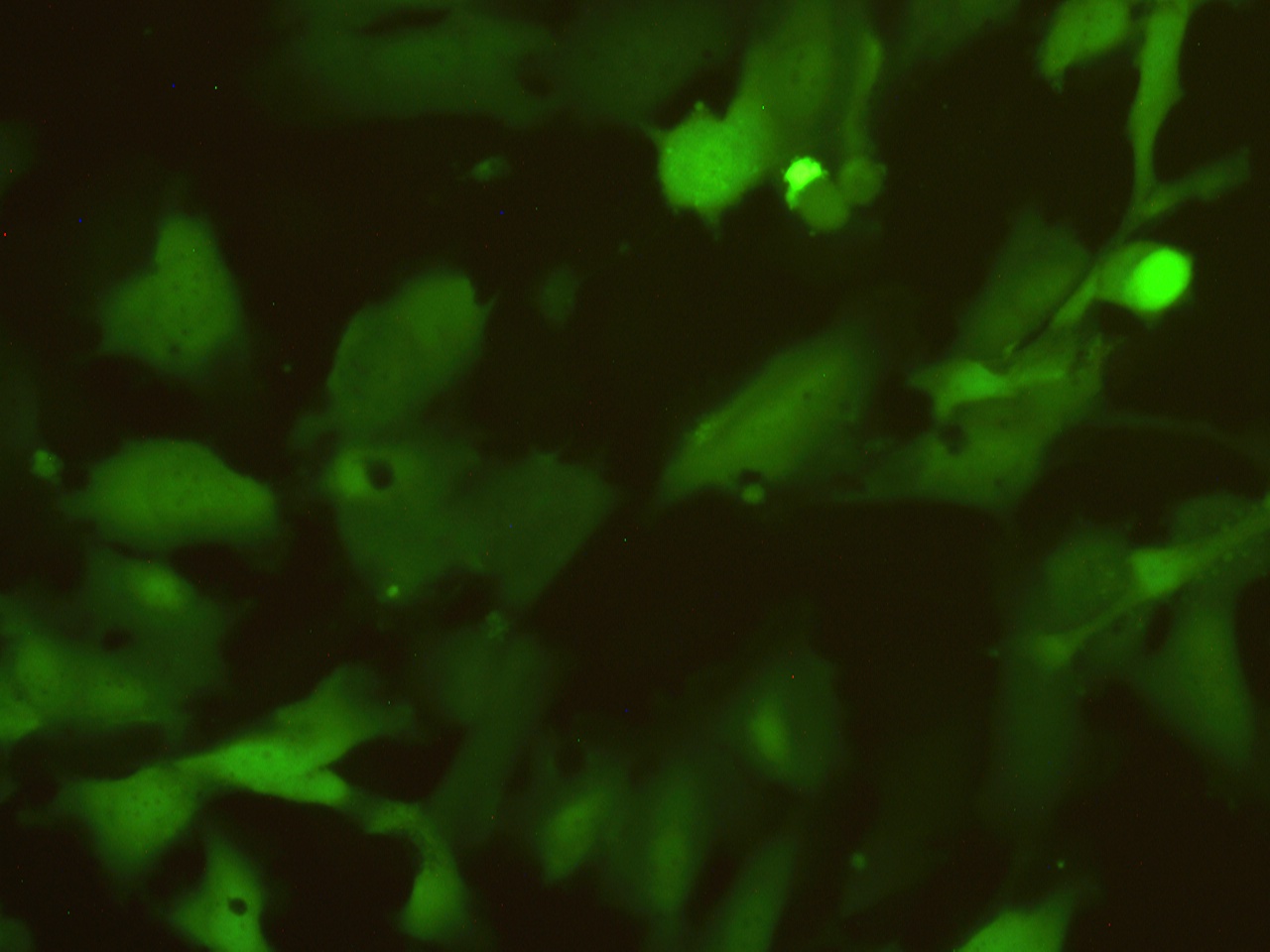

Supplement: Supplementary file 3 [file DataSheet1.ZIP › raw data1/Figure3B Immunofluorescence/KD3 G.jpg]

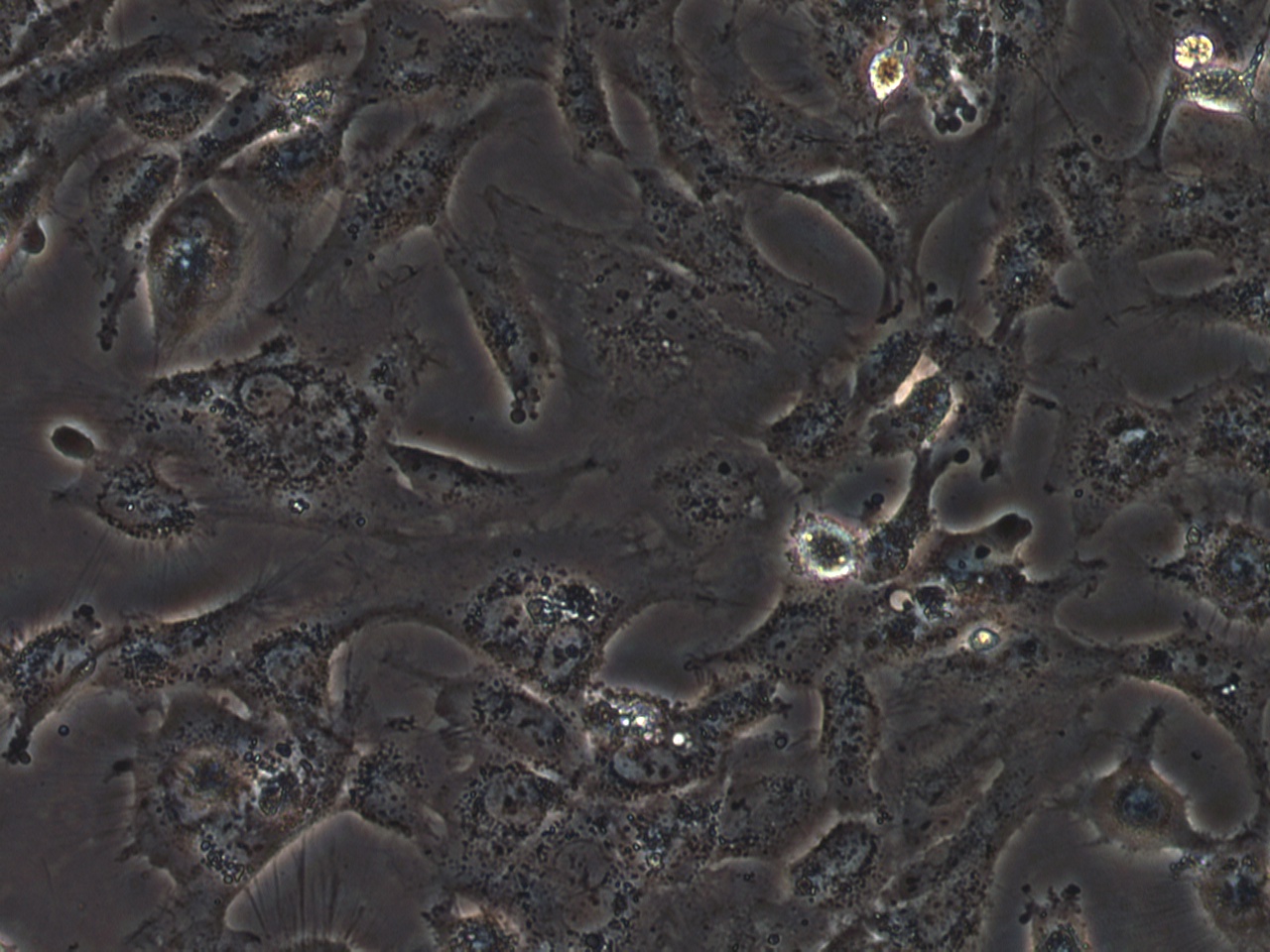

Supplement: Supplementary file 3 [file DataSheet1.ZIP › raw data1/Figure3B Immunofluorescence/NC B.jpg]

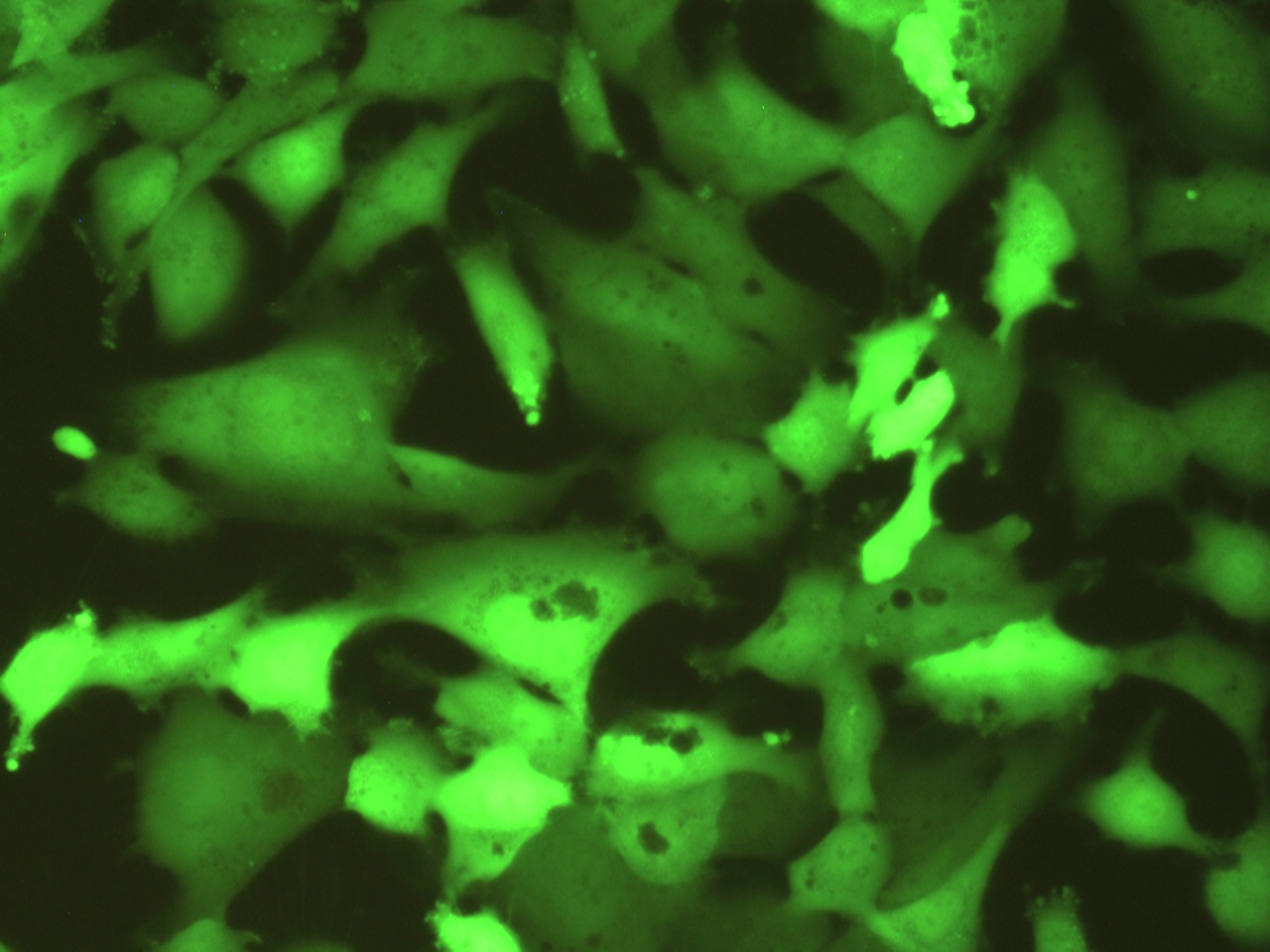

Supplement: Supplementary file 3 [file DataSheet1.ZIP › raw data1/Figure3B Immunofluorescence/NC G.jpg]

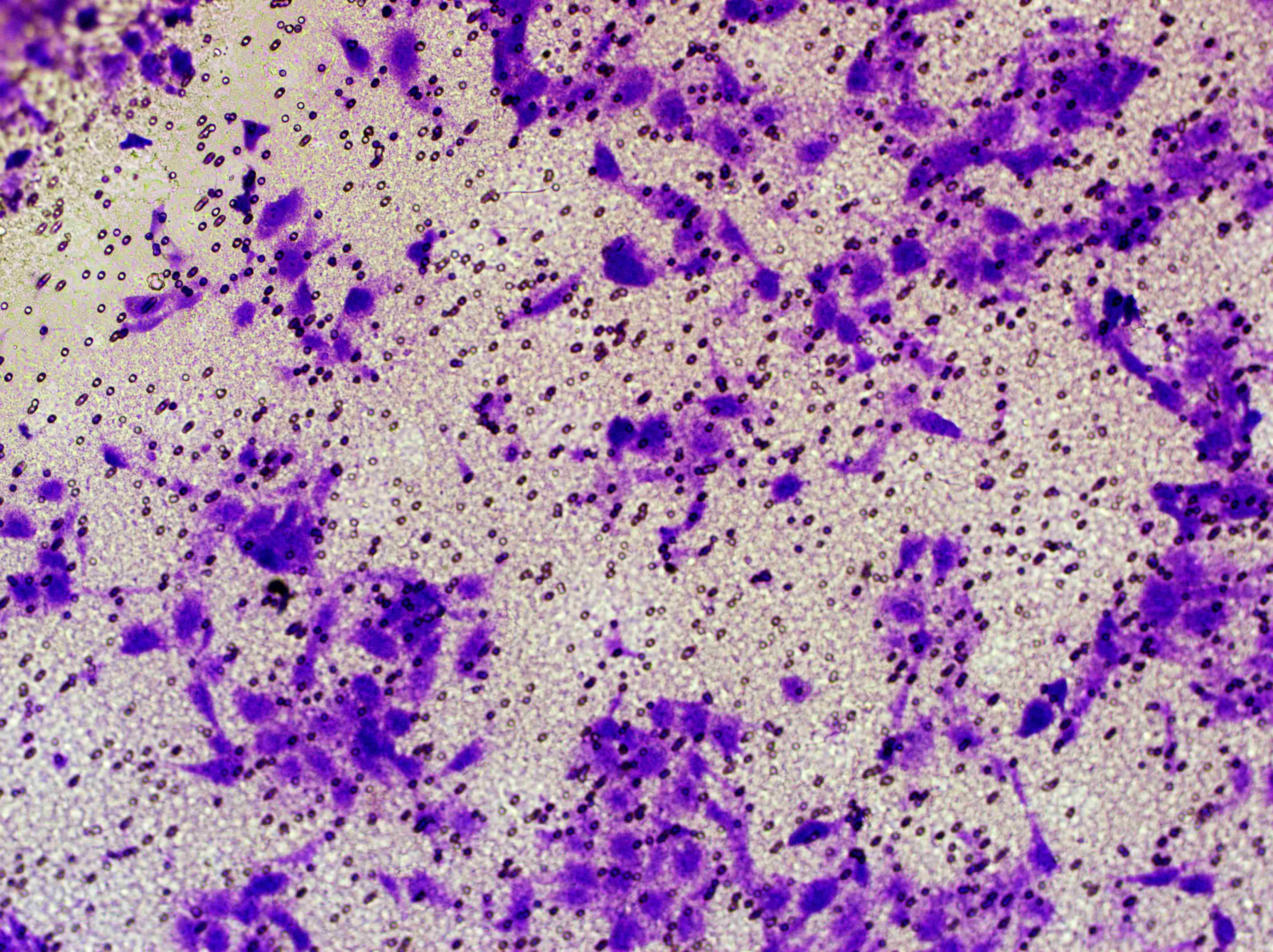

Supplement: Supplementary file 4 [file DataSheet2.ZIP › raw data2/Figur3G 5E 6D transwell/lv-circ0008494/LV-KD1.jpg]

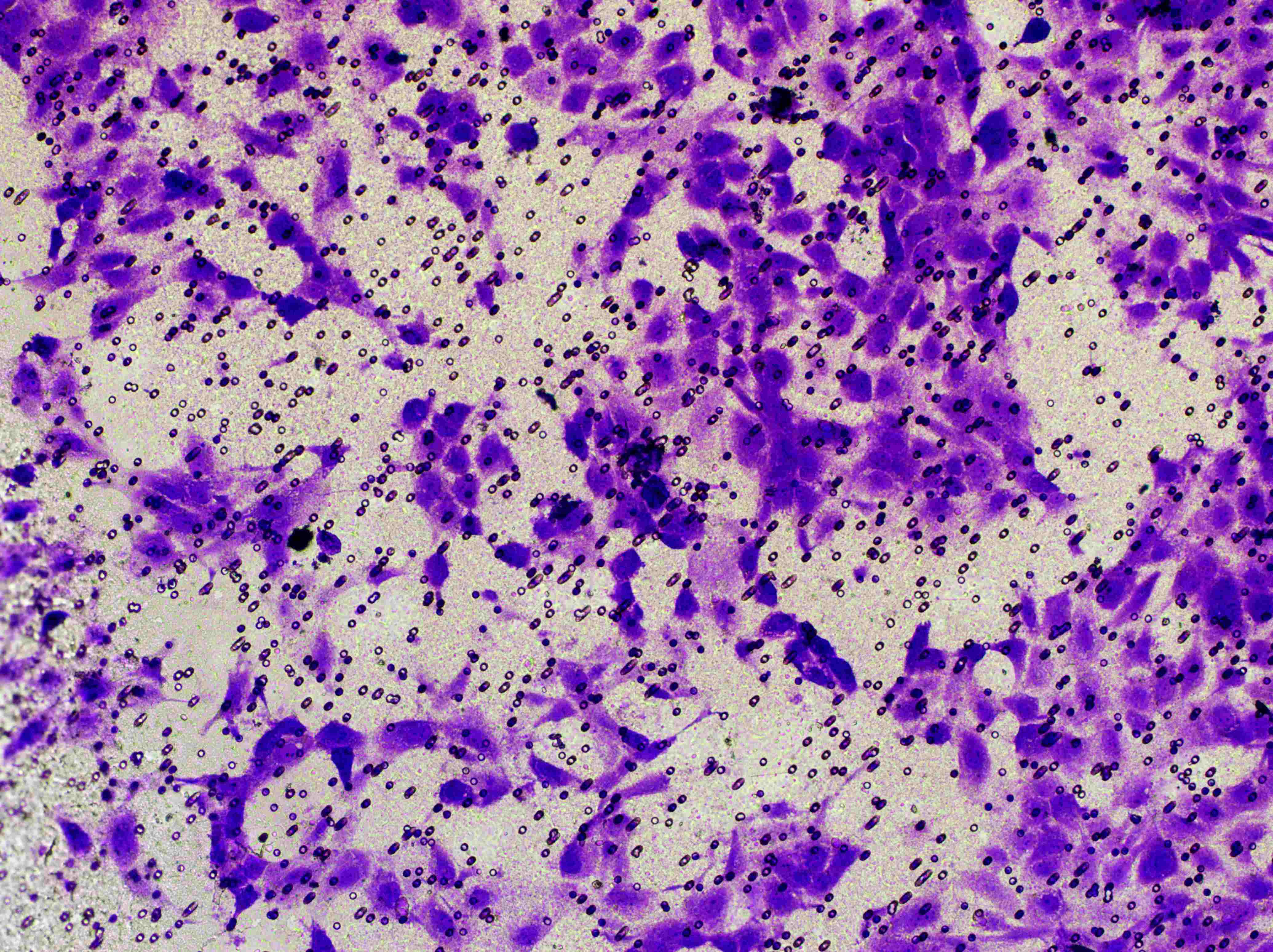

Supplement: Supplementary file 4 [file DataSheet2.ZIP › raw data2/Figur3G 5E 6D transwell/lv-circ0008494/LV-KD2.jpg]

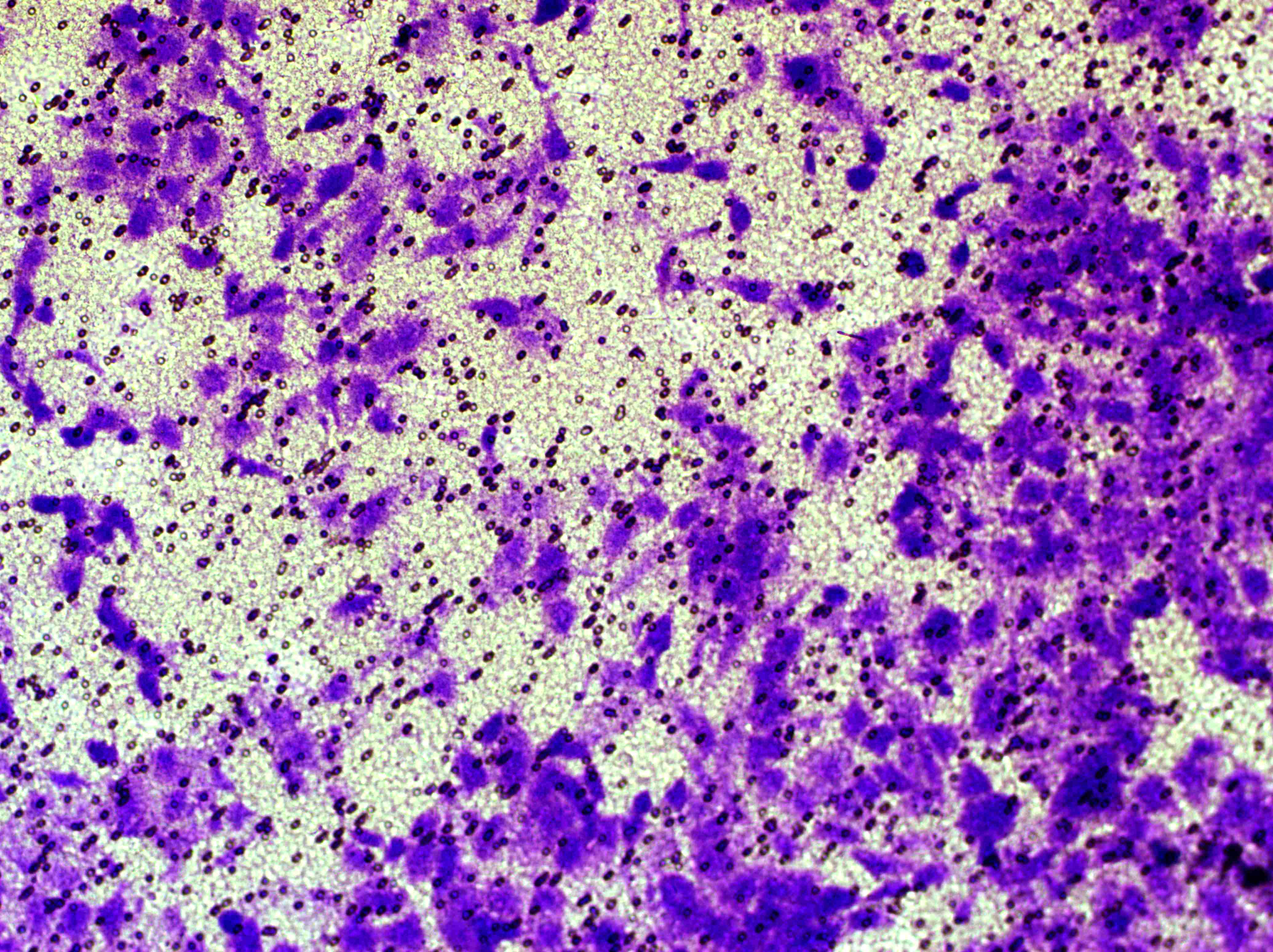

Supplement: Supplementary file 4 [file DataSheet2.ZIP › raw data2/Figur3G 5E 6D transwell/lv-circ0008494/LV-KD3.jpg]

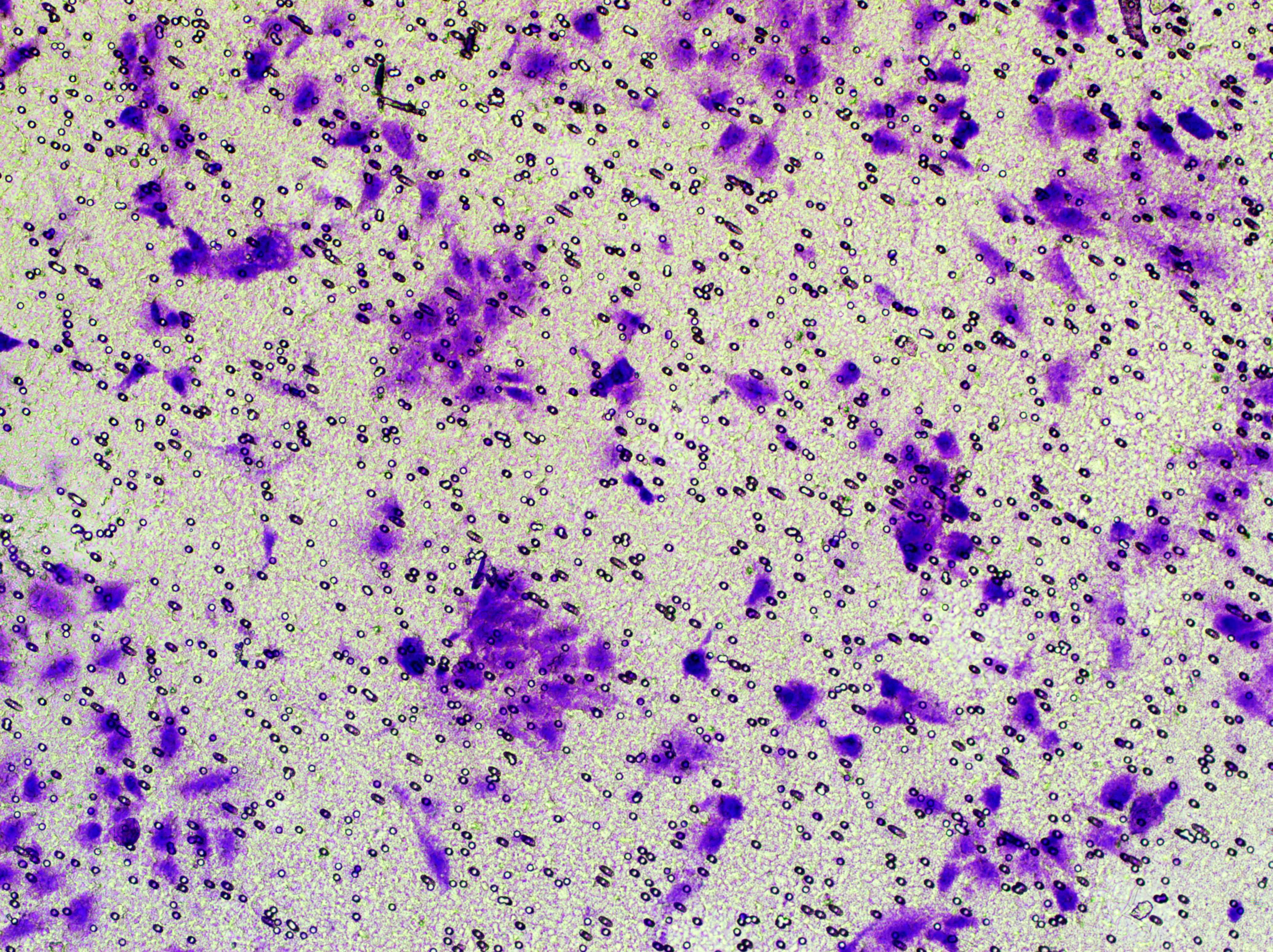

Supplement: Supplementary file 4 [file DataSheet2.ZIP › raw data2/Figur3G 5E 6D transwell/lv-circ0008494/LV-NC1.jpg]

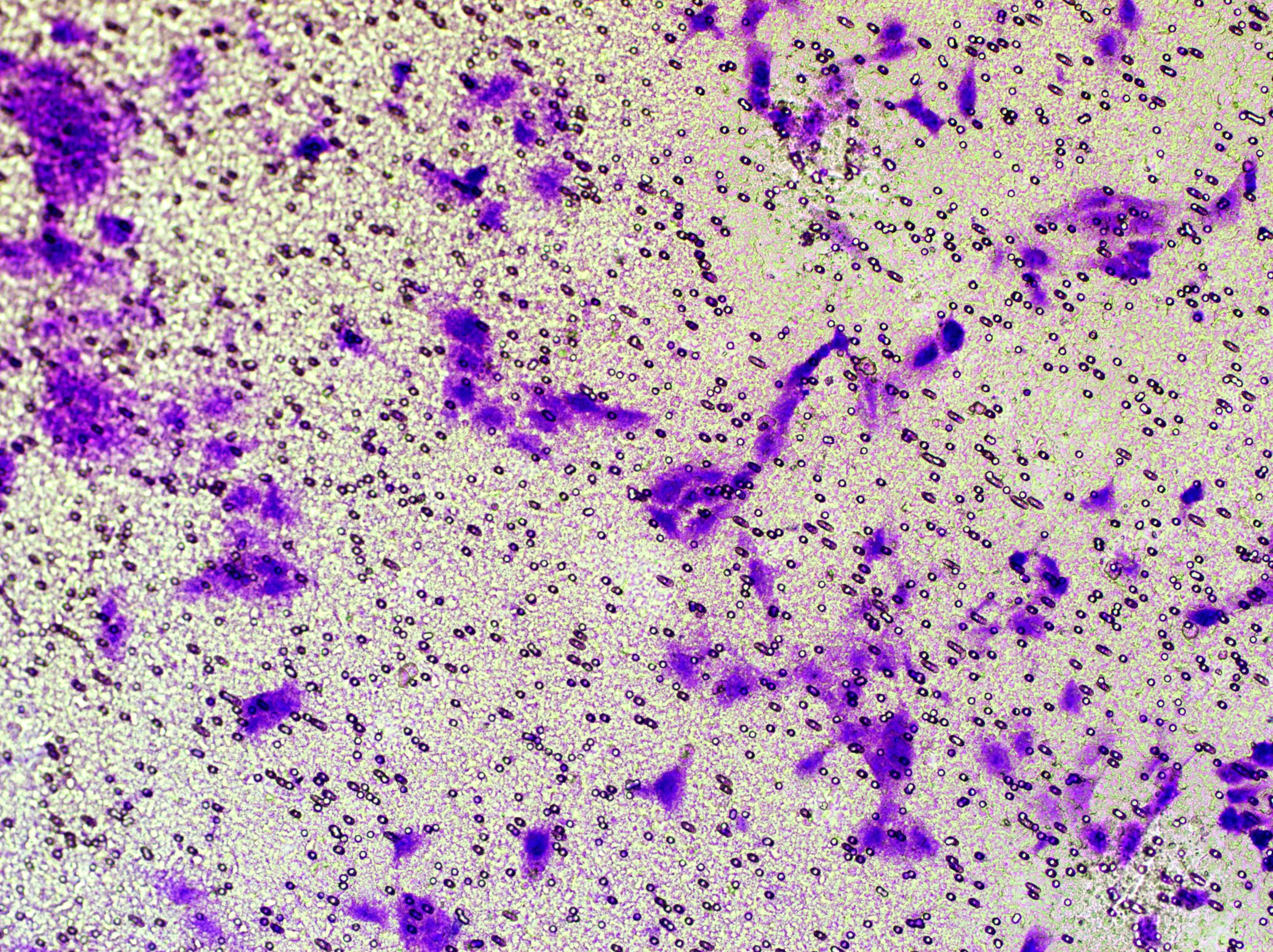

Supplement: Supplementary file 4 [file DataSheet2.ZIP › raw data2/Figur3G 5E 6D transwell/lv-circ0008494/LV-NC2.jpg]

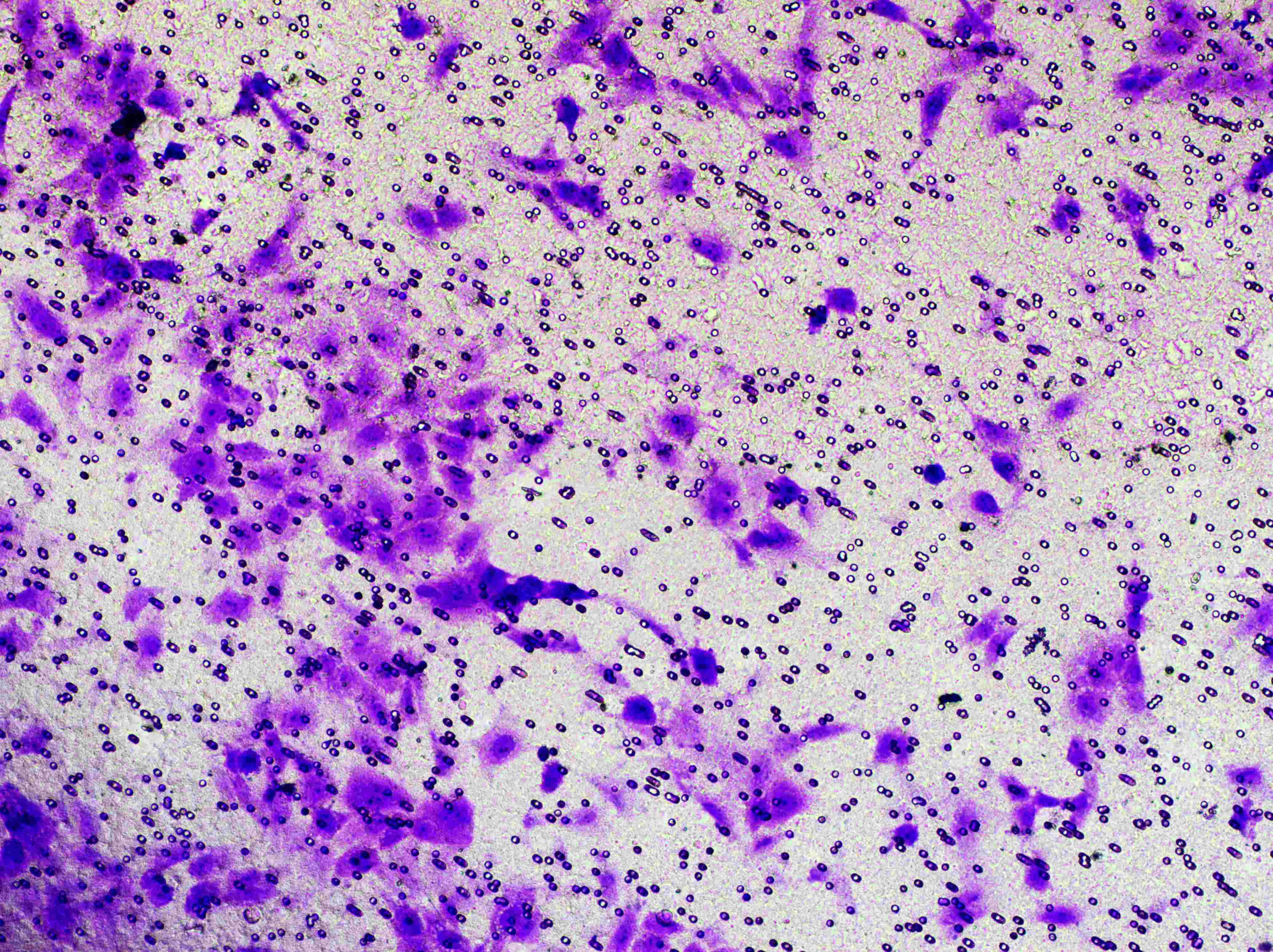

Supplement: Supplementary file 4 [file DataSheet2.ZIP › raw data2/Figur3G 5E 6D transwell/lv-circ0008494/LV-NC3.jpg]

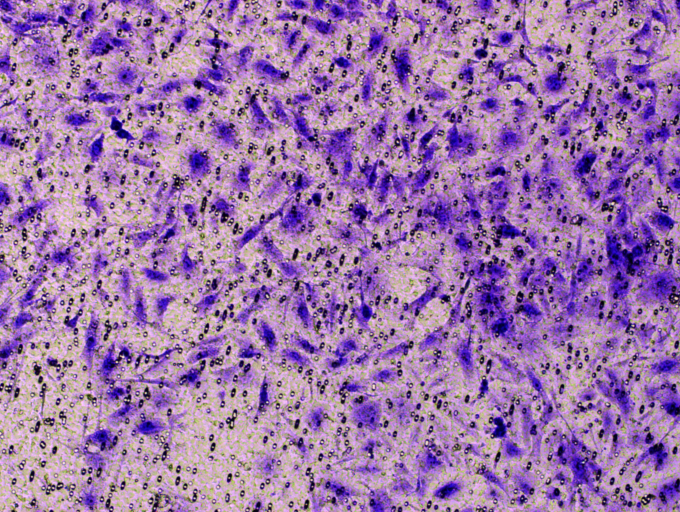

Supplement: Supplementary file 4 [file DataSheet2.ZIP › raw data2/Figur3G 5E 6D transwell/miR-185/185 inhibitor nc1.png]

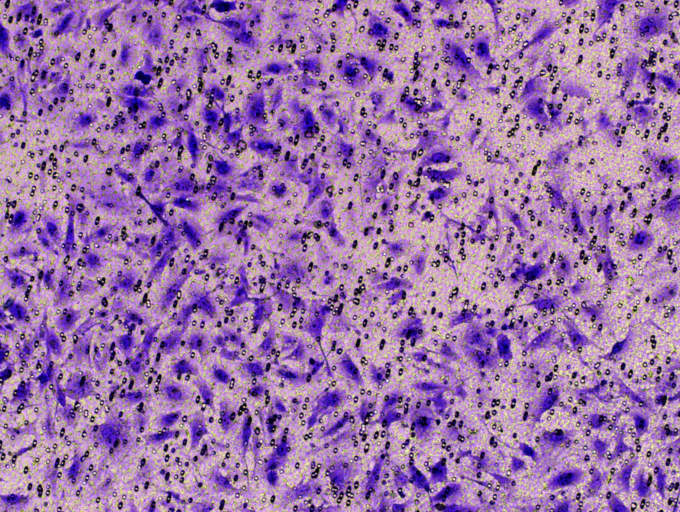

Supplement: Supplementary file 4 [file DataSheet2.ZIP › raw data2/Figur3G 5E 6D transwell/miR-185/185 inhibitor nc2.png]

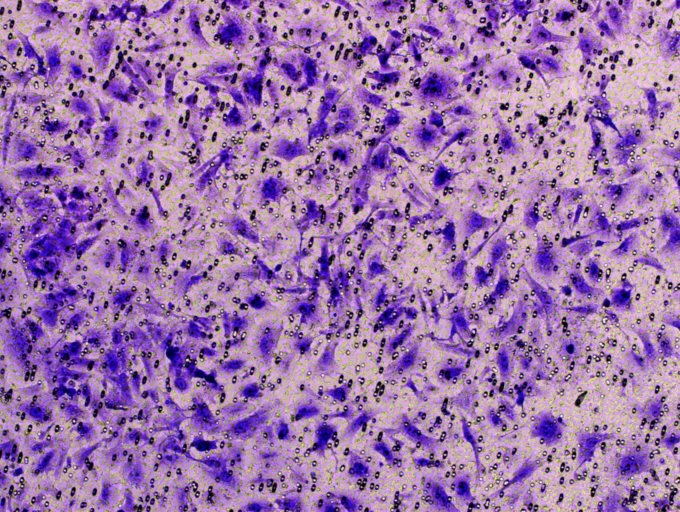

Supplement: Supplementary file 4 [file DataSheet2.ZIP › raw data2/Figur3G 5E 6D transwell/miR-185/185 inhibitor nc3.png]

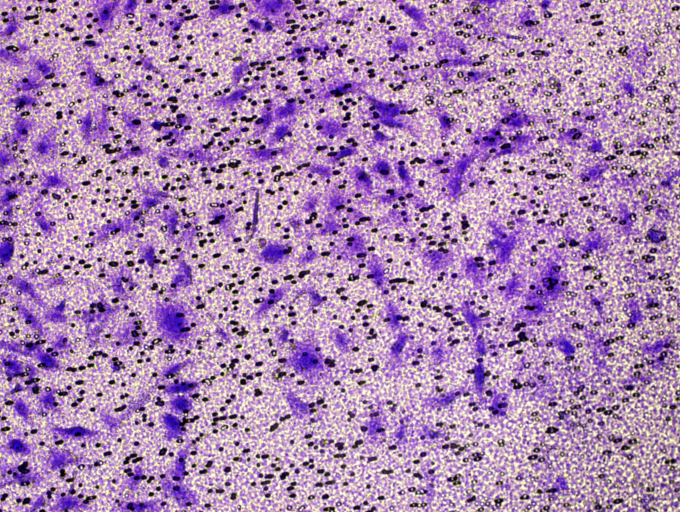

Supplement: Supplementary file 4 [file DataSheet2.ZIP › raw data2/Figur3G 5E 6D transwell/miR-185/185 inhibitor1.png]

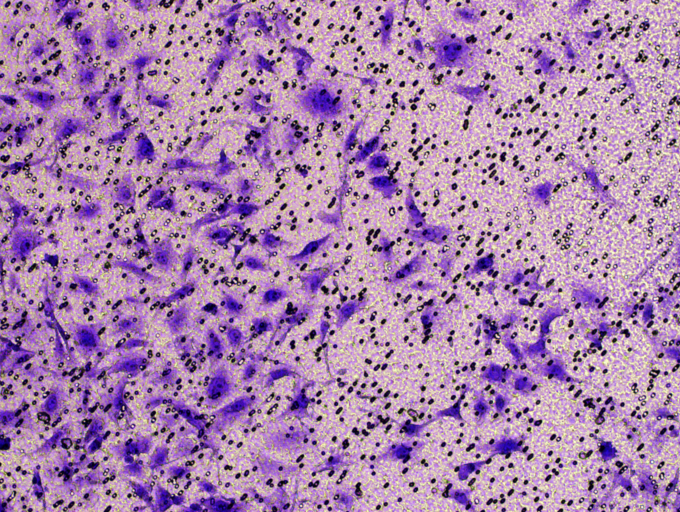

Supplement: Supplementary file 4 [file DataSheet2.ZIP › raw data2/Figur3G 5E 6D transwell/miR-185/185 inhibitor2.png]

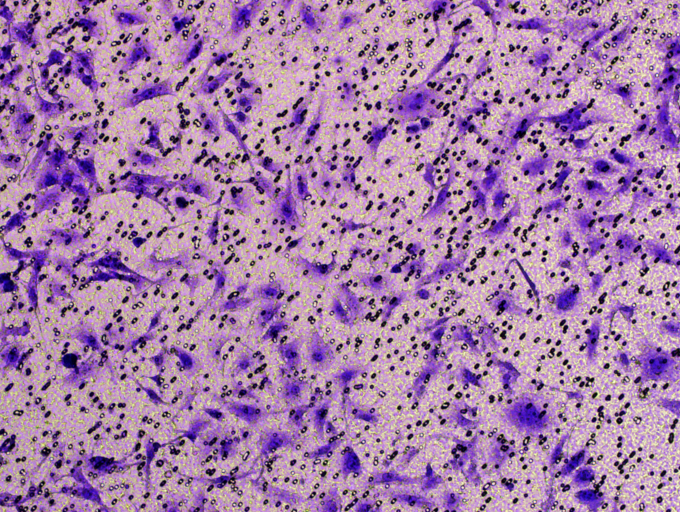

Supplement: Supplementary file 4 [file DataSheet2.ZIP › raw data2/Figur3G 5E 6D transwell/miR-185/185 inhibitor3.png]

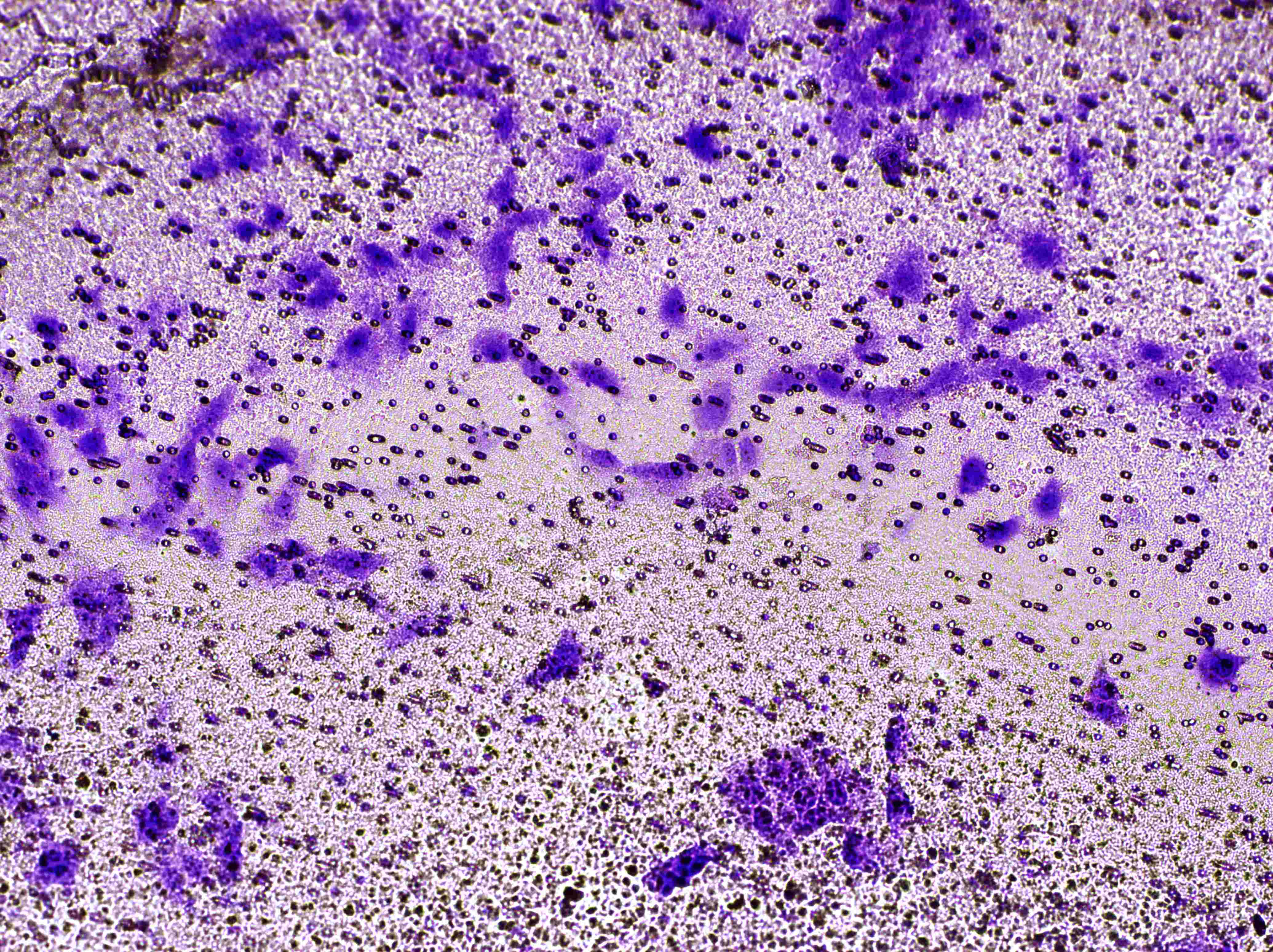

Supplement: Supplementary file 4 [file DataSheet2.ZIP › raw data2/Figur3G 5E 6D transwell/miR-185/185 mimic nc1.jpg]

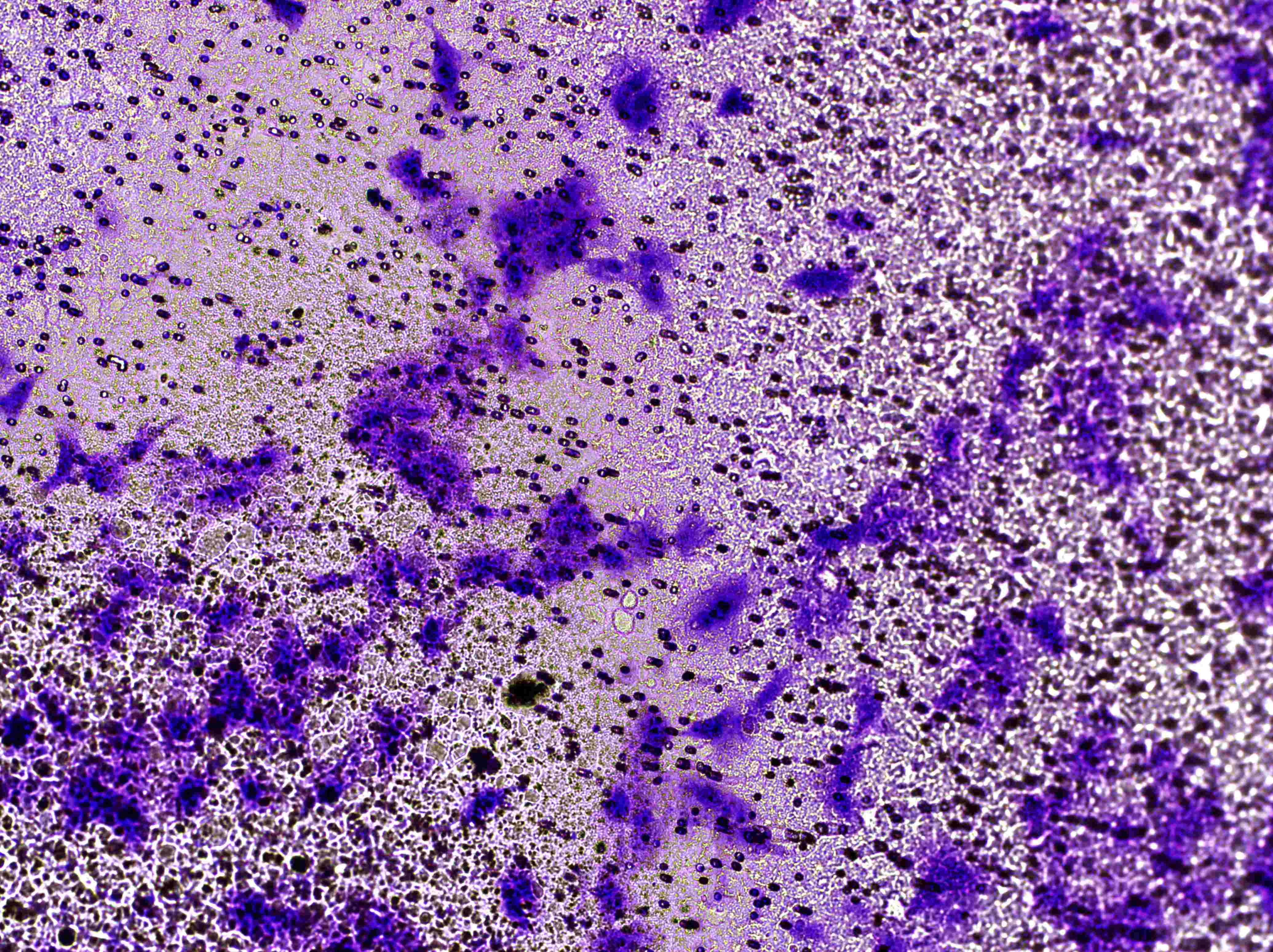

Supplement: Supplementary file 4 [file DataSheet2.ZIP › raw data2/Figur3G 5E 6D transwell/miR-185/185 mimic nc2.jpg]

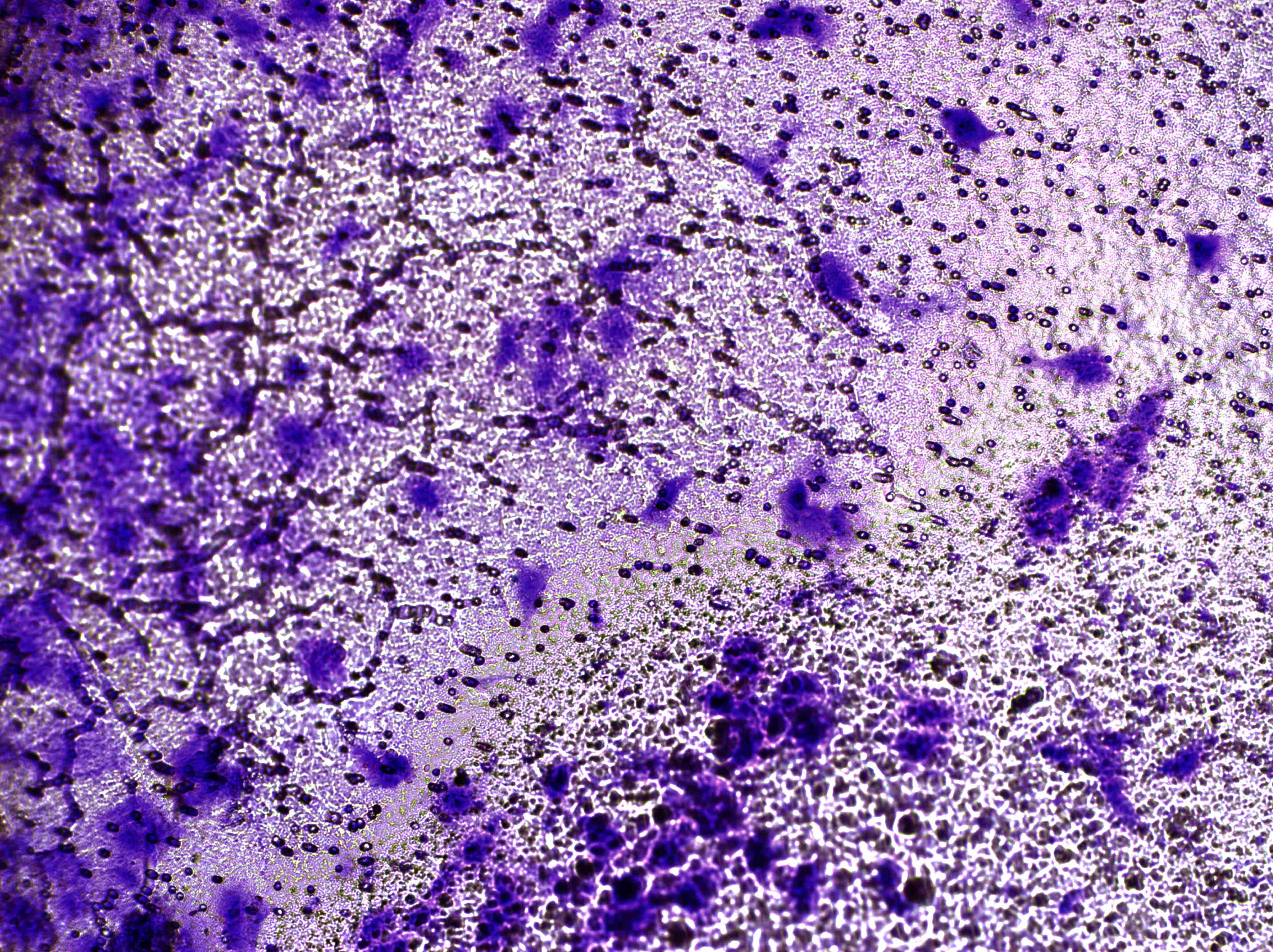

Supplement: Supplementary file 4 [file DataSheet2.ZIP › raw data2/Figur3G 5E 6D transwell/miR-185/185 mimic nc3.jpg]

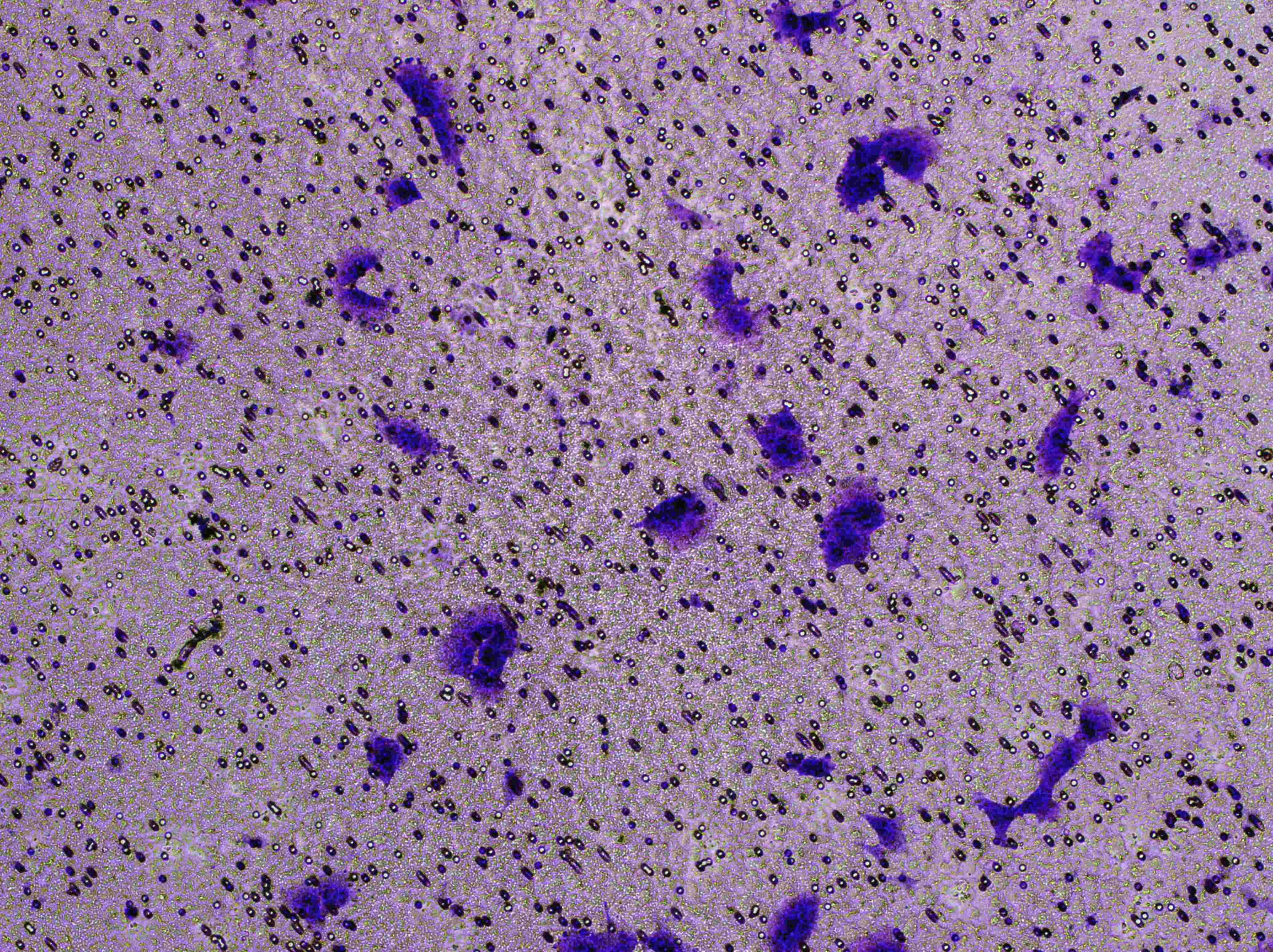

Supplement: Supplementary file 4 [file DataSheet2.ZIP › raw data2/Figur3G 5E 6D transwell/miR-185/185 mimic1.jpg]

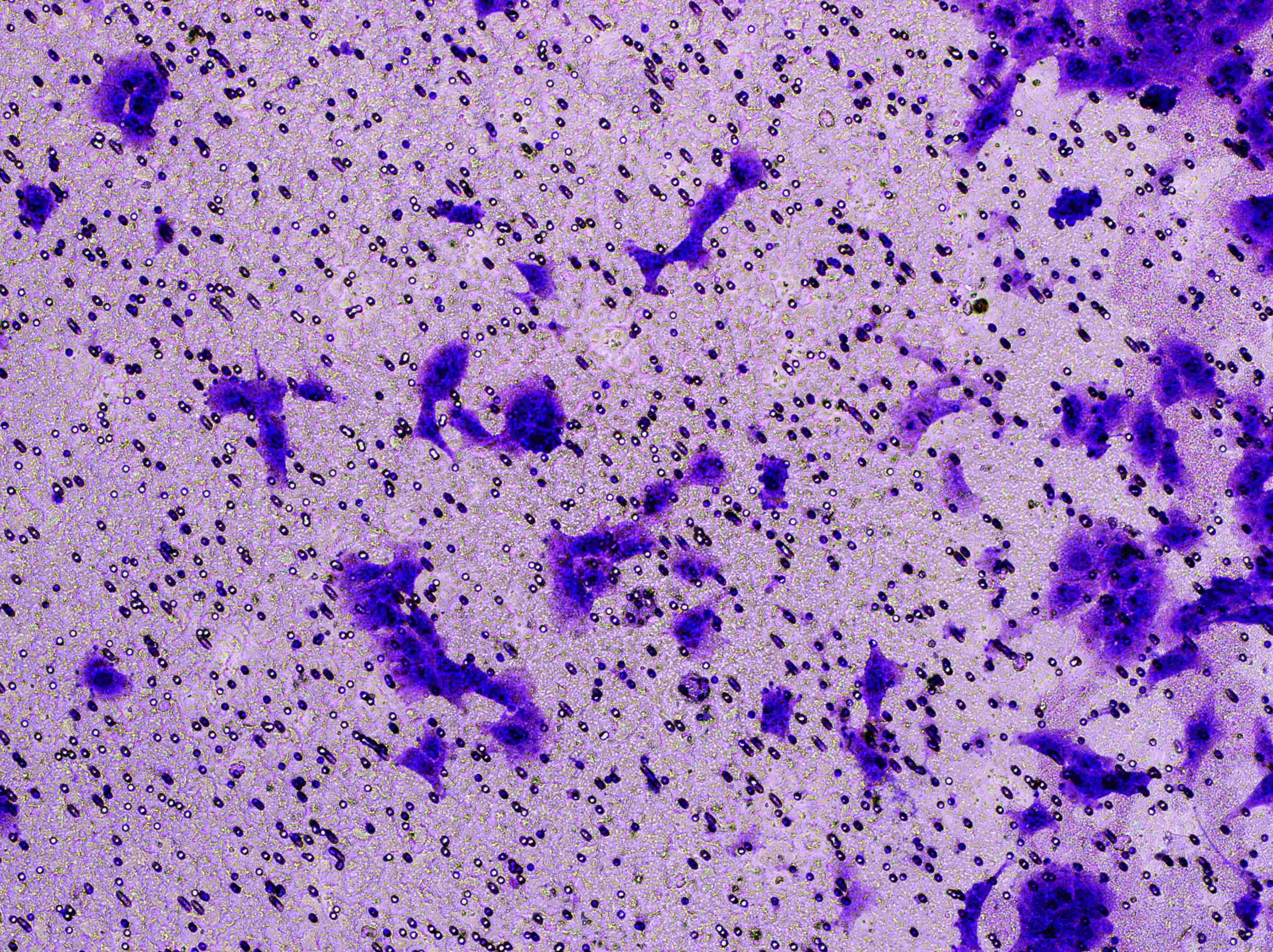

Supplement: Supplementary file 4 [file DataSheet2.ZIP › raw data2/Figur3G 5E 6D transwell/miR-185/185 mimic2.jpg]

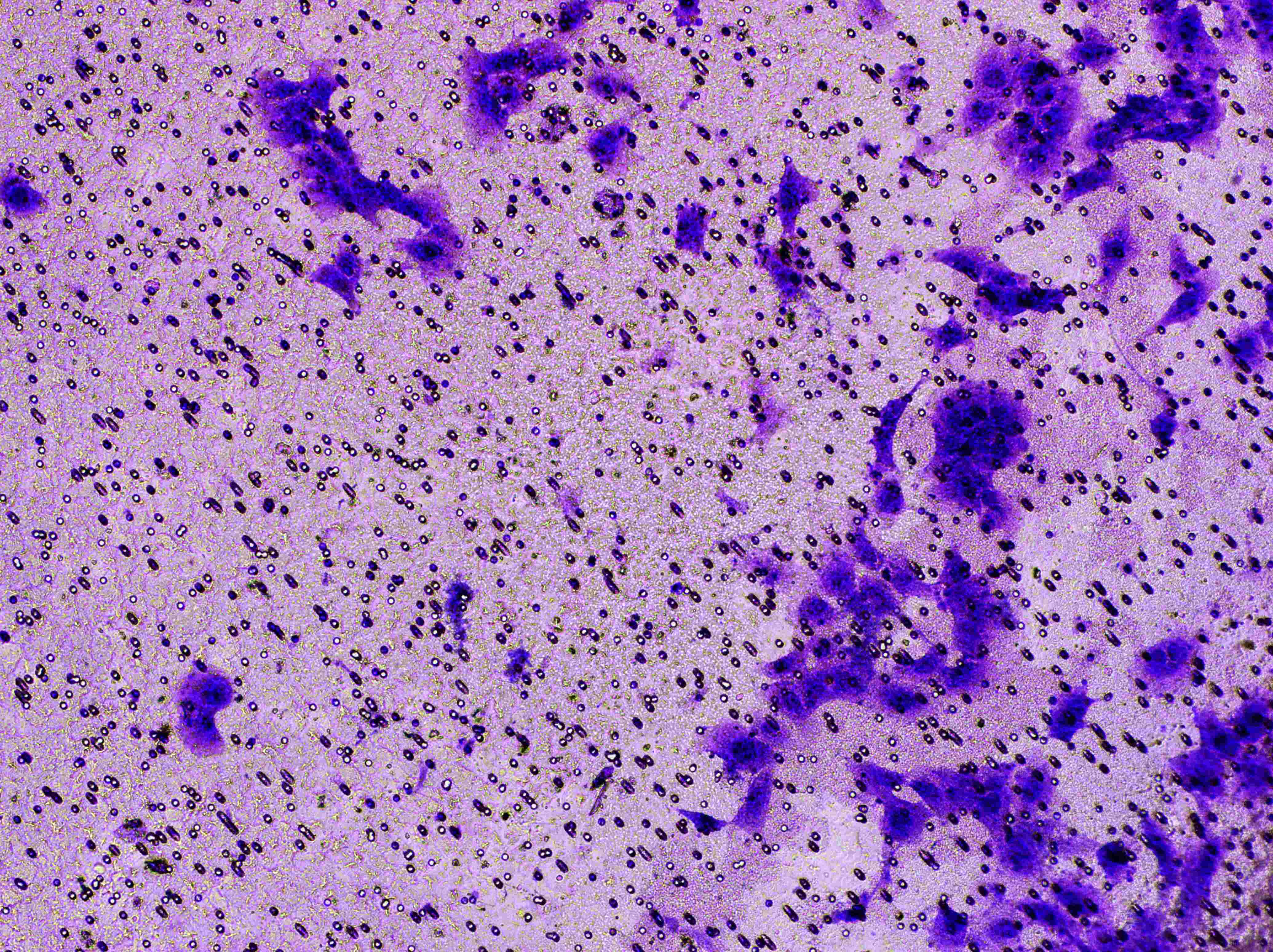

Supplement: Supplementary file 4 [file DataSheet2.ZIP › raw data2/Figur3G 5E 6D transwell/miR-185/185 mimic3.jpg]

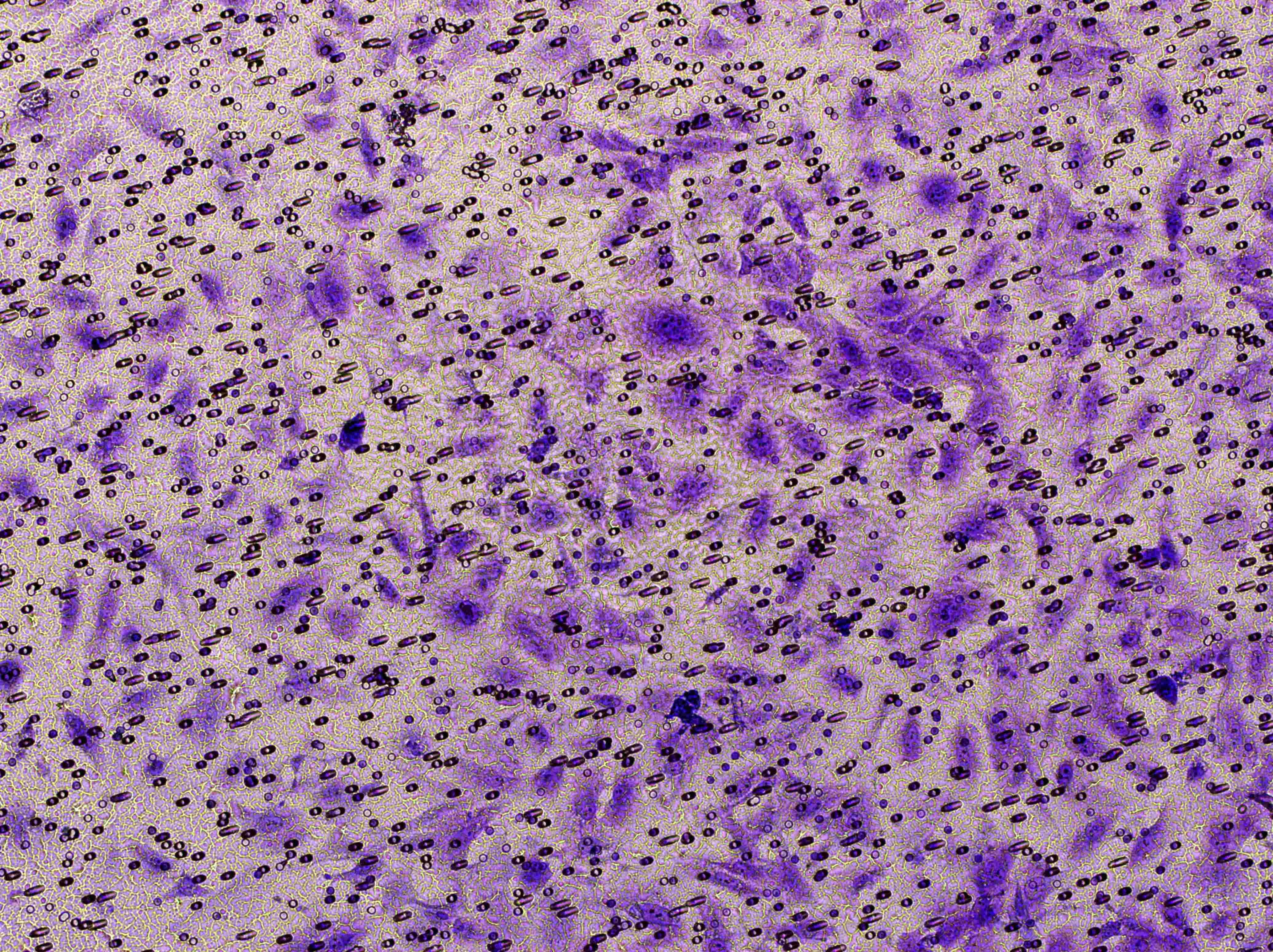

Supplement: Supplementary file 4 [file DataSheet2.ZIP › raw data2/Figur3G 5E 6D transwell/rescue/LV-KD+185 inhibitor 1.jpg]

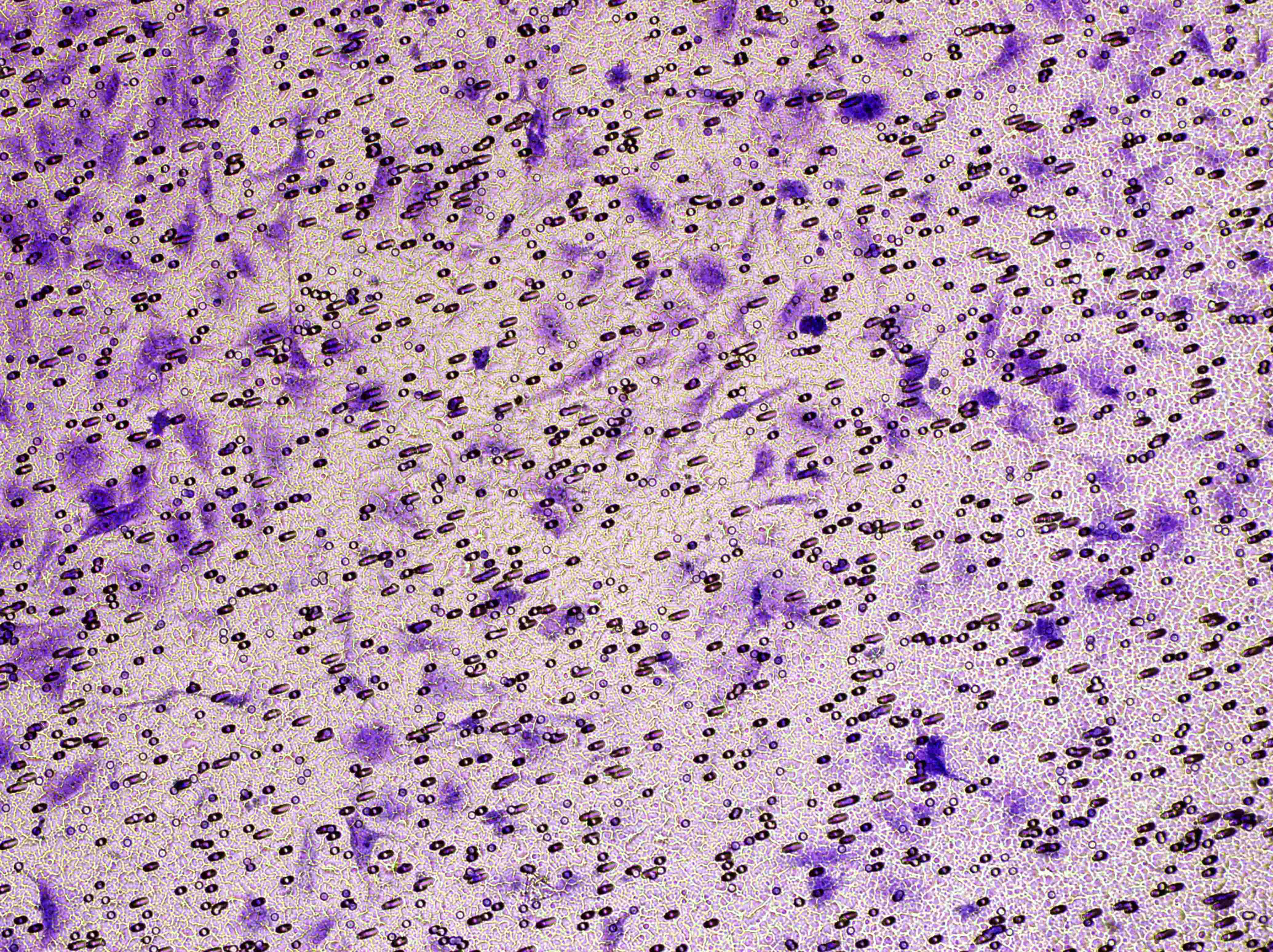

Supplement: Supplementary file 4 [file DataSheet2.ZIP › raw data2/Figur3G 5E 6D transwell/rescue/LV-KD+185 inhibitor 2.jpg]

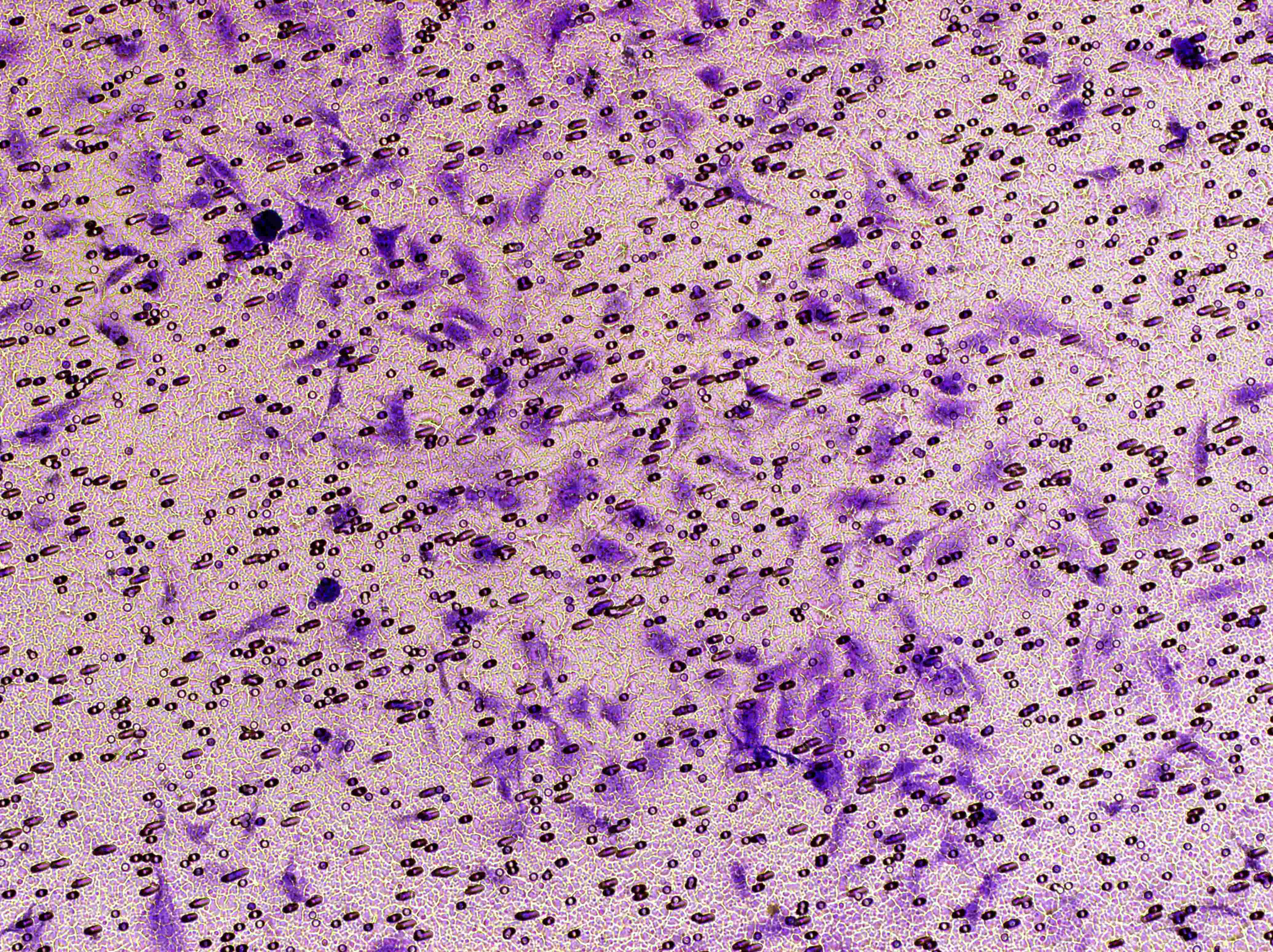

Supplement: Supplementary file 4 [file DataSheet2.ZIP › raw data2/Figur3G 5E 6D transwell/rescue/LV-KD+185 inhibitor 3.jpg]

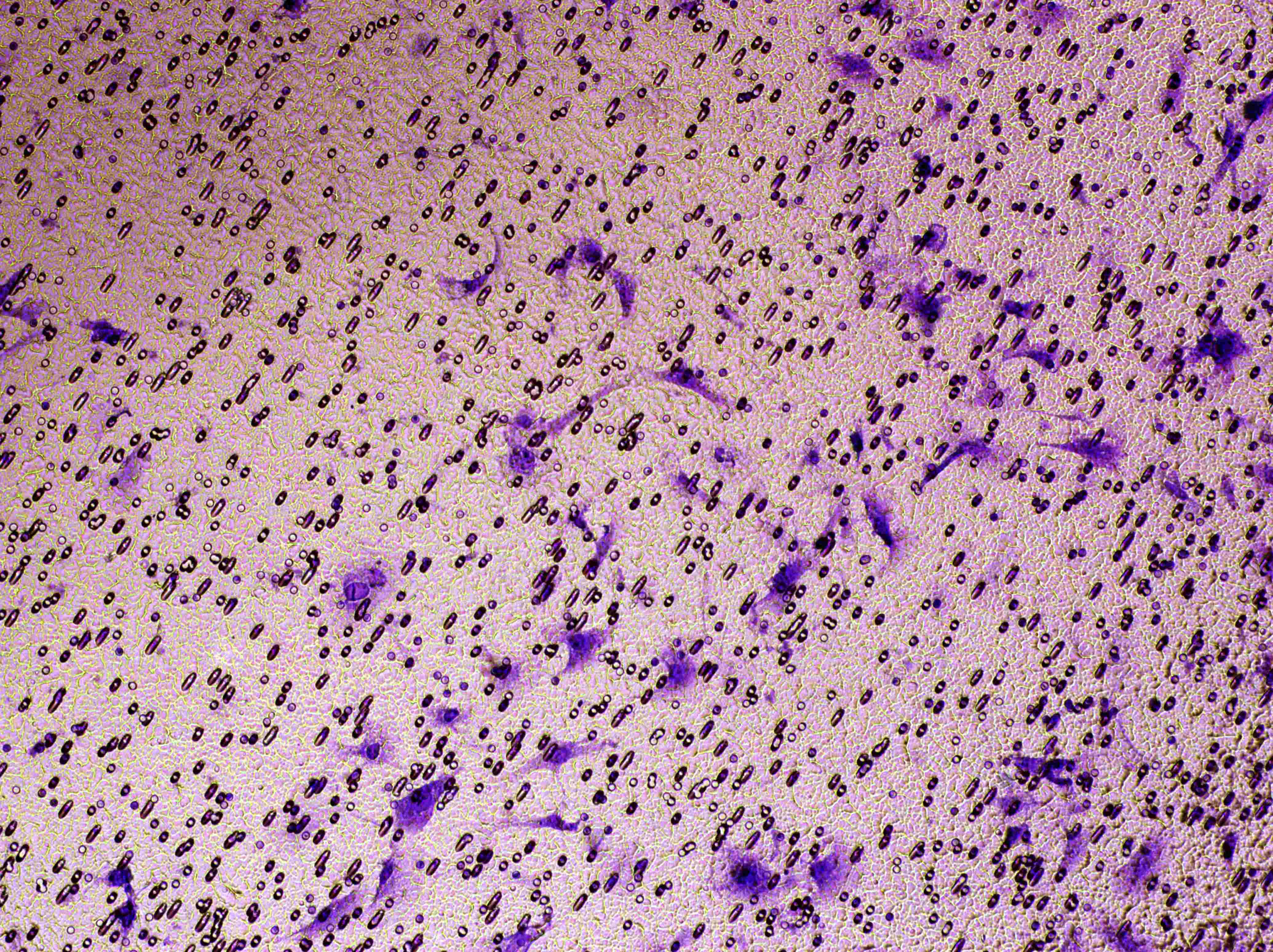

Supplement: Supplementary file 4 [file DataSheet2.ZIP › raw data2/Figur3G 5E 6D transwell/rescue/LV-KD+inhibitor NC1.jpg]

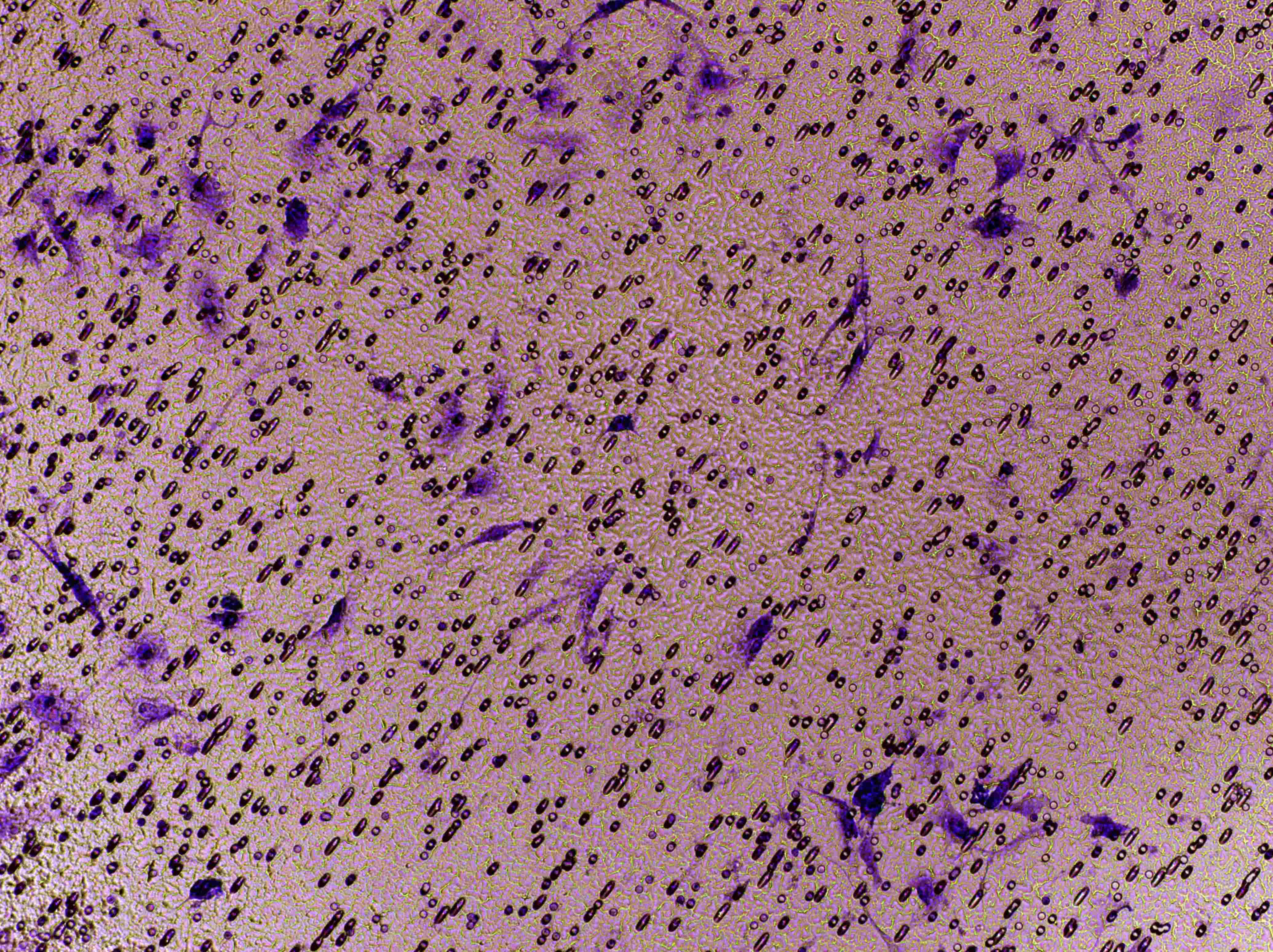

Supplement: Supplementary file 4 [file DataSheet2.ZIP › raw data2/Figur3G 5E 6D transwell/rescue/LV-KD+inhibitor NC2.jpg]

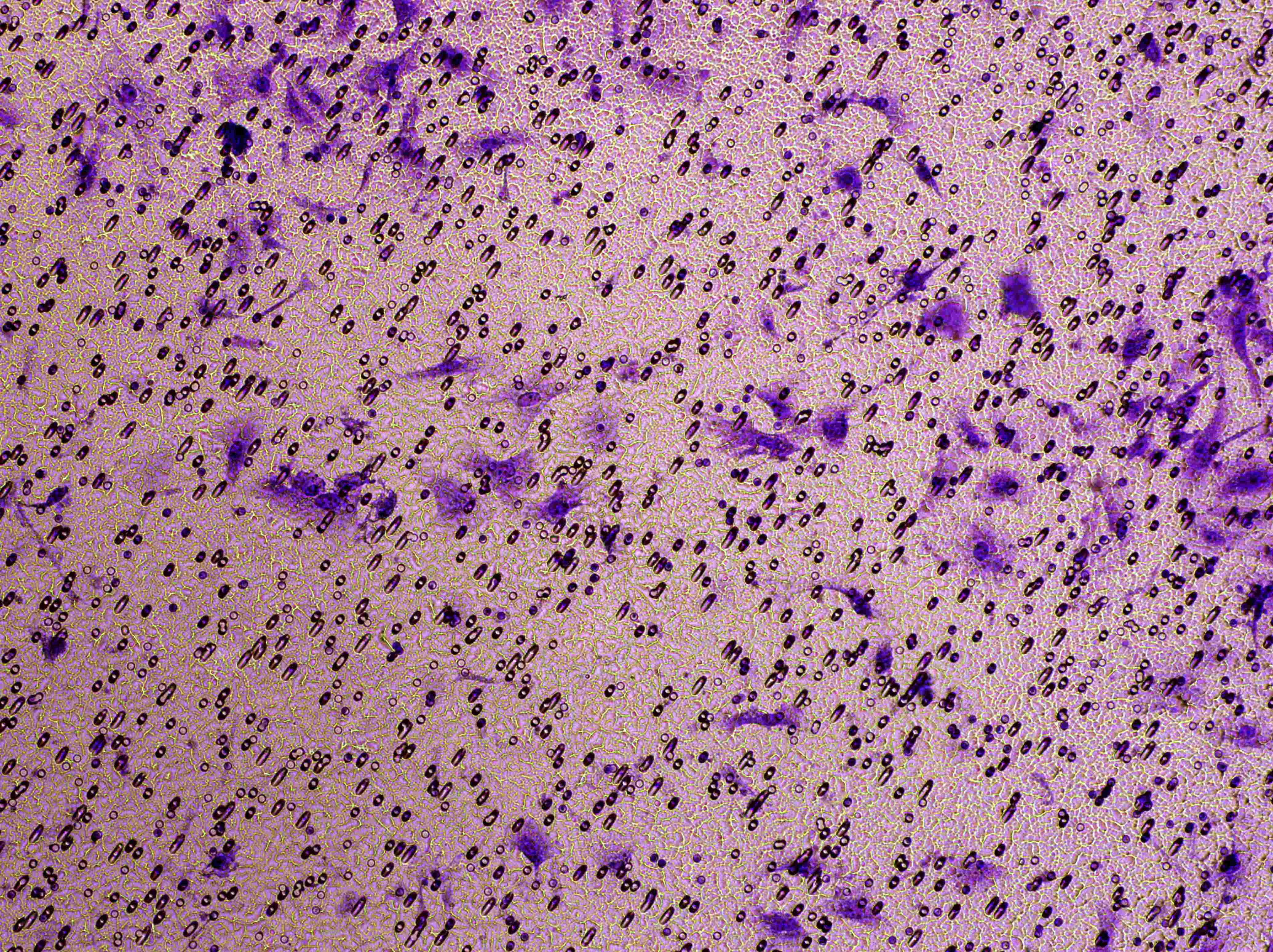

Supplement: Supplementary file 4 [file DataSheet2.ZIP › raw data2/Figur3G 5E 6D transwell/rescue/LV-KD+inhibitor NC3.jpg]

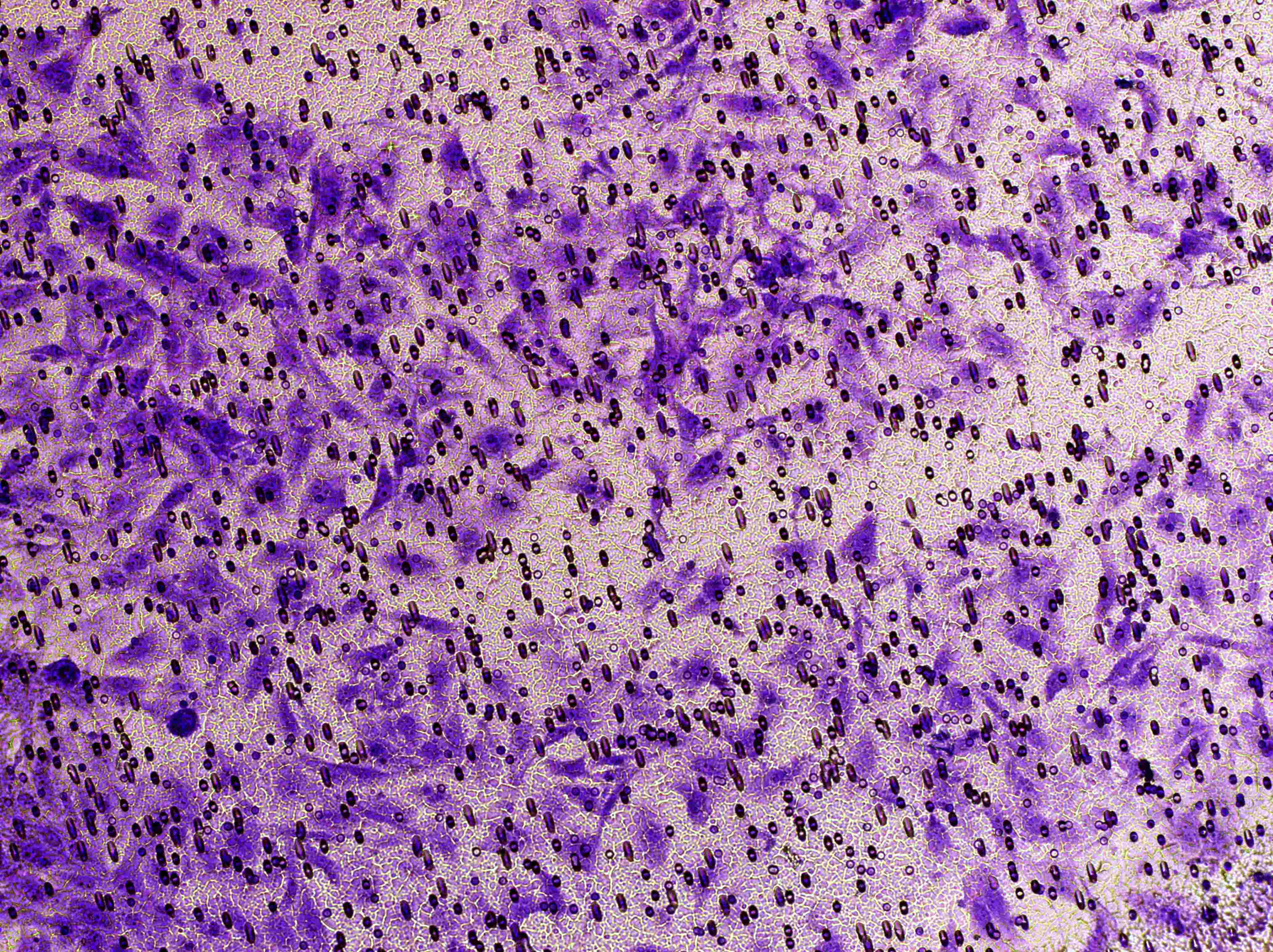

Supplement: Supplementary file 4 [file DataSheet2.ZIP › raw data2/Figur3G 5E 6D transwell/rescue/LV-NC+inhibitor NC1.jpg]

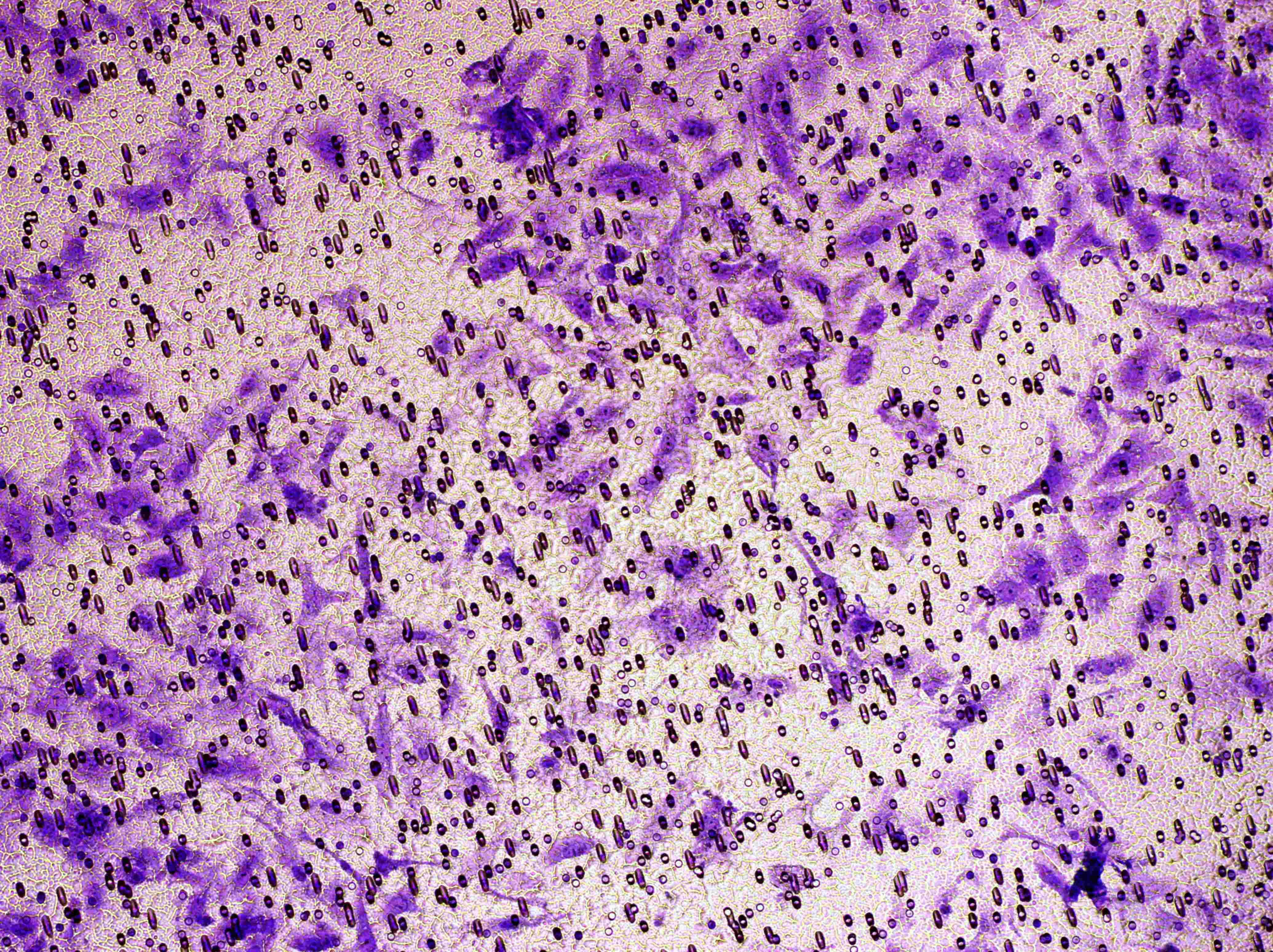

Supplement: Supplementary file 4 [file DataSheet2.ZIP › raw data2/Figur3G 5E 6D transwell/rescue/LV-NC+inhibitor NC2.jpg]

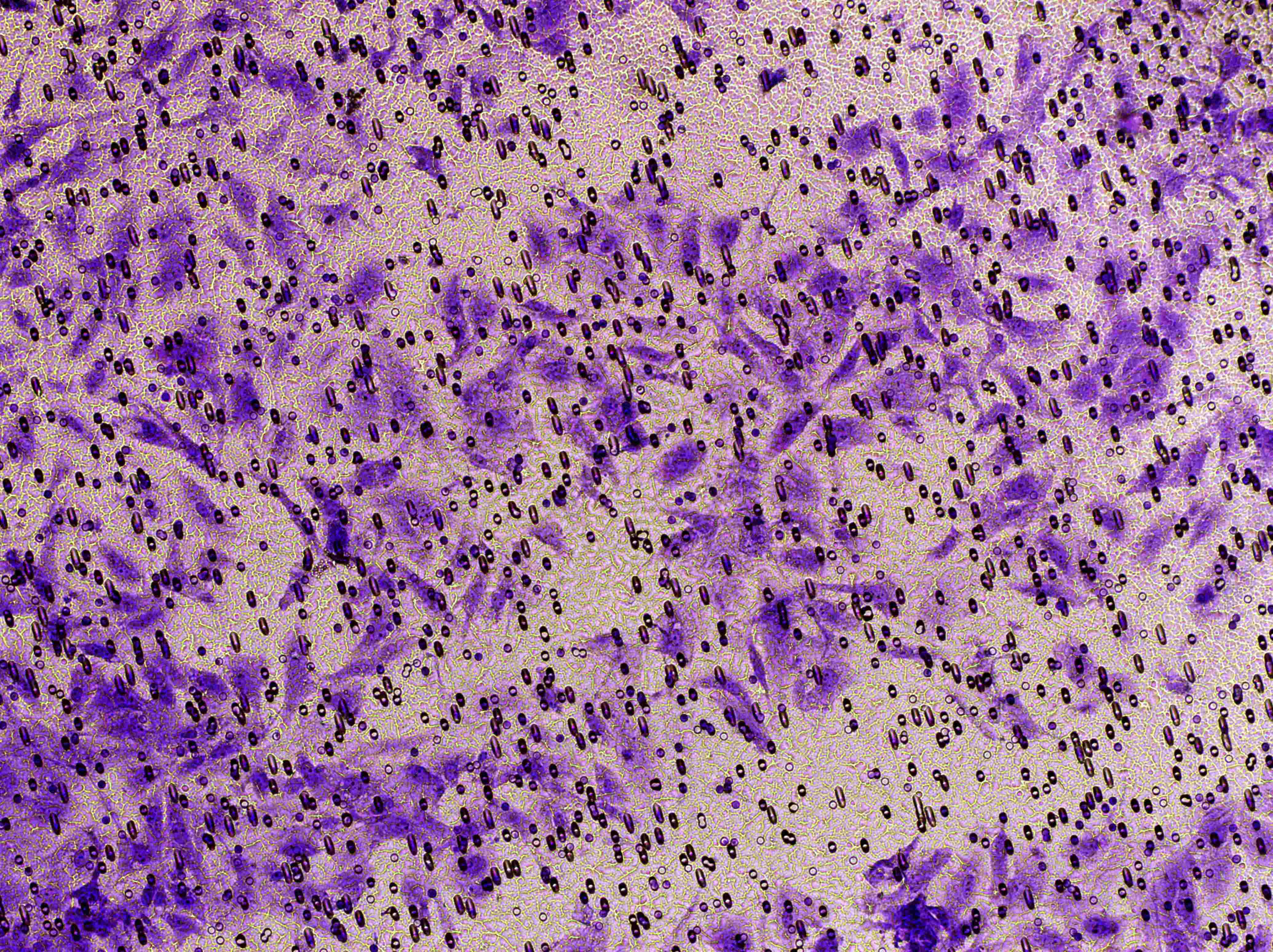

Supplement: Supplementary file 4 [file DataSheet2.ZIP › raw data2/Figur3G 5E 6D transwell/rescue/LV-NC+inhibitor NC3.jpg]

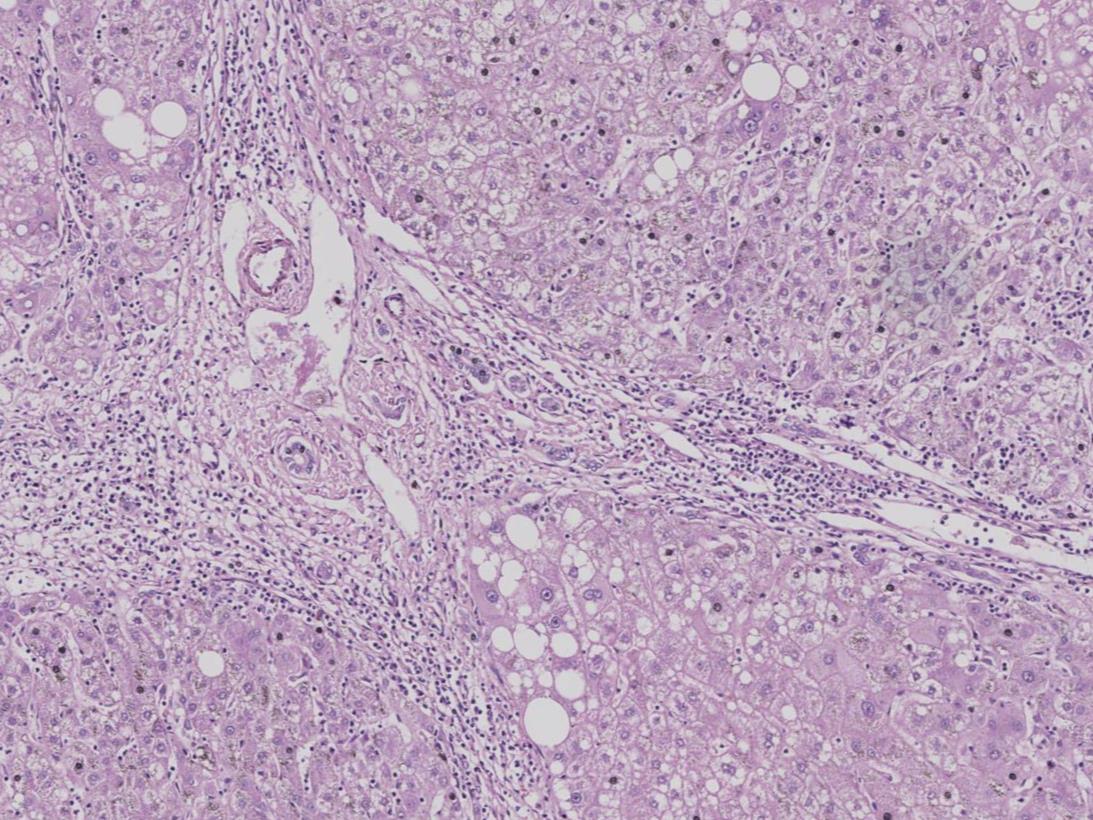

Supplement: Supplementary file 4 [file DataSheet2.ZIP › raw data2/Figure2G and supple Figure2 FISH/HE MASSON IHC/HE 2022-06-17 08_18_10_10.00X_20220626124227.jpg]

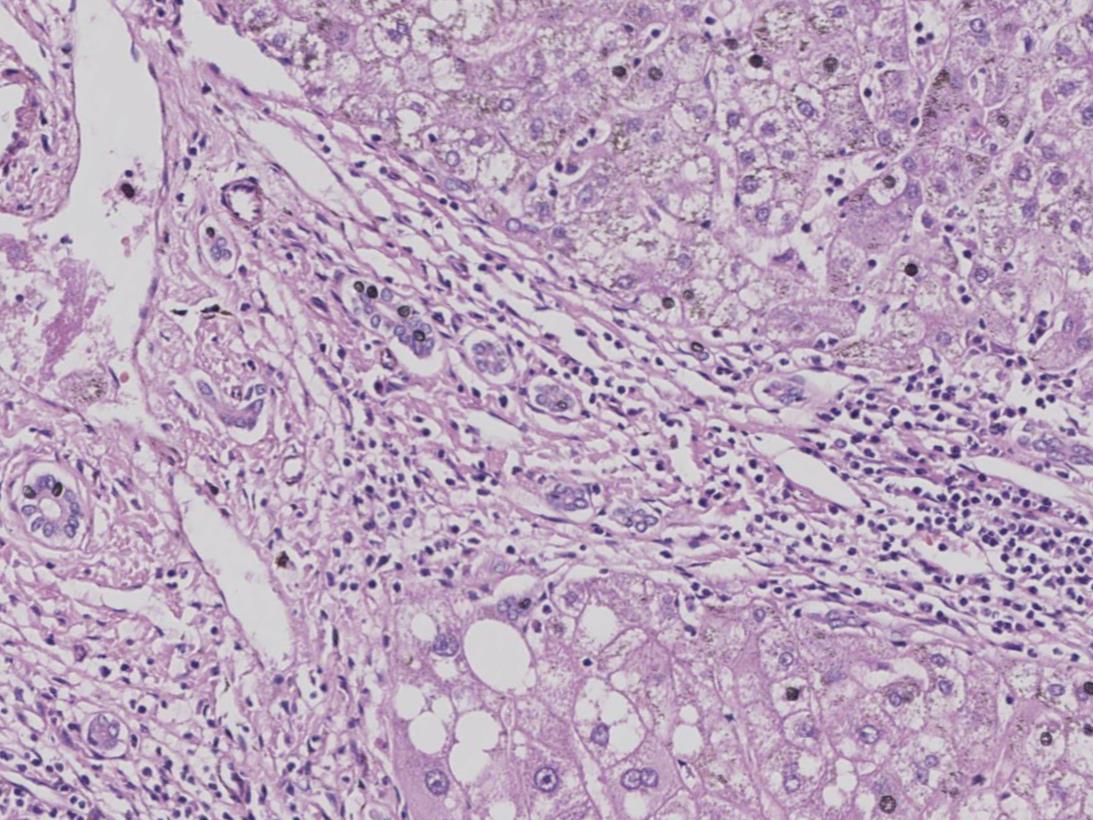

Supplement: Supplementary file 4 [file DataSheet2.ZIP › raw data2/Figure2G and supple Figure2 FISH/HE MASSON IHC/HE 2022-06-17 08_18_10_20.00X_20220626124221.jpg]

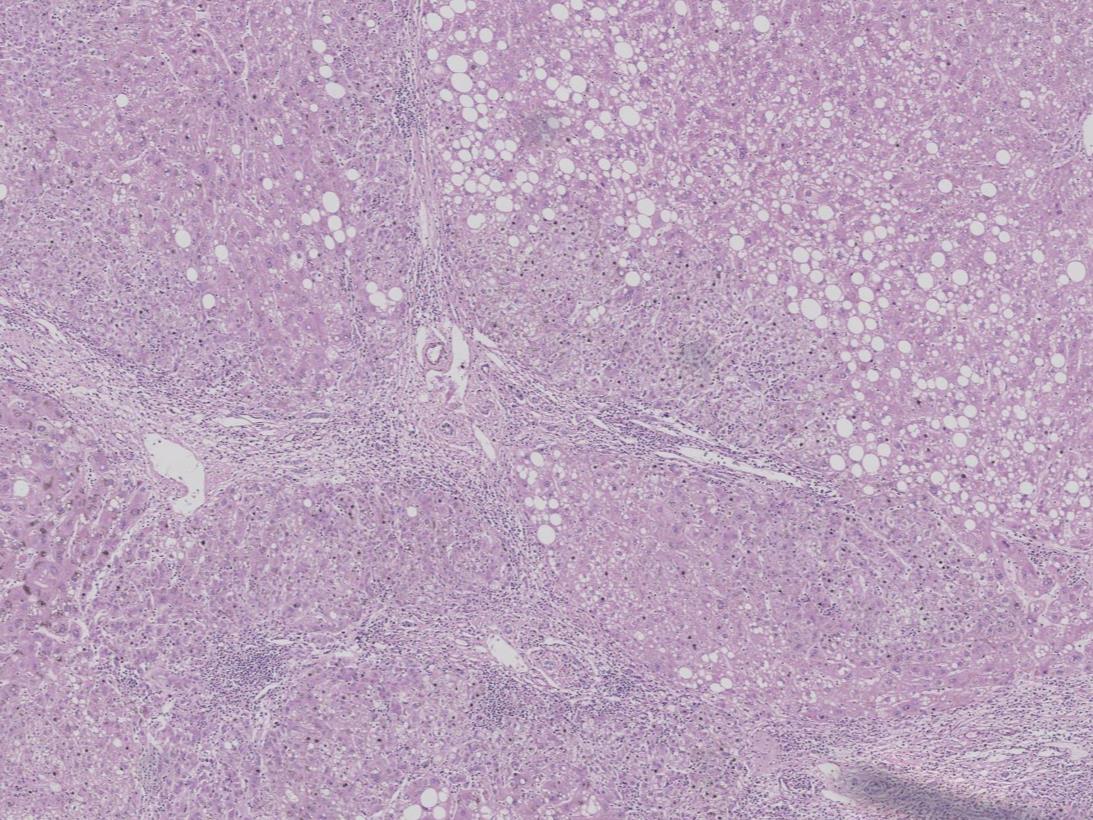

Supplement: Supplementary file 4 [file DataSheet2.ZIP › raw data2/Figure2G and supple Figure2 FISH/HE MASSON IHC/HE 2022-06-17 08_18_10_4.00X_20220626124233.jpg]

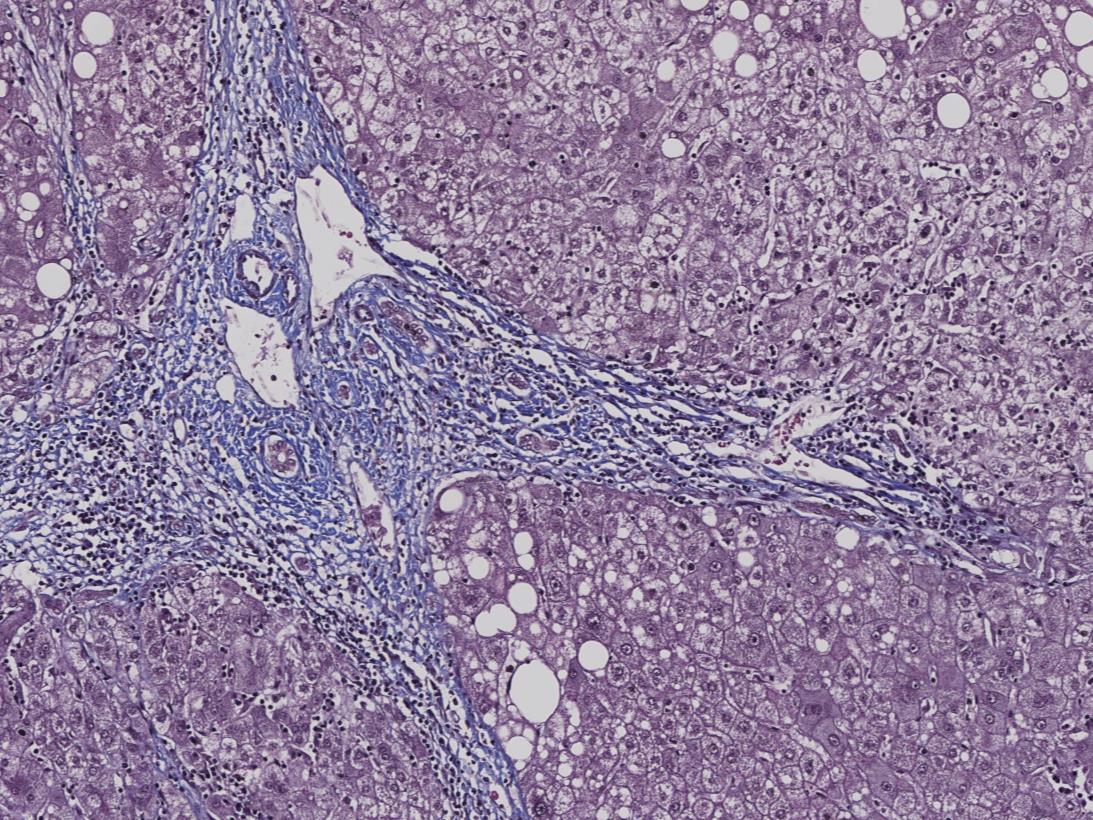

Supplement: Supplementary file 4 [file DataSheet2.ZIP › raw data2/Figure2G and supple Figure2 FISH/HE MASSON IHC/Masson 2022-06-17 08_28_10_10.00X_20220626124020.jpg]

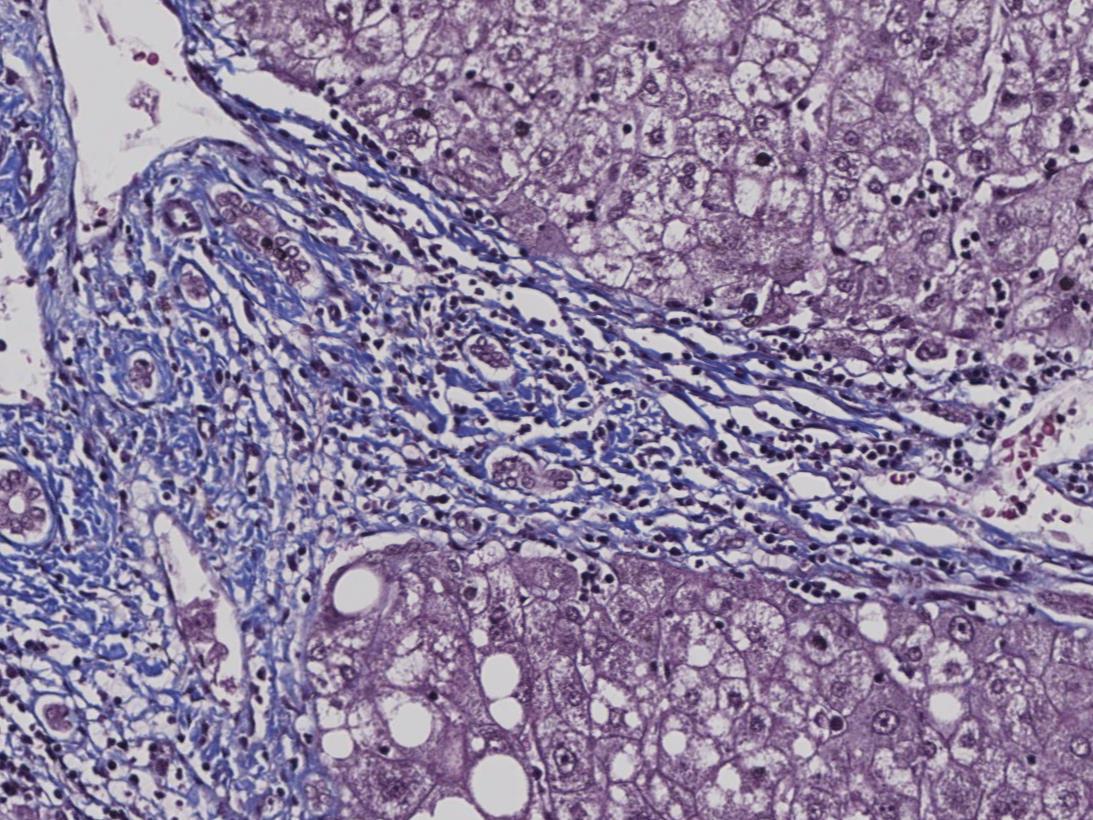

Supplement: Supplementary file 4 [file DataSheet2.ZIP › raw data2/Figure2G and supple Figure2 FISH/HE MASSON IHC/Masson 2022-06-17 08_28_10_20.00X_20220626124036.jpg]

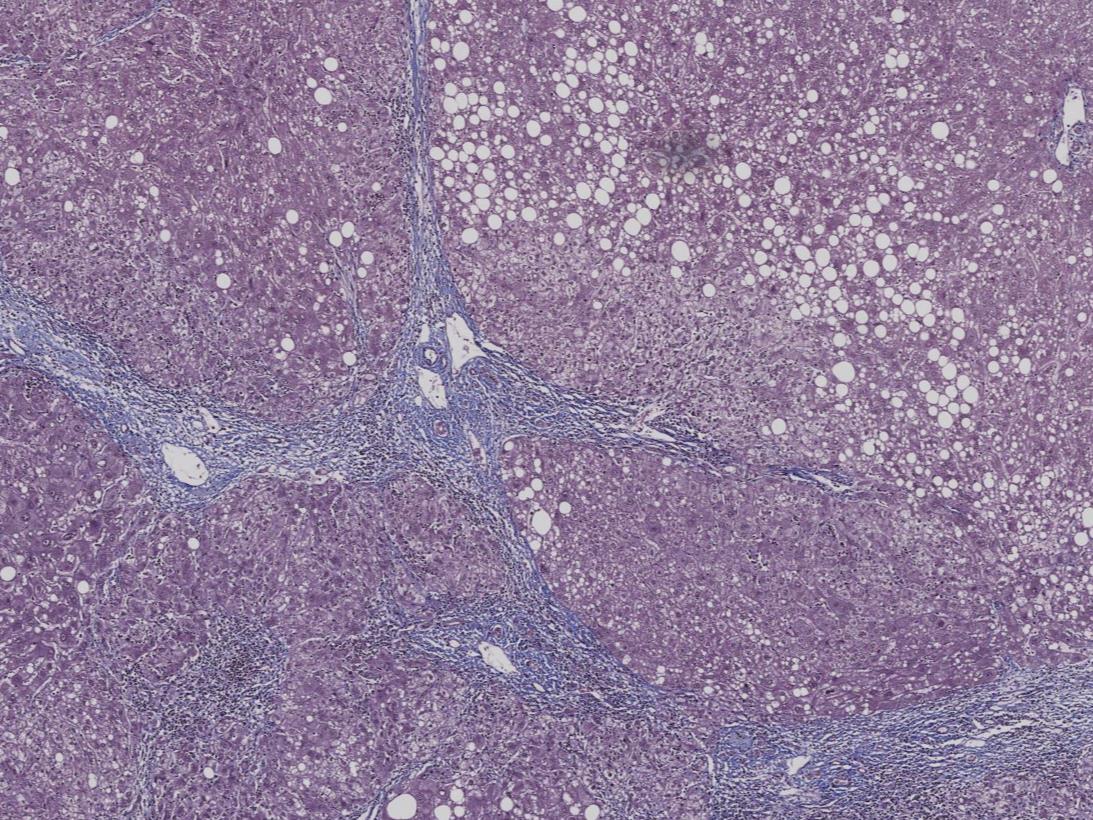

Supplement: Supplementary file 4 [file DataSheet2.ZIP › raw data2/Figure2G and supple Figure2 FISH/HE MASSON IHC/Masson 2022-06-17 08_28_10_4.00X_20220626124029.jpg]

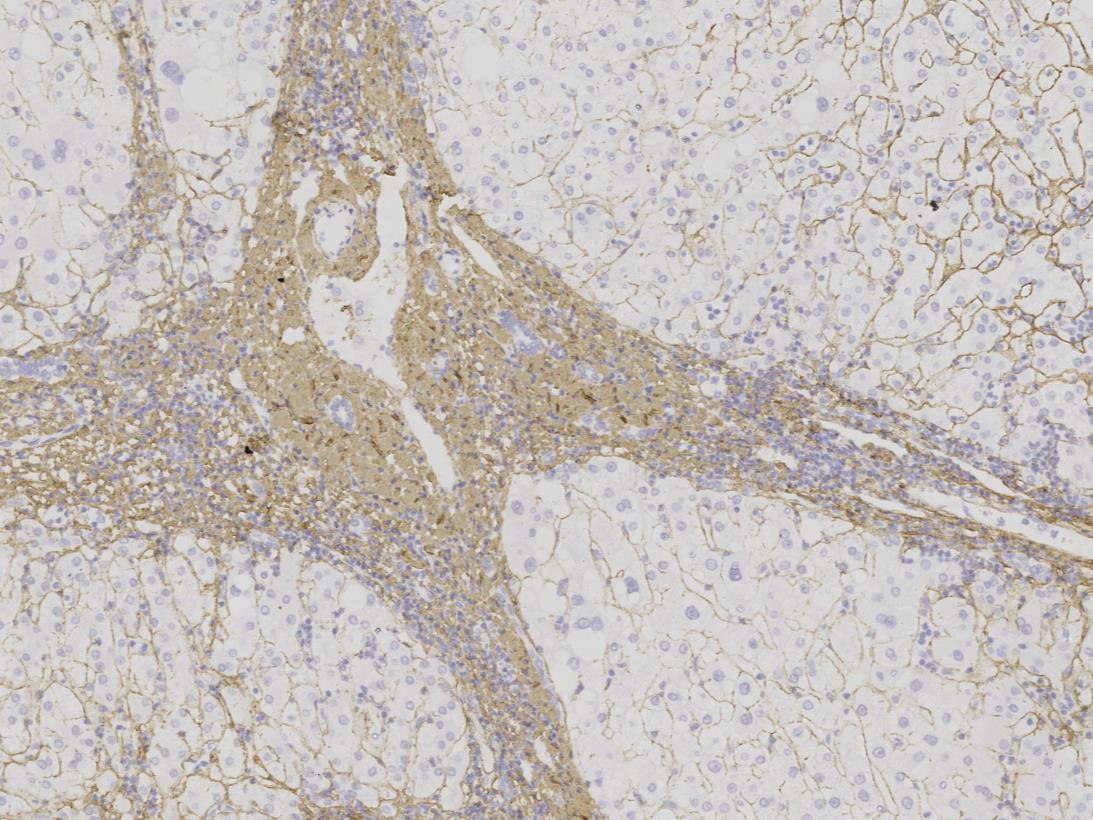

Supplement: Supplementary file 4 [file DataSheet2.ZIP › raw data2/Figure2G and supple Figure2 FISH/HE MASSON IHC/col 2022-06-27 14_49_59_10.00X_20220627084604.jpg]

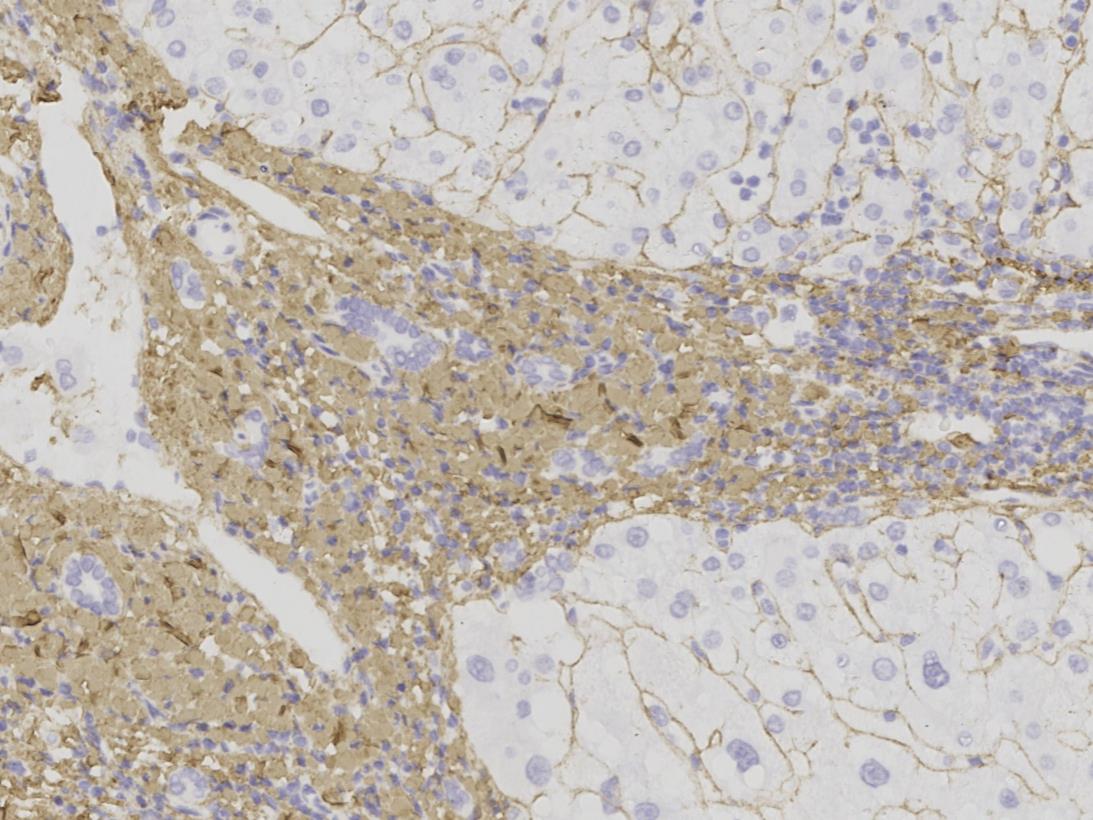

Supplement: Supplementary file 4 [file DataSheet2.ZIP › raw data2/Figure2G and supple Figure2 FISH/HE MASSON IHC/col 2022-06-27 14_49_59_20.00X_20220628071402.jpg]

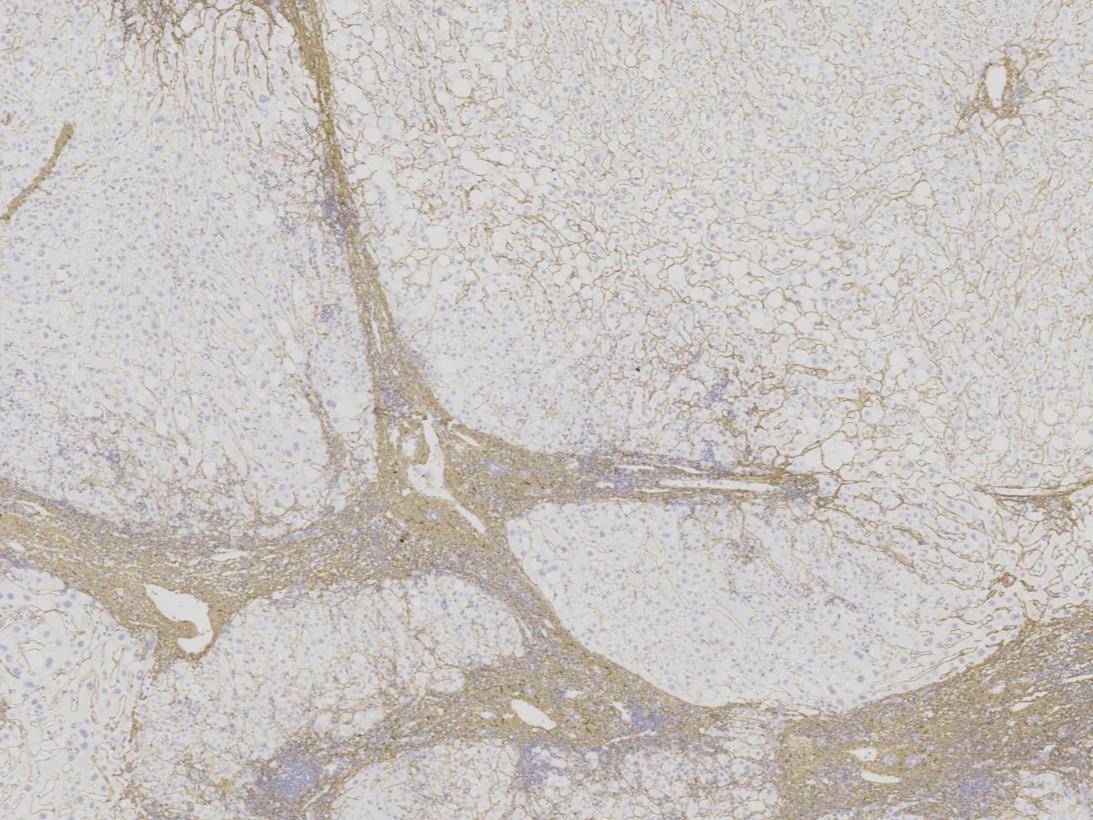

Supplement: Supplementary file 4 [file DataSheet2.ZIP › raw data2/Figure2G and supple Figure2 FISH/HE MASSON IHC/col 2022-06-27 14_49_59_4.00X_20220628071002.jpg]

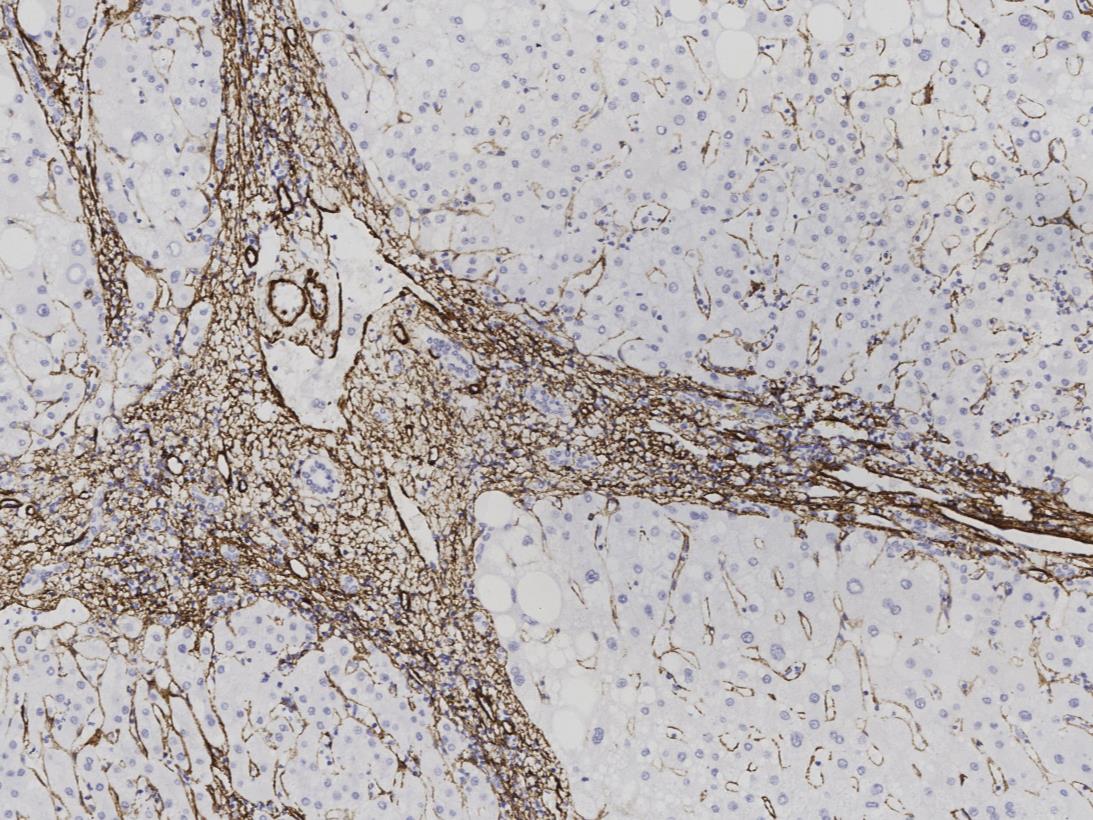

Supplement: Supplementary file 4 [file DataSheet2.ZIP › raw data2/Figure2G and supple Figure2 FISH/HE MASSON IHC/sma 2022-06-17 09_23_44_10.00X_20220626123847.jpg]

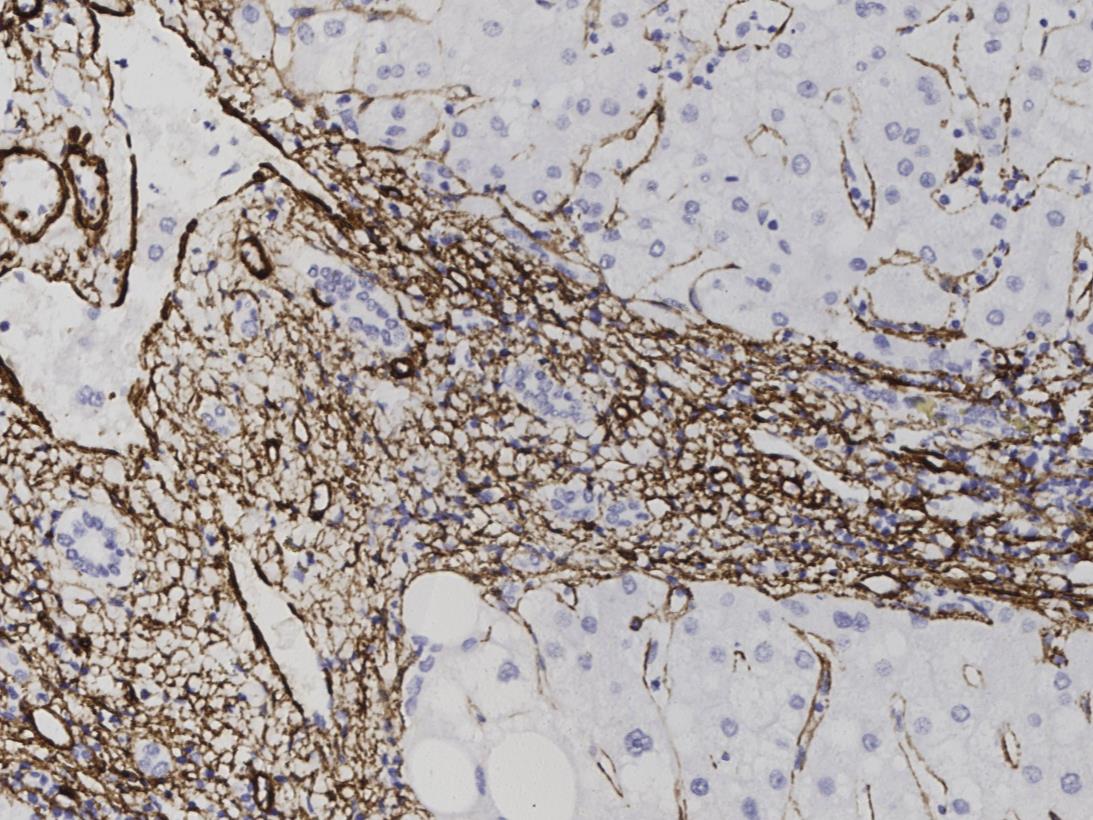

Supplement: Supplementary file 4 [file DataSheet2.ZIP › raw data2/Figure2G and supple Figure2 FISH/HE MASSON IHC/sma 2022-06-17 09_23_44_20.00X_20220626123839.jpg]

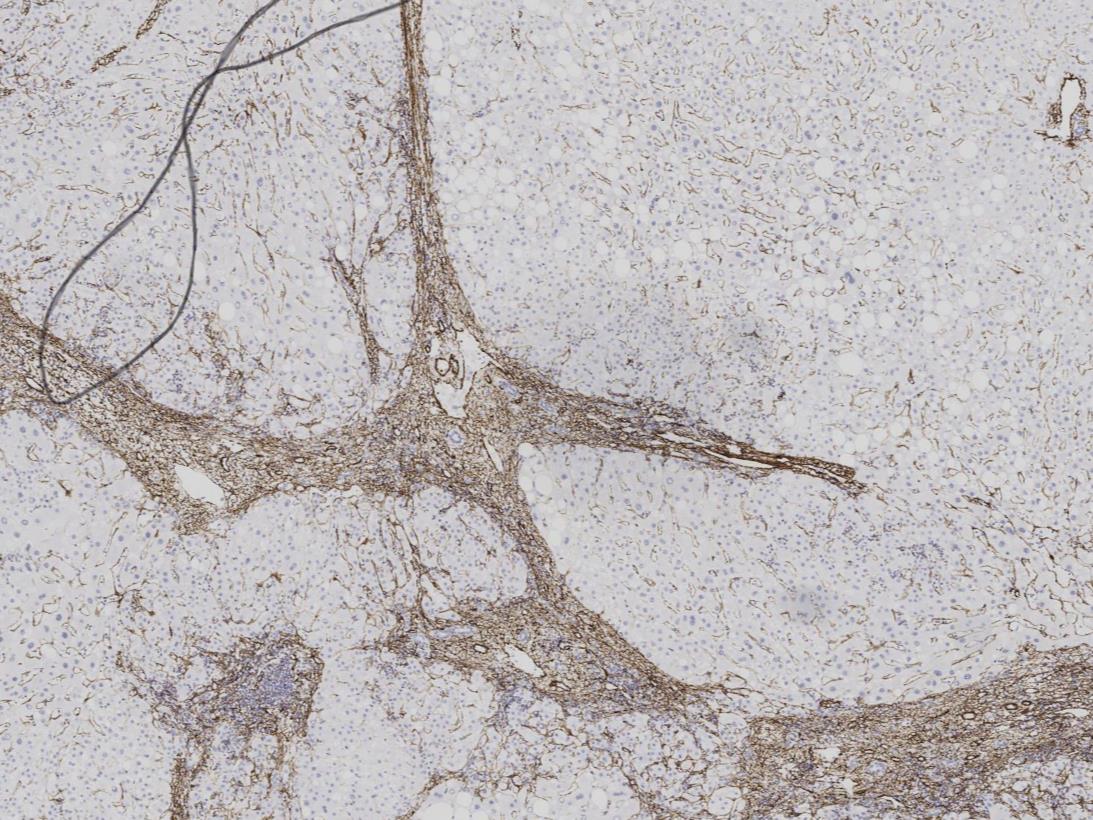

Supplement: Supplementary file 4 [file DataSheet2.ZIP › raw data2/Figure2G and supple Figure2 FISH/HE MASSON IHC/sma 2022-06-17 09_23_44_4.00X_20220626123856.jpg]

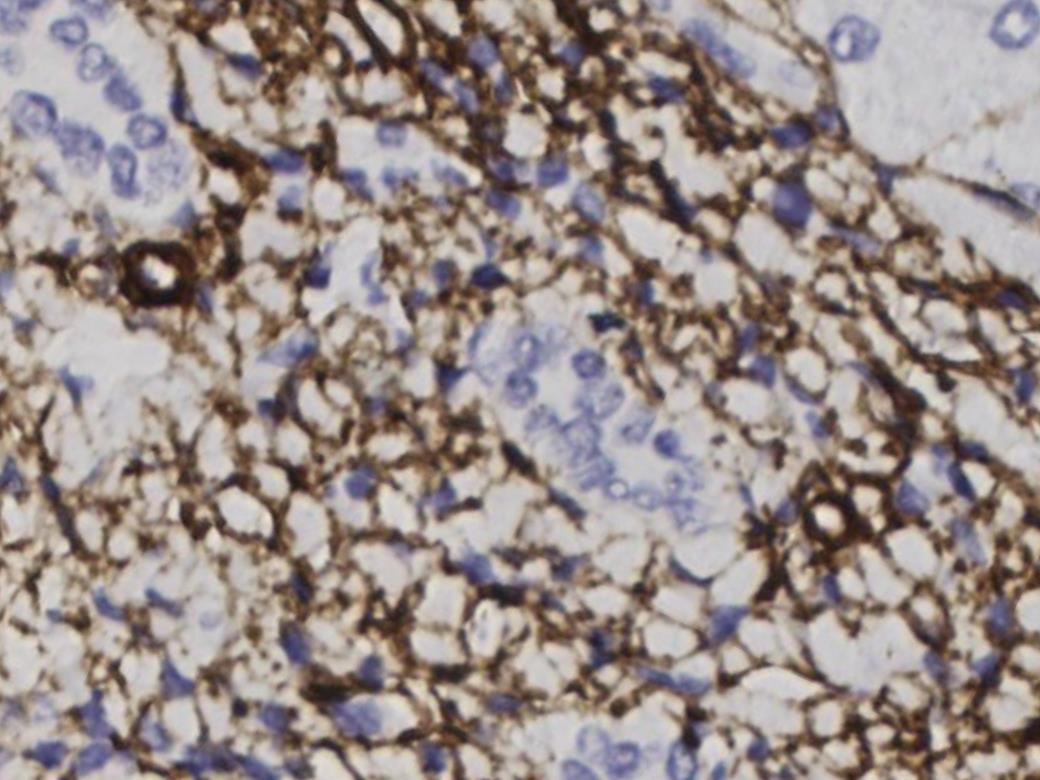

Supplement: Supplementary file 4 [file DataSheet2.ZIP › raw data2/Figure2G and supple Figure2 FISH/HE MASSON IHC/sma 2022-06-17 09_23_44_40.00X_20220630112314.jpg]

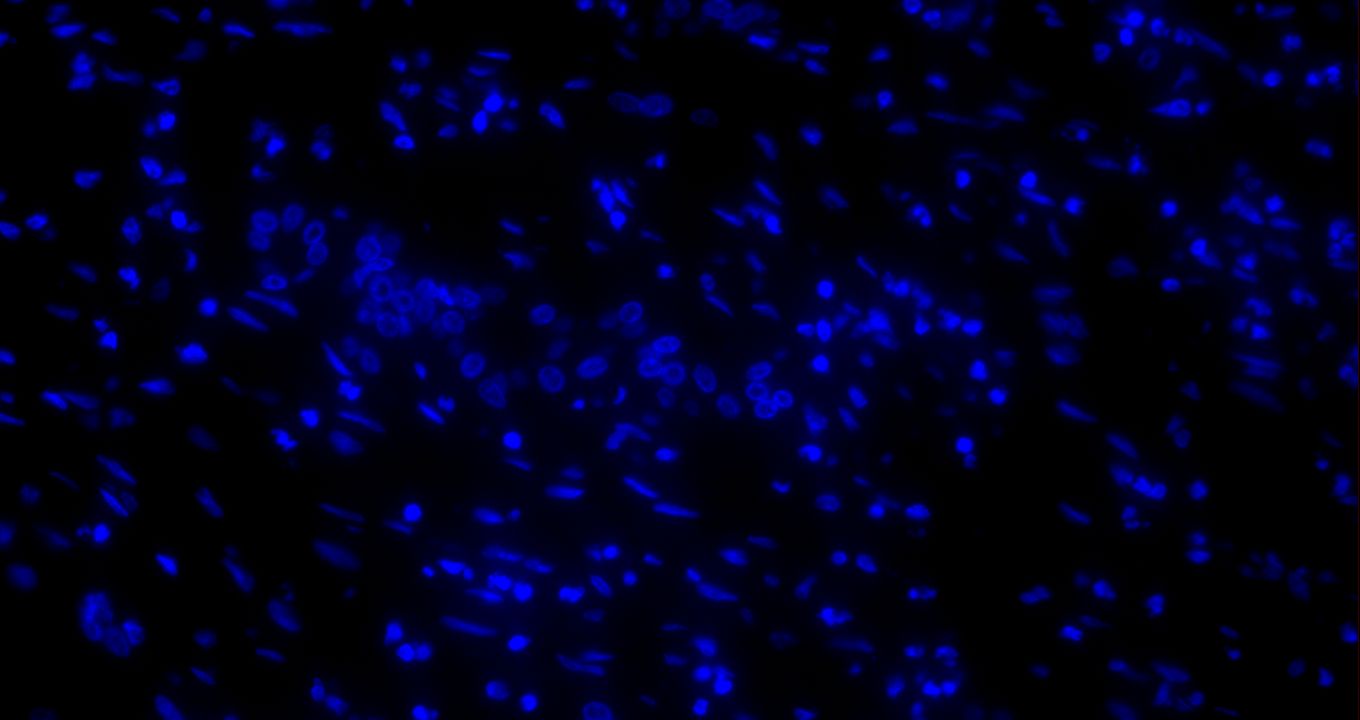

Supplement: Supplementary file 4 [file DataSheet2.ZIP › raw data2/Figure2G and supple Figure2 FISH/fish/8494 dapi.JPG]

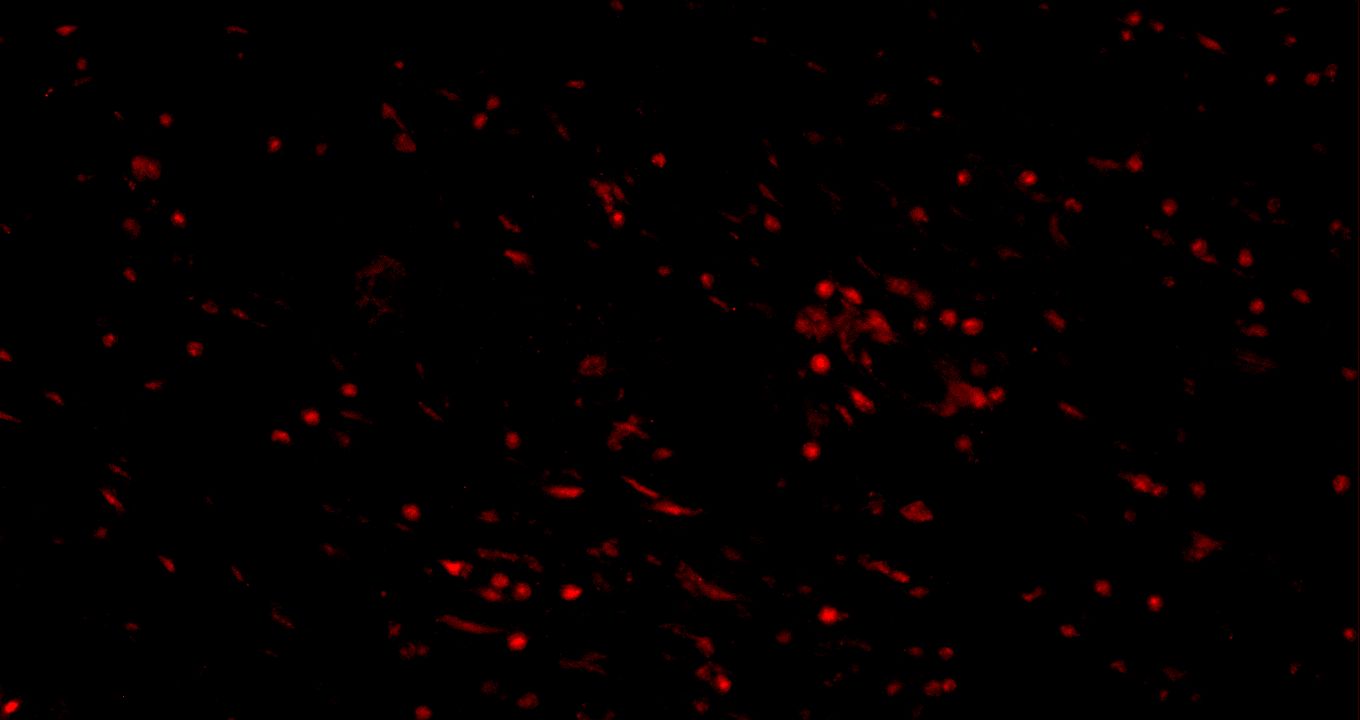

Supplement: Supplementary file 4 [file DataSheet2.ZIP › raw data2/Figure2G and supple Figure2 FISH/fish/8494 luo.JPG]

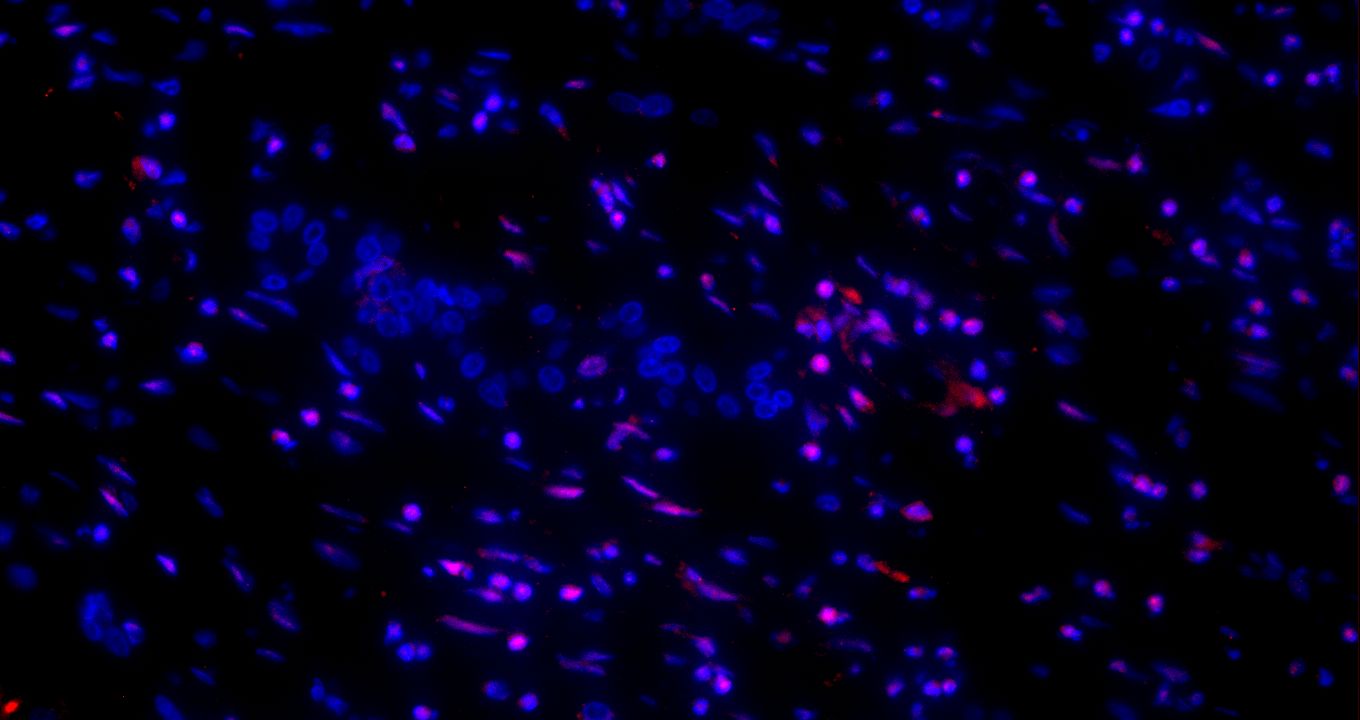

Supplement: Supplementary file 4 [file DataSheet2.ZIP › raw data2/Figure2G and supple Figure2 FISH/fish/8494 merger.JPG]
